# Therapeutic Vaccination with TNF-Kinoid in TNF Antagonist-Resistant Rheumatoid Arthritis: A Phase II Randomized, Controlled Clinical Trial

**DOI:** 10.1371/journal.pone.0113465

**Published:** 2014-12-17

**Authors:** Patrick Durez, Pierre Vandepapeliere, Pedro Miranda, Antoaneta Toncheva, Alberto Berman, Tatjana Kehler, Eugenia Mociran, Bruno Fautrel, Xavier Mariette, Olivier Dhellin, Bernard Fanget, Stephane Ouary, Géraldine Grouard-Vogel, Marie-Christophe Boissier

**Affiliations:** 1 Service et Pole de Rhumatologie, Cliniques Universitaires Saint-Luc, Brussels, Belgium; Institut de Recherche Experimentale et Clinique, Universite catholique de Louvain, Rheumatology, Brussels, Belgium; 2 Neovacs S.A., Paris, France; 3 Centro de Estudios Reumatológicos, Santiago de Chile, Chile; 4 National Multiprofile Transport Hospital “Tzar Boris III”, Sofia, Bulgaria; 5 Centro Medico Privado de Reumatologia, San Miguel de Tucuman, Argentina; 6 Thalassotherapia Opatija, Opatija, Croatia; 7 Emergency County Hospital “Dr Constantin Opris”, Baia Mare, Romania; 8 UPMC, GRC 08 (EEMOIS), Institut Pierre Louis d’Epidémiologie et Santé Publique, Paris, France; 9 AP-HP, Pitié Salpêtrière University Hospital, Dept of Rheumatology, Paris, France; 10 Université Paris-Sud, Hôpitaux Universitaires Paris-Sud, AP-HP, INSERM U1012, Le Kremlin-Bicêtre, France; 11 INSERM U1125, Bobigny, France; 12 Université Paris 13, Sorbonne Paris Cité, Bobigny, France; 13 AP-HP, Avicenne University Hospital, Department of Rheumatology, Bobigny, France; Immunology Frontier Research Center, Osaka University, Japan

## Abstract

**Objectives:**

Active immunization, or vaccination, with tumor necrosis factor (TNF)-Kinoid (TNF-K) is a novel approach to induce polyclonal anti-TNF antibodies in immune-mediated inflammatory diseases. This study was performed to transfer the proof of concept obtained in mice model of rheumatoid arthritis (RA) into human. We designed a pilot study to demonstrate the feasibility of therapeutic vaccination in RA.

**Methods:**

This was a phase IIa, placebo-controlled, multicenter study in adults with RA who previously experienced secondary failure of TNF antagonists. Patients were immunized intramuscularly with 2 or 3 doses of placebo (n = 10) or 90 (n = 6), 180 (n = 12), or 360 µg TNF-K (n = 12). The primary objective was to identify the best dose and schedule based on anti-TNF antibody titers. Clinical symptoms and safety were assessed during 12 months and solicited reactions for 7 days after each injection.

**Results:**

The highest anti-TNF antibody response was detected in patients immunized with 360 µg TNF-K and with 3 injections, although this difference was not significant with all other groups. Similar proportions of patients receiving TNF-K and placebo reported adverse events up to month 12. Serious adverse events were reported by 4 patients treated with TNF-K (13.3%) and 3 treated with placebo (30.0%), all unrelated to treatment. At month 12, DAS28-CRP, tender and swollen joint counts, and HAQ scores decreased significantly more in patients who exhibited anti-TNF antibody response than in patients who did not.

**Conclusions:**

TNF-K therapeutic vaccination induced dose- and schedule-dependent anti-TNF antibodies in RA patients and was well tolerated. Patients who developed anti-TNF antibodies showed a trend toward clinical improvement. Although the most aggressive dose and schedule, i.e. 360 mg dose administered 3 times, did show a strong trend of higher antibody response, further studies are warranted to examine even higher and more frequent doses in order to establish the best conditions for clinical improvement.

**Trial Registration:**

ClinicalTrials.gov NCT01040715

## Introduction

Rheumatoid arthritis (RA) is a chronic systemic immune mediated inflammatory disease that affects 0.5 to 1% of the population, resulting in major functional disability and increased mortality [1]. Hyperproduction of tumor necrosis factor (TNF) produced by activated monocytes and macrophages plays a central role in RA, resulting in synovitis and articular matrix degradation [2]. The advent of TNF targeting drugs (anti-TNF) have changed dramatically the perspectives of RA treatment over the last decade, with unprecedented results in terms of disease control and articular destruction prevention [3]. Nevertheless, only 30 to 40% of anti-TNF treated patients achieved remission in controlled clinical trials [4], and even lower remission rates are described in everyday practice [5]. An approximately similar proportion reaches a functional status comparable to that of the general population [6]. Primary or secondary therapeutic failures on anti-TNF drugs are frequent [7], and there is now evidence that the induction of anti-drug antibodies could be a major factor to loss of response to this class of therapeutics, mainly with the use of anti-TNF monoclonal antibodies [8], [9]. These drawbacks of current anti-TNF treatments confirm that there is room for alternative ways to target this key proinflammatory cytokine.

Among these, active immunization against TNF with TNF-Kinoid (TNF-K) is a promising development [10], [11]. TNF-K consists of human TNF (hTNF) coupled to a carrier protein, the keyhole limpet haemocyanin (KLH) [12]. This compound is able to break B cell tolerance to hTNF, thereby inducing the production of polyclonal, neutralizing anti-hTNF antibodies and circumventing the concern of anti-drug antibody induction [13]. The proof of concept of TNF-K applicability in RA was performed in hTNF transgenic mouse (TTg) model [14]. We demonstrated the efficacy of TNF-K in TTg, both on clinical arthritis and histological joint inflammation and destruction [13], [15], [16]. The anti-TNF antibody response induced by TNF-K has some characteristics that are fundamental for further developments: TNF-K does not sensitize T cells to native hTNF [13], the anti-hTNF antibody titers are produced as bell-shaped curve along time [15], [16], endogenous TNF does not boost the immune response [16]: only B cell tolerance toward TNF is broken and only TNF-K could boost the immune response.

Based on the proof of concept established in experimental arthritis, TNF-K entered clinical development. A Phase I clinical trial, performed in Crohn’s disease patients, showed it was well tolerated and immunogenic [17]. Here we report the results of a phase IIa pilot study, performed in RA patients, who previously experienced a secondary failure of anti-TNF biologics. We observed a production of anti-TNF antibodies and improvement of some clinical parameters showing the relevance in humans of the anti-TNF therapeutic vaccination concept.

## Methods

### Overall study design

This study was a phase II, randomized, double-blind, multicenter clinical trial examining the safety and immune responses of TNF-K in adults with RA who previously experienced a secondary failure of anti-TNF biologics (ClinicalTrials.gov registry no. NCT01040715). The primary objective was to identify the best dose and schedule of administration of TNF-K in terms of anti-TNF antibody response induced by either 2 injections (day 0 and 28) or three injections (day 0, 7, and 28) of TNF-K at 3 dosages (90, 180, or 360 µg). The study was performed at 21 centers in Argentina, Belgium, Bulgaria, Chile, Croatia, France and Romania, in compliance with European Medicines Agency Guidelines on Clinical Evaluation of New Vaccines, International Committee for Harmonization Guidelines on Good Clinical Practice, and the Declaration of Helsinki (revision of the 52^nd^ World Medical Association General Assembly, Edinburgh, Scotland, October 2000). The study protocol was approved by the medical ethics committees at all participating institutions, as detailed below:

Comite Independiente de Etica para Ensayos en Farmacologia Clinica (Fefym), Buenos Aires; Comité Institucional de Etica en Investigacion en Salud (CIEIS), Sociedad de Beneficencia Hospital Italiano Cordoba city; Comité de Etica del Centro de Investigaciones Reumatologicas, San Miguel de Tucuman; Comité de Docencia e Investigacion - Centro Medico Privado de Reumatologia, San Miguel de Tucuman; Argentina. Université Catholique de Louvain Faculté de Médecine Commission d’Ethique Biomédicale Hospitalo-Facultaire, 1200 Bruxelles, Belgium. Ethics Committee for Multicenter clinical trials, Sofia, Bulgaria. Comité Etico Cientifico Del servicio de Salud Metropolitano Oriente (SSMO) Providencia, Santiago, Chile. Central Ethics Committe, agency for Medecines and Medical devices, Zagreb, Croatia. Comité de Protection des Personnes (CPP) Ile de France II, Paris, France. Ministry of Health, National Ethics Committee for the Clinical study of medecines, Bucharest, Romania. CEIC de Asturias, Hospital Universitario Central de Asturias, Oviedo, Spain. Commission Cantonale d’éthique de la recherche sur l’être humain, Lausanne, Switzerland.

Patients gave written informed consent before inclusion in the trial.

The protocol for this trial and supporting CONSORT checklist are available as supporting information; see S1 Checklist and S1 Protocol.

### Patients

Adults 18 to 70 years with RA were considered for inclusion if they had RA that was diagnosed at least 6 months earlier according to the revised 1987 criteria of the American College of Rheumatology (ACR) and a DAS28-CRP>3.2 at screening [18]. Between March 1, 2010 and August 11, 2011, patients were enrolled if they had an initial response (an ACR20 response, a decrease in DAS28-C-reactive protein [CRP] ≥1.2, or according to the investigator’s opinion) to TNF antagonist (infliximab, adalimumab, etanercept, certolizumab, or golimumab) followed by a treatment failure (an increase in DAS28≥0.6 during the last 6 months, a decrease in EULAR score, or according to the investigator’s opinion). Methotrexate was allowed if administered at a stable dosage ≤20 mg/week for at least 4 weeks. Other non-biological anti-rheumatic drugs had to be discontinued within 4 weeks prior to the first administration of the study product. Patients could not have been previously treated with any RA biological therapy other than TNF antagonists. They were also excluded if they had received intra-articular corticosteroids within 3 months, had a documented severe bacterial infection within 28 days, had a history of or current congestive heart failure, had a known history of active or latent tuberculosis, were positive for human immunodeficiency virus, hepatitis C virus, or hepatitis B virus, or had received any live vaccine within 3 months. The study completion date was July 16, 2012 (month 12).

### Treatments

TNF-K was prepared by aldehyde crosslinking of recombinant human TNF and KLH as described previously [13], [15]. Briefly, KLH and recombinant human TNF were mixed in a 1∶37 molar ratio and crosslinked with 25 mM glutaraldehyde, quenched with glycine, inactivated with 250 mM formaldehyde, and quenched again with glycine. The product was purified by ultrafiltration in phosphate buffer, sterilized by passing through a 0.22-µm filter, formulated with mannitol before lyophilization, and stored at 4°C. Before administration, TNF-K was reconstituted with water for injection (180 µg in 0.3 mL of water) and was emulsified with an equal volume of the adjuvant ISA-51VG (Seppic, Puteaux, France) to make one dose. Patients who received 90 µg TNF-K received one-half of a fully reconstituted dose; patients who received 360 µg TNF-K were administered 2 fully reconstituted doses. The placebo was an equivalent amount of stabilizer (mannitol) in 0.3 mL water and emulsified with an equal volume of the adjuvant ISA-51VG. Both TNF-K and placebo were supplied as white cake in identical numbered vials so that the patient and investigator were blinded to the treatment administered. Doses were injected intramuscularly into the deltoid area of the arm.

**Figure 1:**
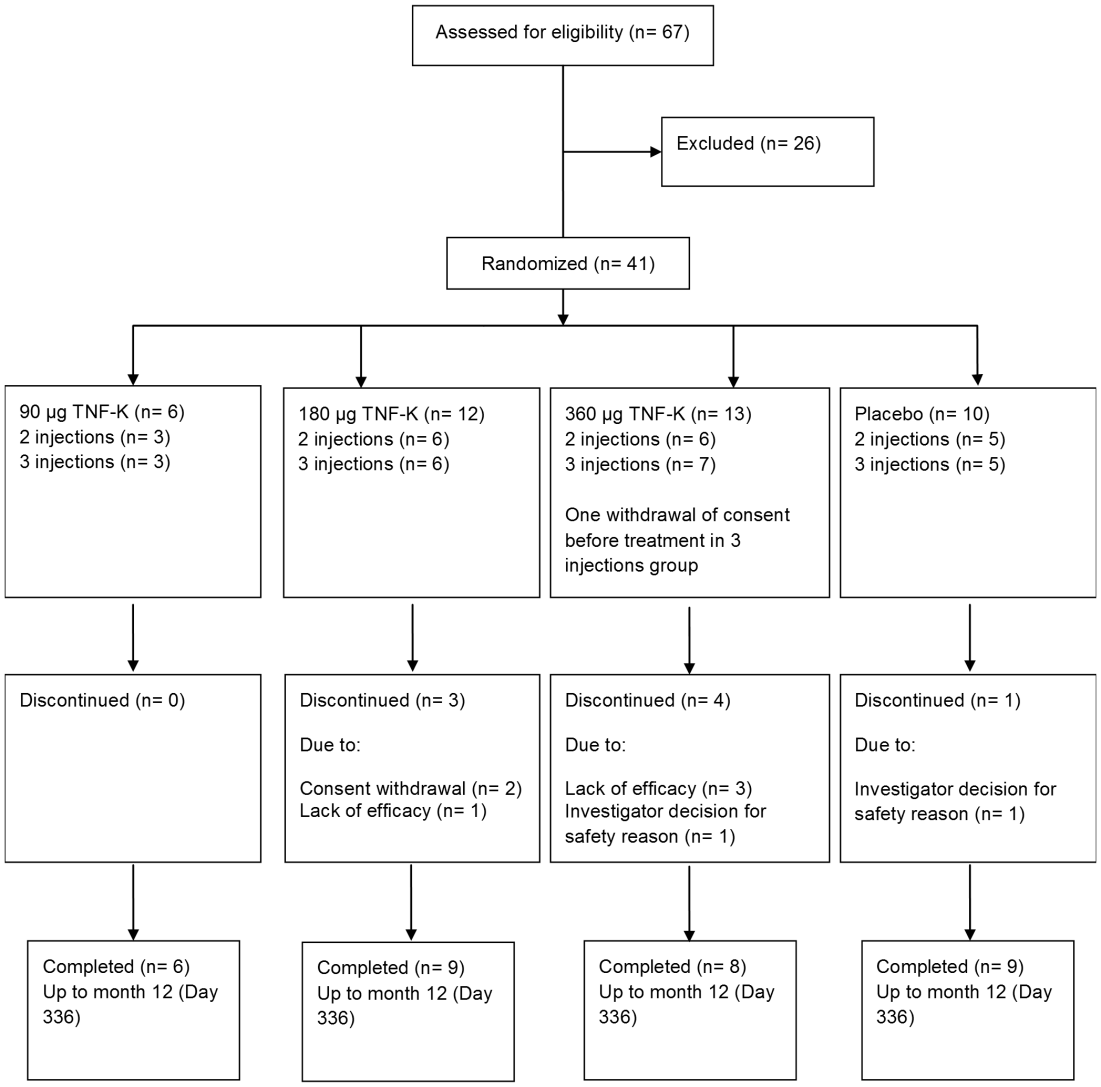
Study design. In the first stage, 8 patients were randomized 3∶1 to receive 90 µg TNF-K or placebo, in the second, 16 patients were randomized 3∶1 to receive 180 µg TNF-K or placebo, and in the third, 17 patients were randomized 3∶1 to receive 360 µg TNF-K or placebo. In each stage, patients were also randomized 1∶1 to receive 2 doses (day 0, 28) or 3 doses (day 0, 7, 28) (arrows). For stages 1 and 2, after 3 patients had been enrolled and no safety issues had been reported for at least 7 days, enrolment in the subsequent stage started in parallel. One patient randomized to receive 3 doses of 360 µg TNF-K withdrew consent prior to treatment. The principal analysis portion of the study continued up to day 84, and the follow-up portion continued up to month 12.

Treatments were randomly assigned using a sequential, incremental, staggered-dose design (Fig. 1). In each stage, patients were also randomized to the study arm and 1∶1 to receive 2 doses (day 0, 28) or 3 doses (day 0, 7, 28). Randomization was via a central interactive web response system, using a randomization list generated with SAS (SAS Institute, Cary, NC). For stages 1 and 2, after 3 patients had been enrolled since at least 7 days and if no safety issue was detected by an independent DSMB (iDSMB) and no related Serious Adverse Event was reported, enrolment in the subsequent stage started in parallel.

### Measurement of anti-TNF antibody titers and Neutralizing Capacity

Anti-TNF antibody titers were measured in sera collected on days 0, 17, 28, 38, 56, 84, 112, 140, 168, 252, and 336 using a direct enzyme-linked immunosorbent assay in which microtiter plates were coated with human recombinant TNF [13]. Serial 2-fold dilutions of serum samples were analyzed. The titer was defined as the highest sample dilution for which the optical density was>3 fold the optical density of a pool of sera from healthy volunteers, which was added in all plates as negative control. The optical density was read at 490 nm. The lower limit of quantification was a titer of 200 (1/dilution). TNF-neutralizing antibodies were measured as described previously [19].

### Lymphoproliferation assay

Peripheral blood mononuclear cells from patients were isolated by Ficoll-hypaque density gradient centrifugation, added (200,000/well) to round-bottom 96-well plates, and incubated at 37°C with medium alone or with TNF-K, recombinant human TNF (Boehringer Ingelheim, Ingelheim am Rhein, Germany), or KLH (Sigma, St Louis, MO, USA) at 0.1, 1 and 10 µg/mL. After 72 h, [^3^H]-thymidine incorporation was assessed as described previously [13]. All samples were tested in quadruplicate. The stimulation index was calculated as the background-corrected counts per minute for stimulated cells divided by the background-corrected counts per minute for cells cultured with medium alone. In this multicenter trial, patients from centers outside of Europe were not analyzed since PBMC isolation could not be performed within 24 hours in the central laboratory located in Europe. Therefore, only 16 patients (10 TNF-K-immunized and 6 placebo) have been analyzed for T-cell response.

### Measurement of anti-drug antibodies

Assessment of ant-drug antibodies was performed with bridge ELISA LISA-TRACKER Premium from Theradiag (Croissy-Beaubourg, France) according to manufacturer’s instructions. Briefly, serial diluted sera from patients were added to previously drug-coated plate, then biotinylated drug was added. After successive washings, streptavidin HRP was added and revealed with the appropriate substrate. Then, optical density was read on 450 nm.

### Assessment of clinical efficacy

DAS28-CRP, swollen and tender joint counts, EULAR response [20], [21], blood CRP concentration, HAQ index, and ACR20, ACR50, and ACR70 response rates [18], [22] were assessed on days 0, 84, 168, and 336.

### Adverse events

Adverse events (AEs) and their occurrence, intensity, and relationship to TNF-K immunization were recorded. AEs, serious adverse events (SAEs), all abnormalities in physical examinations, vital signs, 12-lead electrocardiogram, and clinical laboratory evaluations were recorded up to month 12. Solicited local reactions (pain, tenderness, erythema, swelling, itching, induration, and ulceration) and solicited systemic reactions (fever, vomiting, headache, fatigue, myalgia, and nausea) were recorded by patients on diary cards for 7 days following each dose of TNF-K or placebo.

### Statistical analysis

All analyses were performed for all patients who received at least one dose of TNF-K or placebo. Geometric mean titers (GMT) of anti-TNF and anti-KLH antibodies were calculated on log_10_-transformed titers. For the calculation of GMT, titers below the lower limit of detection (200) were converted to half the lower limit of detection (100). Forty-eight patients (12 per group) were estimated to attain 80% power to detect a difference of 75% between group proportions of patients with at least a 3-fold increase in anti-TNF antibody response vs cut-off at day 38, using a two-sided Fisher’s exact test and a significance level of 0.008 accounting for multiple comparison. In post-hoc analyses, antibody responses were defined as a titer ≥200 at any time point and clinical scores were compared between antibody responders and non-responders (patients with (n = 23) and without (n = 17) detectable anti-TNF antibodies, respectively) using non-parametric Wilcoxon rank-sum tests. A p-value <0.05 was considered to indicate a statistically significant difference for these post-hoc analyses. All calculations were made using SAS version 9.2 (SAS Institute, Cary, NC) or Excel version 14.0.6123.5000 (Microsoft, Redmond, WA).

## Results

### Patients disposition and baseline characteristics

Patients were enrolled between March 1, 2010 and August 11, 2011 (Table 1). As per protocol only 6 patients were included in the 90 µg group in view of the low rate of antibody response. Consequently, forty patients were injected (Fig. 1). One patient randomized to the 360 µg TNF-K dose group did not receive treatment. All patients completed the primary phase to month 3 and thirty-two patients completed the study up to month 12. Of the eight that withdrew between month 3 and 12, 4 were for absence of clinical improvement, 2 following investigator decision for safety reason (flare of RA and persistent RA respectively), 2 withdrew consent.

**Table 1:** Demographics of treated patients at baseline (day 0).

	90 µg TNF-K	180 µg TNF-K	360 µg TNF-K	Placebo	Overall
	N = 6	N = 12	N = 12	N = 10	N = 40
Sex					
Male, n (%)	2 (33.3)	3 (25.0)	1 (8.3)	1 (10.0)	7 (17.5)
Female, n (%)	4 (66.7)	9 (75.0)	11 (91.7)	9 (90.0)	33 (82.5)
Age (years)					
Mean ± SD	55.5±13.0	52.9±12.5	46.6±13.0	56.0±10.0	52.2±12.3
Range	31–69	32–69	20–65	34–68	20–69
Disease duration (years)					
Mean ± SD	16.4±11.5	8.6±5.4	13.3±7.8	17.1±16.6	13.2±10.8
Range	6–30	2–19	5–25	2–60	2–60
Tender joint count					
Mean ± SD	13.0±8.3	16.9±8.9	13.25±6.3	8.8±5.3	13.2±7.6
Range	4–26	0–28	5–26	1–15	0–28
Swollen joint count					
Mean ± SD	10.2±8.0	9.5±5.8	9.7±3.2	7.6±3.3	9.2±4.9
Range	3–24	0–17	3–14	1–12	0–24
DAS28					
Mean ± SD	4.7±1.1	4.8±1.2	4.6±0.9	4.3±0.8	4.6±1.0
Range	3–6	1.7–6.5	3.1–6.3	2.8–5.4	1.7–6.5
Previous anti-TNF					
Infliximab	4	5	3	3	15
Adalimumab	3	5	0	4	12
Etanercept	1	3	8	5	17
Certolizumab	0	0	1	1	2
Anti-drug antibodies Positive, n (%)	4 (66.7)	5 (41.7)	1 (8.3)	4 (40.0)	14 (35.0)
Rheumatoid factor ≥40 IU/mL, n (%)	4 (66.7)	5 (41.7)	8 (66.7)	5 (50.0)	22 (55.0)

Abbreviations: SD, standard deviation.

Baseline disease characteristics were similar in the four treatment groups. Of the 40 treated patients, 15 had received infliximab (12 in the TNF-K groups and 3 in the placebo group), 12 had received adalimumab (8 in the TNF-K groups and 4 in the placebo group), 2 had received certolizumab (1 in the TNF-K groups and 1 in the placebo group), and 17 had received etanercept (12 in the TNF-K groups and 5 in the placebo group). Six patients (3 in the TNF-K groups and 3 in the placebo group) had previously received treatment with at least 2 monoclonal antibodies. Fourteen (35.0%) of the 40 patients were anti-drug antibody-positive at screening: 8 had antibodies to infliximab, 3 had antibodies to adalimumab, and 3 had antibodies to both infliximab and adalimumab.

### Immunogenicity

**Figure 2:**
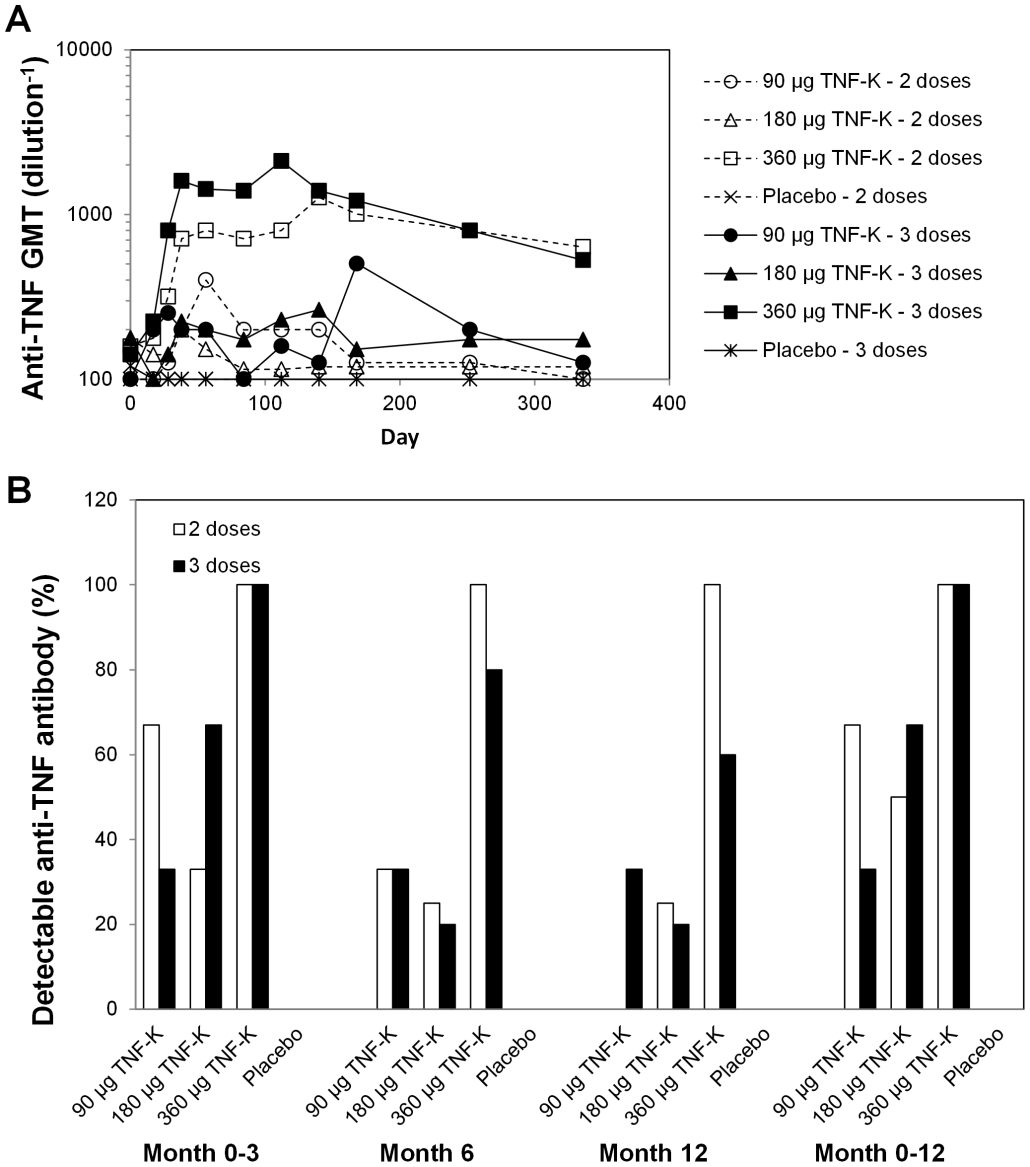
Humoral immune response to TNF. Patients were treated with 2 doses (days 0 and 28) or 3 doses (days 0, 7, and 28) of placebo or 90, 180, or 360 µg TNF-K. Anti-TNF antibody titers were determined by enzyme-linked immunosorbent assay. (A) GMTs. (B) Percent of patients in each treatment group with detectable anti-TNF antibodies (titer ≥200) up to month 3 (at study day 38, 56, or 84), at month 6, at month 12, or at any time up to month 12 (i.e., antibody responders).

Immunization with TNF-K induced a dose- and schedule-dependent increase in anti-TNF antibody levels (Fig. 2A), although a significant difference between TNF-K groups was not found for the primary endpoint (S1 Table). GMTs were numerically higher in patients receiving 360 µg TNF-K than in patients receiving 90 or 180 µg. All 12 patients immunized with 360 µg TNF-K had an antibody response (i.e., detectable anti-TNF antibody at least at 1 time point), whereas no more than two-thirds of the patients receiving 90 or 180 µg TNF-K were antibody responders (Fig. 2B). In patients receiving 360 µg TNF-K, GMTs were highest on day 112 (3 doses) or 140 (2 doses), after which titers decreased gradually. At this dose of TNF-K, GMTs were higher in patients injected with 3 doses (on days 0, 7, and 28) than in patients injected with 2 doses (on days 0 and 28).

Most antibodies were of IgG1 and IgG3 isotypes and no IgE antibodies were detected (S1 Figure). Anti-TNF antibodies were not detected in the placebo group (S1 Table). TNF-neutralizing activity, measured in sera collected on day 56, was not detectable for any patient at the 1/100 dilution on L929 cells (S2 Table).

**Figure 3:**
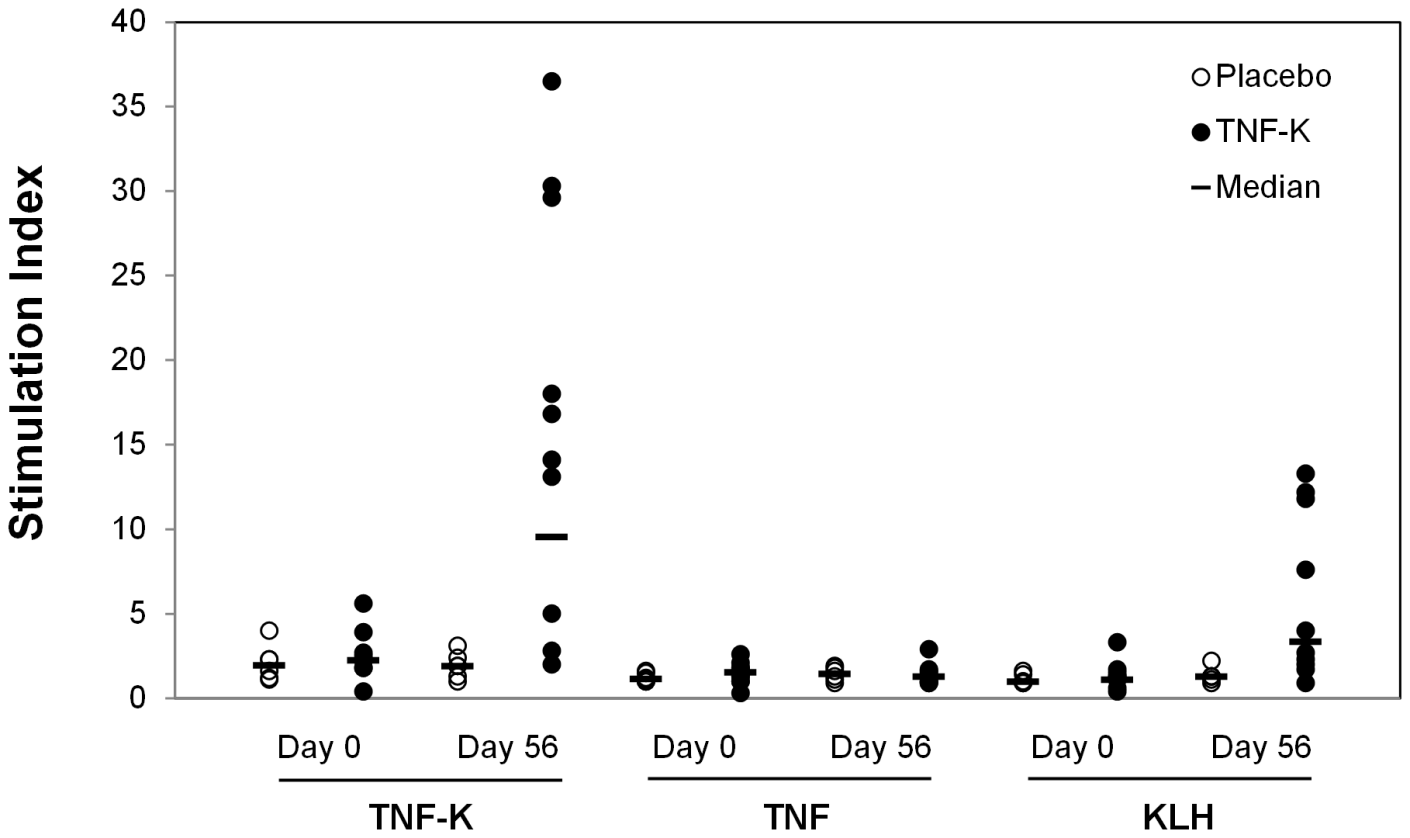
T cell response to TNF. Peripheral blood mononuclear cells were collected on days 0 and 56 and treated in vitro with medium (control) or with 10 µg/mL TNF-K, TNF, or KLH. Lymphoproliferation was assessed after 72 h by ^3^H-thymidine incorporation. Shown is the stimulation index (fold-increase in lymphoproliferation vs. control) for cells from patients treated with placebo (n = 6) or TNF-K (n = 10).

T-cell immune responses were assessed by measuring lymphoproliferation in vitro. As shown on Fig. 3, high stimulation indexes were obtained with TNF-K and KLH; importantly, no T-cell response was observed with TNF (S3 Table).

### Adverse events

Rates of solicited reactions (injection-site and systemic reactions) were comparable in TNF-K-treated and placebo-treated patients (Table 2). The most common local reactions were injection-site pain and tenderness, and myalgia was the most common systemic reaction, followed by fatigue and headache. Most reactions were of mild to moderate intensity. Severe reactions were not more common in patients treated with TNF-K than in patients who received placebo.

**Table 2:** Adverse reactions solicited post injection.

		TNF-K	Placebo
		N(%)	N(%)
Reaction	Severity	N = 28	N = 10
Injection site	Any	19 (67.9)	7 (70.0)
	Grade 3	9 (32.1)	3 (30.0)
Pain^a^	Any	19 (67.9)	5 (50.0)
	Grade 3	9 (32.1)	3 (30.0)
Tenderness^b^	Any	10 (35.7)	5 (50.0)
	Grade 3	6 (21.4)	1 (10.0)
Itching^c^	Any	6 (21.4)	1 (10.0)
	Grade 3	1 (3.6)	0 (0.0)
Swelling^d^	Any	2 (7.1)	0 (0.0)
	Grade 3	0 (0.0)	0 (0.0)
Redness^d^	Any	1 (3.6)	1 (10.0)
	Grade 3	0 (0.0)	0 (0.0)
Induration^d^	Any	1 (3.6)	1 (10.0)
	Grade 3	0 (0.0)	0 (0.0)
Ulceration^e^	Any	0 (0.0)	0 (0.0)
	Grade 3	0 (0.0)	0 (0.0)
Systemic	Any	23 (82.1)	8 (80.0)
	Grade 3	5 (17.9)	4 (40.0)
Fever^f^	Any	5 (17.9)	1 (10.0)
	Grade 3	0 (0.0)	0 (0.0)
Vomiting^g^	Any	1 (3.6)	0 (0.0)
	Grade 3	0 (0.0)	0 (0.0)
Headache^a^	Any	13 (46.4)	4 (40.0)
	Grade 3	1 (3.6)	0 (0.0)
Fatigue^a^	Any	16 (57.1)	6 (60.0)
	Grade 3	3 (10.7)	1 (10.0)
Myalgia^a^	Any	18 (64.3)	6 (60.0)
	Grade 3	3 (10.7)	4 (40.0)
Nausea^a^	Any	6 (21.4)	4 (40.0)
	Grade 3	0 (0.0)	0 (0.0)

Information on solicited symptoms was not obtained for 2 patients in the TNF-K.

a Grade 1, did not interfere with daily activities; grade 2, caused some interference with daily activities; grade 3 significantly affected or prevented daily activities.

b Grade 1, mild pain to touch; grade 2, pain with movement; grade 3, significant pain at rest.

c Grade 1, localized; grade 2, intense or widespread; grade 3, prevented daily activity.

d Grade 1, largest diameter>0 to <30 mm; grade 2, largest diameter 30 to <120 mm; grade 3, largest diameter ≥120 mm.

e Not assigned a grade.

f Grade 1, 37.5°C to <38°C; grade 2, 38°C to <39°C; grade 3, ≥39°C.

g Grade 1, one to 2 episodes/24 h; grade 2, more than 2 episodes/24 h; grade 3, intravenous hydration was required.

AEs were reported by most of the patients treated with TNF-K (23/30 [76.7%]) or placebo (9/10 [90.0%]) and infections were reported by similar proportions of patients treated with TNF-K and placebo (43.3% vs. 50.0%). Treatment-related AEs were reported by 14 patients treated with TNF-K (46.7%) and 6 treated with placebo (60.0%). One non-serious AE considered possibly treatment-related (moderate polyarthritis 57 days after a second dose of 180 µg TNF-K) led to early discontinuation of the treatment and one patient treated with TNF-K reported a severe AE (headache) considered by the investigator to be treatment-related.

SAEs were reported by 4 patients treated with TNF-K (13.3% - urinary tract infection, leiomyoma, thyroid multifocal carcinoma, synovitis) and 3 treated with placebo (30.0% - humerus fracture and device breakage, bunion, bronchitis). None of the SAEs was considered treatment-related. No case of serious infection, no tuberculosis, no malignancies, no anaphylactic reactions, no serum sickness-like reactions and no deaths related to treatment were reported up to month 12.

### Clinical efficacy

Clinical response rates between TNF-K and placebo groups were not significantly different, but non-significant trends towards better clinical responses were seen in patients administered 360 µg TNF-K who had the most pronounced antibody responses. In post-hoc analysis, clinical responses were therefore compared between antibody responders (n = 23) and non-responders (n = 17).

**Figure 4:**
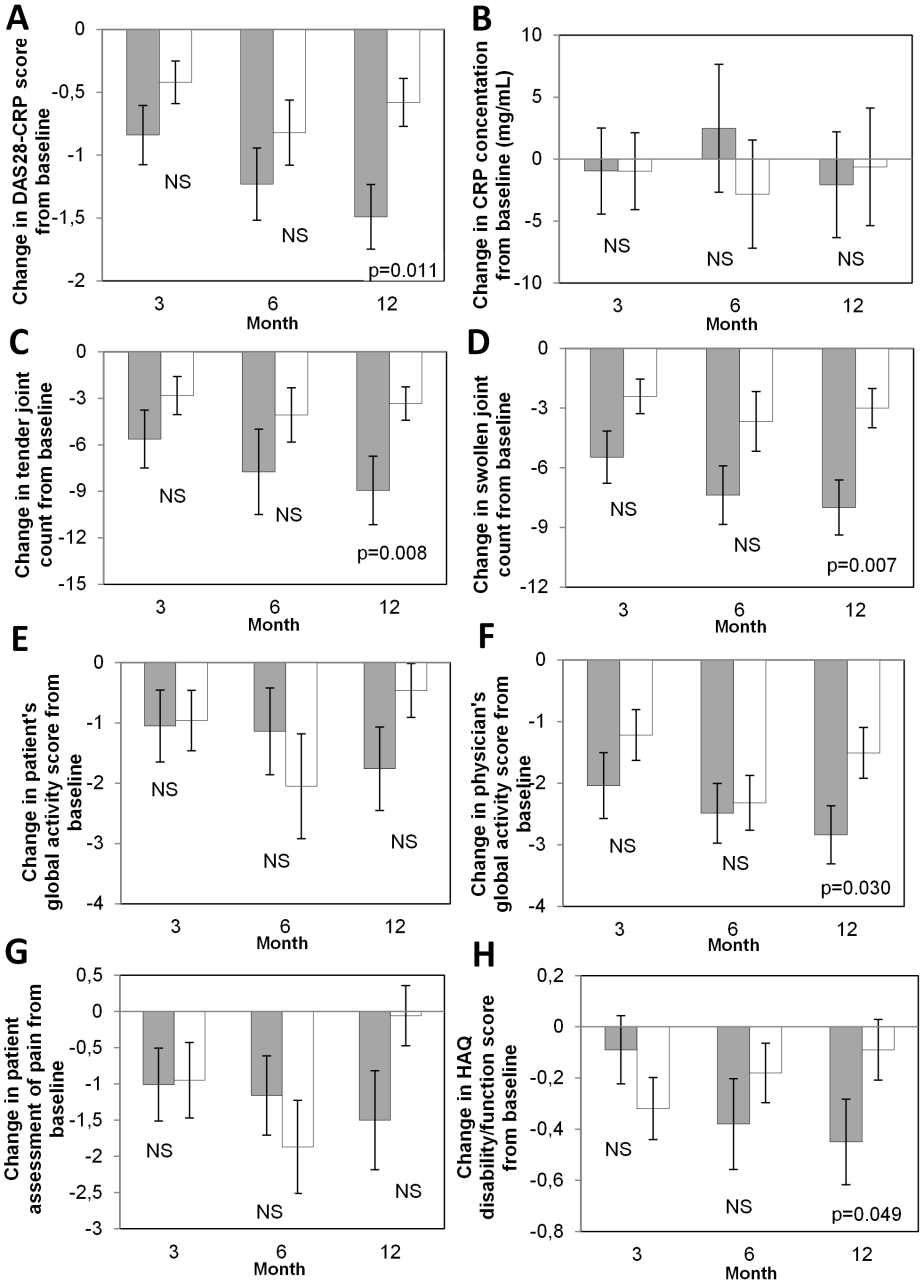
Difference from baseline for clinical assessments in anti-TNF antibody responders and non-responders. Post-hoc analyses: Mean changes in clinical assessments from baseline are shown at months 3, 6, and 12 for antibody responders (detectable anti-TNF antibodies at any time; gray bars) and for non-responders (white bars). (A) DAS28-CRP. (B) CRP. (C) Tender joints count. (D) Swollen joints count. (E) Patient’s global activity score. (F) Physician’s global activity score. (G) Patient assessment of pain. (H) Change in HAQ disability/function score. Error bars indicate standard error of the mean. P-values were determined by Wilcoxon rank-sum test. NS, not significant (p≥0.05). In responders, n = 19 at month 3, 16 at months 6 and 12 except at month 3 for Physician GAS n = 18 and for CRP n = 20. In non-responders, n = 17 at month 3 and 15 at months 6 and 12 except at month 6 n = 14 for CRP and DAS 28 score.

DAS28-CRP, mean tender joint count, swollen joint count, patient’s global assessment, physician’s global assessment, patient assessment of pain, and HAQ score improved progressively up to month 12 in antibody responders, whereas no clear trend over time was observed in patients who remained negative for anti-TNF antibodies (Fig. 4). At month 12, antibody responders had significantly greater decreases from baseline than non-responders for DAS28-CRP (p = 0.011 using Wilcoxon rank-sum test), tender joint count (p = 0.008), swollen joint count (p = 0.007), physician global activity score (p = 0.030), and HAQ disability/function score (p = 0.049).

## Discussion

This pilot study showed that TNF-K was able to induce anti-TNF antibodies in patients with RA who had experienced a secondary failure with anti-TNF biologics. This antibody response was dose-dependent, since only patients immunized with 360 µg TNF-K had all an antibody response. Thus active anti-cytokine immunization with Kinoid appeared to be feasible in humans against TNF as shown here and also against IFNα as previously reported in lupus patients [23]. Previous studies with two other agents combining TNF and a foreign carrier (CYT007-TNFQb and AutoVac TNF106) induced high titers of neutralizing antibodies in mice but were poorly immunogenic in humans [24]–[26]. We did not detect neutralizing activity in sera collected at day 56 using the classical test using L929 cells, at 1/100 dilution, despite high anti-TNF antibodies titers and some clinical effect. This may be explained by too early assessment of neutralizing antibodies or by the fact that binding antibodies by themselves may enhance TNF clearance through immune complexes formation. We also showed that the cellular response was strong when PBMCs from TNF-K immunized patients were stimulated with TNF-K or the carrier protein (KLH). In contrast TNF-K did not induce a T cell response to native TNF, meaning a lack of induction of auto-reactive T-cells specific of TNF. This result was expected since it was also observed in our previous studies on TNF transgenic mice [13], [15]–[16]. The lack of T cell response against native cytokine was also observed with the IFN-K despite a strong humoral response in lupus patients [23]. This T cell response limited to the carrier protein or the Kinoid confirms that, like classical paediatric vaccines, the carrier protein provides T cell help for a polyclonal B cell response to the conjugated cytokine [27].

This study also showed that TNF-K was well tolerated. The injection of TNF-K did not induce any severe local reactions and no unexpected safety concerns were identified. No cases of severe infection including tuberculosis were observed. Larger studies should be performed to evaluate the absolute risk of severe infection, including tuberculosis, and compare with other strategies of TNF blockade [28].

Despite the small size of the study and the fact it was not specifically powered to detect clinical improvements, we observed progressive and significant improvements in the group of antibody responders compared to the non-responders; this was noticed for DAS28-CRP, tender and swollen joint counts, physician global activity score, and HAQ disability/function score, suggesting a clinical effect of the polyclonal anti-TNF antibodies induced by TNF-K. A similar observation was made in mice models with an association of the immune responder status to TNF-K with the clinical response [29].

A potential bias in our study is the absence of stratification with regards to previous anti-TNF treatment. Indeed, Van Vollenhoven et al recently reported that etanercept as second anti-TNF following loss of efficacy of a first anti-TNF (adalimumab or infliximab) yielded a better clinical response than adalimumab or infliximab following etanercept [30]. This effect is not related to immunogenicity, i.e. to the development of specific antidrug antibodies to one anti-TNF [31]. In our trial, 8 out of 12 patients in the 360 µg group had a secondary failure to etanercept, an unbalance that might have introduced a negative bias in our study. However the best clinical response was observed in patients with highest antibody titers and was apparently not affected, although the number of patients does not allow drawing definite conclusions. One hypothesis might be that the polyclonal antibody response induced by therapeutic vaccination circumvents this limitation of monoclonal antibodies. Further trials are needed to address this question.

In conclusion, this pilot study showed that anti-TNF therapeutic vaccination with TNF-K was feasible, safe and promising in RA. Further studies are warranted to examine higher and repeated doses to establish the best conditions for clinical improvement.

## Supporting Information

S1 Figure **Geometric mean fold seroconversion by isotype.** Anti-TNF antibody was detected by enzyme-linked immunosorbent assay as previously described (Le Buanec et al. Proc Natl Acad Sci USA. 2006; 103(51):19442-7) but with isotype-specific secondary antibodies. The seroconversion ratio was calculated as the ratio of the extrapolated optical density for the highest dilution of tested serum divided by the mean optical density for a pool of serum from 3 healthy donors.
(PDF)

S1 Table **Anti-TNF antibody Titers.** Individual anti-TNF antibody titer (Dil-1)
(PDF)

S2 Table **Neutralizing Capacity.** Individual neutralization capacity (NC50 Dil-1) at D0, D17 and D56
(PDF)

S3 Table **Lymphoproliferation assay.** Stimulation index of patient PBMCs stimulated with TNF-Kinoid, TNF and KLH at 0.1, 1 and 10 µg/ml.
(PDF)

S1 Protocol **Protocol of the study.** A phase II, randomized, double-blind, controlled study to evaluate the immune responses, safety and clinical efficacy of three doses of TNF-Kinoid in adult patients with rheumatoid arthritis who have relapsed despite anti-TNF biological therapy.
(PDF)

S1 Checklist **CONSORT 2010 checklist of the present study.**
(DOC)
