# Supplementary material for: Therapeutic Vaccination with TNF-Kinoid in TNF Antagonist-Resistant Rheumatoid Arthritis: A Phase II Randomized, Controlled Clinical Trial
Source: PLoS One. 2014 Dec 17;9(12):e113465. doi: 10.1371/journal.pone.0113465 (PMC4269456; doi:10.1371/journal.pone.0113465)
Supplement: S1 Figure — Geometric mean fold seroconversion by isotype. Anti-TNF antibody was detected by enzyme-linked immunosorbent assay as previously described (Le Buanec et al. Proc Natl Acad Sci USA. 2006; 103(51):19442-7) but with isotype-specific secondary antibodies. The seroconversion ratio was calculated as the ratio of the extrapolated optical density for the highest dilution of tested serum divided by the mean optical density for a pool of serum from 3 healthy donors. (PDF) [file pone.0113465.s001.pdf]

Supplemental Figure 1. Geometric mean fold seroconversion by isotype

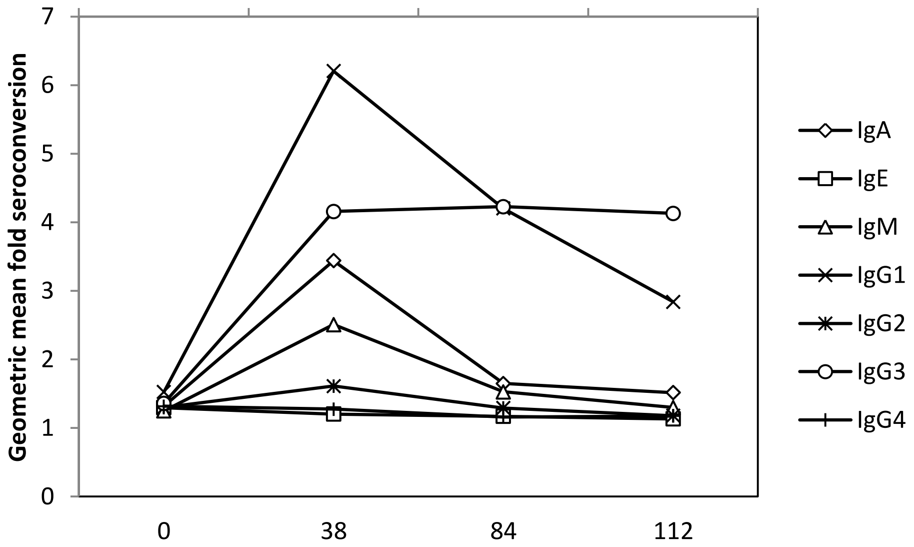

Anti-TNF antibody was detected by enzyme-linked immunosorbent assay as previously described (Le Buanec et al. Proc Natl Acad Sci USA. 2006;103(51):19442-7) but with isotype-specific secondary antibodies. The seroconversion ratio was calculated as the ratio of the extrapolated optical density for the highest dilution of tested serum divided by the mean optical density for a pool of serum from 3 healthy donors.
