# Supplementary material for: Therapeutic Vaccination with TNF-Kinoid in TNF Antagonist-Resistant Rheumatoid Arthritis: A Phase II Randomized, Controlled Clinical Trial
Source: PLoS One. 2014 Dec 17;9(12):e113465. doi: 10.1371/journal.pone.0113465 (PMC4269456; doi:10.1371/journal.pone.0113465)
Supplement: S1 Table — Anti-TNF antibody Titers. Individual anti-TNF antibody titer (Dil-1) (PDF) [file pone.0113465.s002.pdf]

Durez P et al; Therapeutic vaccination with TNF-Kinoid in TNF antagonist-resistant rheumatoid arthritis: a phase II randomized, controlled clinical trial.

Table S1. Anti-TNF antibody Titers. Individual anti-TNF antibody titers (Dil-1)

(light grey)	Kinoid group
(dark grey)	Placebo group

90µg D0-28	Visit	Day	10204	20101	80302	10202
	V2	0	100	100	100	100
	V4	17	100	100	100	100
	V5	28	200	100	100	100
	V6	38	800	100	100	100
	V7	56	3200	100	200	100
	V8	84	800	100	100	100
	V9	112	800	100	100	
	V10	140	400	100	100	
	V11	168	200	100	100	
	V12	252	200	100	100	
	V13	336	100	100	100	

90µg D0-7-28	Visit	Day	10205	80102	80103	10208
	V2	0	100	100	100	100
	V4	17	100	800	100	100
	V5	28	100	1600	100	100
	V6	38	100	800	100	100
	V7	56	100	800	100	100
	V8	84	100	no sample	100	100
	V9	112	100	400	100	
	V10	140	100	200	100	
	V11	168	100	12800	100	
	V12	252	100	800	100	
	V13	336	100	200	100	

	Visit	Day	10207	10301	30301	60103	60201	70402	10206	20501
180µg D0-28	V2	0	3200	100	100	100	100	100	100	100
	V4	17	800	100	100	100	100	100	100	100
	V5	28	400	100	100	100	100	200	100	100
	V6	38	400	100	400	100	100	400	100	100
	V7	56	200	100	400	100	100		100	100
	V8	84	100	100	200	100	100		100	100
	V9	112	200	100	100	100	100			
	V10	140	200	100	100		100			
	V11	168	200	100	100		100			
	V12	252	200	100	100		100			
	V13	336	200	100	100		100			

180µg D0-7-28	Visit	Day	10302	60501	70103	80101	80104	80106	60102*	80105
	V2	0	100	100	800	100	400	100	100	200
	V4	17	100	100	100	100	100	100	100	100
	V5	28	100	100	400	100	200	100	100	100
	V6	38	400	100	800	100	400	100	100	100
	V7	56	200	100	800	200	200	100	100	100
	V8	84		100	400	200	200	100	100	100
	V9	112		100	400	100	1600	100	200	
	V10	140		100	800	100	1600	100	200	
	V11	168		100	100	100	800	100	200	
	V12	252		100	100	100	1600	100	200	
	V13	336		100	100	100	1600	100	200	

	Visit	Day	10209	30303	50201	50304	50602	70104	21102	30701
360µg D0-28	V2	0	100	100	100	1600	100	100	100	100
	V4	17	100	100	100	800	400	100	100	100
	V5	28	400	100	400	800	800	100	100	100
	V6	38	800	400	800	800	1600	400	100	100
	V7	56	400	400	3200	1600	1600	200	100	100
	V8	84	200	200	6400	3200	1600	100	100	100
	V9	112	200	400	12800	1600	1600	100		
	V10	140		400	6400	800				
	V11	168		400	3200	800				
	V12	252		400	3200	400				
	V13	336		400	3200	200				

360µg D0-7-28	Visit	Day	30302	50301	50601	80109	80111	80305	50303	50603
	V2	0	100	100	100	100	100	800	100	100
	V4	17	100	400	1600	100	100	200	100	100
	V5	28	100	6400	6400	800	100	800	100	100
	V6	38	1600	6400	12800	800	100	no sample	100	100
	V7	56	1600	3200	6400	1600	200	800	100	100
	V8	84	800	1600	6400	3200	200		100	100
	V9	112	800	1600	12800	12800	200			
	V10	140	800	1600	6400	6400	100			
	V11	168	800	800	6400	6400	100			
	V12	252	400	400	3200	6400	100			
	V13	336	400	100	3200	3200	100			

60102 * : Patient injected with kinoid at V4 and V5 whereas randomized in Placebo group
