# Supplementary material for: Therapeutic Vaccination with TNF-Kinoid in TNF Antagonist-Resistant Rheumatoid Arthritis: A Phase II Randomized, Controlled Clinical Trial
Source: PLoS One. 2014 Dec 17;9(12):e113465. doi: 10.1371/journal.pone.0113465 (PMC4269456; doi:10.1371/journal.pone.0113465)
Supplement: S2 Table — Neutralizing Capacity. Individual neutralization capacity (NC50 Dil-1) at D0, D17 and D56 (PDF) [file pone.0113465.s003.pdf]

Durez P et al; Therapeutic vaccination with TNF-Kinoid in TNF antagonist-resistant rheumatoid arthritis: a phase II randomized, controlled clinical trial.

Table S2: Results summary of Neutralization Capacity

Patient #	Trial group	NC50 results (dil-1)		
		D0/V2*	D17/V4*	D56/V7*
#010204	TNF-K 90µg	<100	-	<100
#080102	TNF-K 90µg	<100	-	<100
#080302	TNF-K 90µg	<100	-	<100
#010302	TNF-K 180µg	<100	-	<100
#030301	TNF-K 180µg	<100	-	<100
#060102	TNF-K 180µg	<100	-	<100
#060103	TNF-K 180µg	<100	-	<100
#060501	TNF-K 180µg	<100	-	<100
#070103	TNF-K 180µg	438.54	<100	<100
#080101	TNF-K 180µg	<100	-	<100
#080104	TNF-K 180µg	<100	<100	<100
#080106	TNF-K 180µg	<100	-	<100
#010209	TNF-K 360µg	<100	-	<100
#030302	TNF-K 360µg	<100	-	<100
#030303	TNF-K 360µg	<100	-	<100
#050201	TNF-K 360µg	<100	-	<100
#050301	TNF-K 360µg	<100	-	<100
#050601	TNF-K 360µg	<100	-	<100
#050602	TNF-K 360µg	<100	-	<100
#070104	TNF-K 360µg	<100	-	<100
#080109	TNF-K 360µg	<100	-	<100
#080305	TNF-K 360µg	<100	<100	<100

* Day of sampling / Visit number - : Not tested
