# Supplementary material for: Therapeutic Vaccination with TNF-Kinoid in TNF Antagonist-Resistant Rheumatoid Arthritis: A Phase II Randomized, Controlled Clinical Trial
Source: PLoS One. 2014 Dec 17;9(12):e113465. doi: 10.1371/journal.pone.0113465 (PMC4269456; doi:10.1371/journal.pone.0113465)
Supplement: S3 Table — Lymphoproliferation assay. Stimulation index of patient PBMCs stimulated with TNF-Kinoid, TNF and KLH at 0.1, 1 and 10 µg/ml. (PDF) [file pone.0113465.s004.pdf]

Durez P et al; Therapeutic vaccination with TNF-Kinoid in TNF antagonist-resistant rheumatoid arthritis: a phase II randomized, controlled clinical trial.

Table S3. Lymphoproliferation assay. Stimulation index of patient PBMCs stimulated with TNF-Kinoid, TNF and KLH at 0.1, 1 and 10 µg/ml.

Subject	Period*	Stimuli								
		Kinoid (µg/ml)			TNF (µg/ml)			KLH (µg/ml)		
		0.1	1	10	0.1	1	10	0.1	1	10
**01-02-02**	D0/V2	1,6	2,1	4,0	2,5	2,2	1,6	2,0	1,7	1,4
**01-02-02**	D56/V7	1,5	2,0	3,1	2,0	1,8	1,3	1,4	1,8	1,3
**01-02-04**	D0/V2	1,4	2,4	2,3	2,0	2,3	1,8	1,7	1,4	1,1
**01-02-04**	D56/V7	7,2	13,2	14,1	2,6	2,7	2,9	3,2	4,9	13,3
**01-02-05**	D0/V2	1,0	0,4	0,4	0,4	0,3	0,3	0,4	0,4	0,4
**01-02-05**	D56/V7	1,3	1,9	2,0	1,2	1,0	0,9	0,6	1,3	0,9
**01-02-06**	D0/V2	1,0	1,0	1,1	1,2	1,1	1,2	1,2	1,0	0,9
**01-02-06**	D56/V7	1,1	0,9	1,0	1,1	0,8	0,9	1,0	1,1	1,1
**01-02-07**	D0/V2	1,1	1,5	1,8	1,3	1,1	1,3	1,0	1,3	1,1
**01-02-07**	D56/V7	3,0	9,1	16,8	1,0	1,0	0,9	0,9	1,7	4,0
**01-02-08**	D0/V2	1,2	1,2	1,6	1,1	1,1	1,1	1,0	0,9	1,0
**01-02-08**	D56/V7	1,0	1,3	1,9	1,3	1,3	1,1	0,9	1,1	1,3
**01-02-09**	D0/V2	0,6	1,0	1,8	1,2	1,3	1,7	1,1	1,1	1,2
**01-02-09**	D56/V7	1,4	4,0	13,1	1,1	1,4	1,3	1,1	1,5	2,0
**01-03-01**	D0/V2	1,9	2,8	2,2	1,0	1,0	2,6	1,1	0,9	0,6
**01-03-01**	D56/V7	1,5	2,5	2,8	1,3	1,6	1,7	1,9	1,2	2,3
**01-03-02**	D0/V2	1,2	2,1	3,9	2,0	1,5	1,3	1,6	1,4	1,3
**01-03-02**	D56/V7	9,2	21,7	30,3	2,1	1,6	1,6	1,6	2,6	7,6
**02-01-01**	D0/V2	1,4	1,5	2,7	2,9	2,0	1,8	2,0	2,5	3,3
**02-01-01**	D56/V7	1,7	2,7	5,0	1,3	0,9	1,0	1,4	2,0	2,7
**02-05-01**	D0/V2	0,9	1,0	2,3	1,1	1,1	1,5	1,6	1,3	1,0
**02-05-01**	D56/V7	0,8	1,3	2,4	1,8	1,5	1,9	1,7	1,7	2,2
**02-11-02**	D0/V2	1,0	0,6	1,2	1,2	1,0	1,0	1,3	0,5	1,6
**02-11-02**	D56/V7	0,7	1,3	1,9	1,8	1,2	1,8	1,3	2,0	1,3
**03-03-01**	D0/V2	1,6	2,8	5,6	3,9	4,4	2,1	1,8	2,4	1,5
**03-03-01**	D56/V7	6,7	22,3	36,5	2,3	2,2	1,0	1,8	2,9	12,2
**03-03-02**	D0/V2	1,0	1,2	2,3	1,5	1,0	1,0	1,3	1,4	1,3
**03-03-02**	D56/V7	7,5	21,8	29,6	1,8	1,4	1,2	1,8	4,8	11,8
**03-03-03**	D0/V2	1,1	1,7	2,5	1,4	1,1	1,0	1,1	1,1	1,7
**03-03-03**	D56/V7	3,4	7,9	18,0	2,1	2,1	1,5	2,4	2,1	2,6
**03-07-01**	D0/V2	0,8	1,4	2,3	0,9	0,9	1,0	0,9	1,2	0,9
**03-07-01**	D56/V7	1,0	0,7	1,3	1,3	1,6	1,6	1,3	1,5	0,9

*Day of sampling/Visit number. In gray, placebo patients.
