# Supplementary material for: Therapeutic Vaccination with TNF-Kinoid in TNF Antagonist-Resistant Rheumatoid Arthritis: A Phase II Randomized, Controlled Clinical Trial
Source: PLoS One. 2014 Dec 17;9(12):e113465. doi: 10.1371/journal.pone.0113465 (PMC4269456; doi:10.1371/journal.pone.0113465)
Supplement: S1 Protocol — Protocol of the study. A phase II, randomized, double-blind, controlled study to evaluate the immune responses, safety and clinical efficacy of three doses of TNF-Kinoid in adult patients with rheumatoid arthritis who have relapsed despite anti-TNF biological therapy. (PDF) [file pone.0113465.s005.pdf]

Sponsor:
Neovacs SA
3-5 Impasse Reille
75014 Paris
France

**Study products**	Neovacs' TNF-Kinoid 90 mcg combined with ISA-51 adjuvant<br>Neovacs' TNF-Kinoid 180 mcg combined with ISA-51 adjuvant<br>Neovacs' TNF-Kinoid 360 mcg combined with ISA-51 adjuvant
**Study number(s) and abbreviated title(s)**	TNF-K-003
**EudraCT number**	2009-012041-35
**Date of original protocol**	09 September 2009 - Final
**Amendment 01**	03 May 2010 – Final
**Amendment 02**	29 November 2010 – Final
**Title**	A phase II, randomized, double-blind, controlled study to evaluate the immune responses, safety and clinical efficacy of three doses of Neovacs' TNF-Kinoid in adult patients with rheumatoid arthritis who have relapsed despite anti-TNFα biological therapy.
**Coordinating author**	Pierre Vandepapelière**,** MD, PhD<br>*(Chief Medical Officer, Neovacs)*
**Contributing authors**	Sarah Créac'h, *Clinical coordinator, Neovacs*<br>Linda Gibbs, *Medical Writer, 4Clinics*<br><br>Philippe Wiesel*, MD Senior Clinical Development Advisor, Genexion*

Sponsor Approval

**Study number(s) and abbreviated title(s)**	TNF-K-003
**EudraCT number**	2009-012041-35
**Amendment number**	Amendment 02
**Amendment 01 date**	03 May 2010
**Amendment 02 date**	29 November 2010
**Title**	A phase II, randomized, double-blind, controlled study to evaluate the immune responses, safety and clinical efficacy of three doses of Neovacs' TNF-Kinoid in adult patients with rheumatoid arthritis who have relapsed despite anti-TNFα biological therapy.
**Sponsor signatory approval**	Date and signature

Pierre Vandepapelière
(Chief Medical Officer)

30 NOV 2010.

Guy-Charles Fanneau de la Horie
(Chief Executive Officer)

30 Nov. 2010

Investigator Agreement

**Study number(s) and abbreviated title(s)**	TNF-K-003
**EudraCT number**	2009-012041-35
**Amendment number**	Amendment 02
**Amendment 01 date**	03 May 2010
**Amendment 02 date**	29 November 2010
**Protocol title**	A phase II, randomized, double-blind, controlled study to evaluate the immune responses, safety and clinical efficacy of three doses of Neovacs' TNF-Kinoid in adult patients with rheumatoid arthritis who have relapsed despite anti-TNFα biological therapy

I agree:

- To assume responsibility for the proper conduct of the study at this site.
- To conduct the study in compliance with this protocol, any mutually agreed future protocol amendments, and with any other study conduct procedures provided by Neovacs SA.
- To ensure that all persons assisting me with the study are adequately informed about the Neovacs SA investigational product(s) and other study-related duties and functions as described in the protocol.
- Not to implement any changes to the protocol without agreement from Neovacs SA and prior review and written approval from the Institutional Review Board (IRB) or Independent Ethics Committee (IEC), except where necessary to eliminate an immediate hazard to the patients, or where permitted by all applicable regulatory requirements (for example, for administrative aspects of the study).
- That I am thoroughly familiar with the appropriate use of the study product(s), as described in this protocol, and any other information provided by Neovacs SA, including, but not limited to, the following: the current Investigator's Brochure (IB) or equivalent document, IB supplement (if applicable).
- That I am aware of, and will comply with, "Good Clinical Practice" (GCP) and all applicable regulatory requirements.
- That I have been informed that certain regulatory authorities require Neovacs SA to obtain and supply, as necessary, details about the Investigator's ownership interest in

Neovacs SA or the investigational product, and more generally about his/her financial ties with Neovacs SA. Neovacs SA will use and disclose the information solely for the purpose of complying with regulatory requirements.

Hence I:

- Agree to supply Neovacs SA with any necessary information regarding ownership interest and financial ties (including those of my spouse and dependent children).
- Agree to promptly update this information if any relevant changes occur during the course of the study and for 1 year following completion of the study.
- Agree that Neovacs SA may disclose any information it has about such ownership interests and financial ties to regulatory authorities.
- Agree to provide Neovacs SA with an updated Curriculum Vitae.

Agreed by:

Investigator: ______________________________

______________________________ ______________________________

Investigator signature **Date**

Synopsis

Synopsis amended 29 November 2010

**Title**	A phase II, randomized, double-blind, controlled study to evaluate the immune responses, safety and clinical efficacy of three doses of Neovacs' TNF-Kinoid in adult patients with rheumatoid arthritis who have relapsed despite anti-TNFα biological therapy.
**Indication/Study population**	Two or three injections of TNF-Kinoid (TNF-K) at three dose levels in adult patients between 18 and 70 years old with rheumatoid arthritis who have relapsed despite anti-TNFα biological therapy.
**Rationale**	Rheumatoid arthritis (RA) is a chronic systemic inflammatory auto-immune disease that primarily affects the joints but can also induce systemic symptoms in patients.<br><br>For a long time the therapeutic strategy of RA followed the step-up treatment pyramid, starting with non-steroidal anti-inflammatory drugs (NSAIDs) for months to years before initiating second line disease modifying anti-rheumatic drugs (DMARDs) such as methotrexate (MTX), when there was evidence of joint damage. More recently, monoclonal antibodies (mAbs) against tumor necrosis factor alpha (TNFα) have demonstrated very good efficacy in RA patients, especially in combination with MTX.<br><br>However these treatments also present shortcomings. Patients who develop antibodies against TNFα antagonist mAbs have a high probability of escaping mAb efficiency (Radstake 2008). In such cases, the current therapeutic practice is to switch to another biological DMARD, such as anti-TNFα or another biological treatment. These switches however create another risk of developing multiple resistances against multiple biological therapies. In addition switching therapy can erode the confidence of the patients and their compliance to an already cumbersome treatment (van Vollenhoven 2004).<br><br>The objective of this trial is to demonstrate that active immunization with anti-TNFα kinoid (TNF-K) is able to induce polyclonal anti-TNFα antibodies in RA patients who were previously treated with anti-TNFα mAb but have lost ability to respond to therapy (i.e. secondary failure). Indeed the polyclonal response induced by active immunization with TNF-K should overcome the secondary resistance and could become a treatment of choice for "switch therapy" in such patients.

The study is designed to identify the best dose and injection schedule in terms of anti-TNFα antibody response and safety by comparing schedules of two or three injections of TNF-K at three dose levels. The doses of TNF-K and the staggered dose increase scheme, have been selected based on the clinical experience gained in the ongoing phase I-II study in Crohn's disease (CD) patients. Doses 180 and 360 mcg have been shown to be safe and to induce an antibody response in patients with CD who were naïve to anti-TNFα therapy or who had successfully responded. A dose of 60 mcg was poorly immunogenic in that study. Follow-up over 12 months minimum will evaluate the persistence of the immune response and could allow determination of the timing for subsequent doses of TNF-K.

Although the sample size should not allow significant characterization of the clinical efficacy of TNF-K in this population, exploratory clinical data will be collected with well defined and validated disease scores and by measuring biological parameters.

Objectives

The primary objective of this study is:

- To identify the best dose and schedule of administration of TNF-K in terms of anti-TNFα antibody response induced by two or three injections of TNF-K (Day 0, 28 or Day 0, 7, 28) at three dose levels (90, 180 or 360 mcg).

The secondary objectives are:

- To characterize the immune response after two or three injections at each dose level until the anti-TNFα antibody response declines to ≤ cut off level and at least until Month12.
- To evaluate the clinical response to immunization with TNF-K.
- To assess the safety after two or three injections at each dose level.

Study design

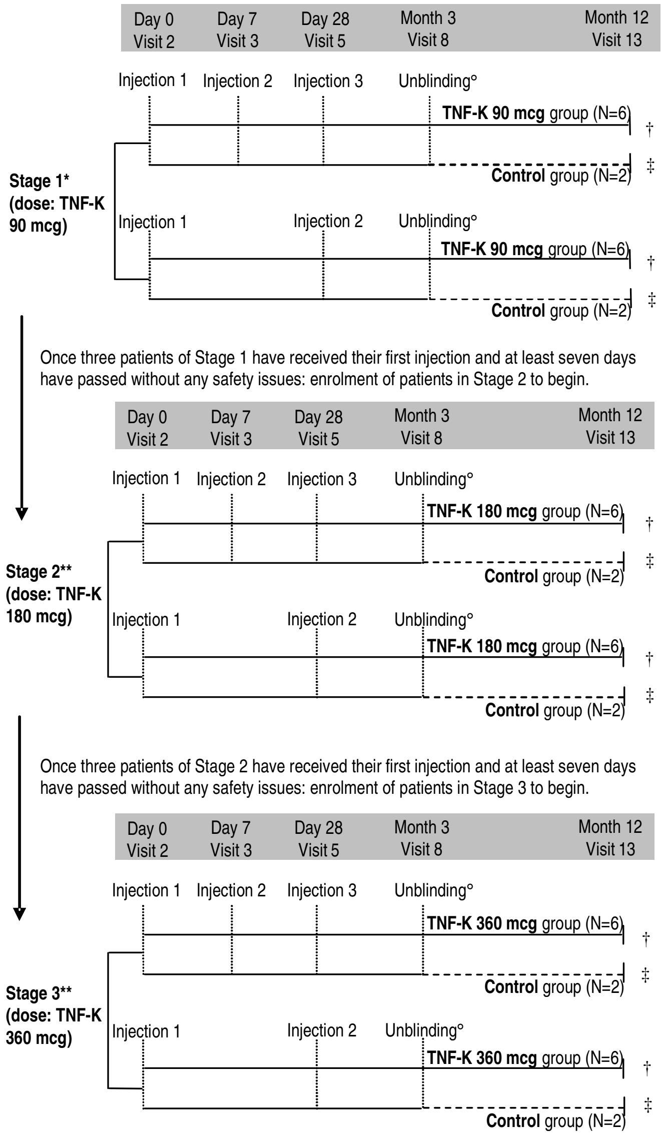

* An interval of one week will be respected between the first three patients. In addition, recruitment will only ***continue*** if at least three out of the ***initial group of*** patients have a positive anti-TNFα antibody response (3x increase vs ***cut off*** level) at Day 38.

** An interval of one week will be respected between the first three patients. Afterwards, there will be no limitations in the recruitment process.

° Unblinding will be carried out within a dose level after all patients randomized to that dose level have completed their Month 3 visit or been prematurely withdrawn from the study. Rescue therapy may be provided, if required, to any patient as of the Month 3 visit.

† TNF-K group patients will be followed up until the anti-TNFα antibody response declines to ≤ ***cut off*** level (***i.e. level at Day 0***) and at least until Month 12.

‡ Control group patients will be followed up until Month 12. During follow up (dashed line), they will undergo all study procedures, except those related to immune response to TNF-K.

Experimental design:

- Phase II, randomized, double-blind, multicenter, controlled international clinical trial.
- Two immunization schedules:
    - Day 0 and Day 28.
    - Day 0, Day 7 and Day 28.
- Four study groups:
- TNF-K 90 mcg group: 6-12 patients receiving TNF-K at 90 mcg combined with adjuvant ISA-51.
- TNF-K 180 mcg group: 12 patients receiving TNF-K at 180 mcg combined with adjuvant ISA-51.
- TNF-K 360 mcg group: 12 patients receiving TNF-K at 360 mcg combined with adjuvant ISA-51.
- Control group: 10-12 patients receiving mannitol powder combined with adjuvant ISA-51.

Staggered dose increase design:

Stage 1:

- 8-16 patients will be recruited to receive either TNF-K at 90 mcg (6-12 patients) or a control (2-4 patients). Study products will be administered following two different schedules (Day 0, 28 or Day 0, 7, 28).
- An interval of one week will be respected between the first three patients (the fourth patient can then be recruited immediately after the third and so on). Recruitment in stage 1 will be temporarily stopped after the 8th patient is randomized. The anti-TNFα antibody levels of this initial group of patients at baseline and at Day 38 will be titrated by ELISA. The Independent Data and Safety Monitoring Board (IDSMB) will assess blinded results (only the total number of patients in Stage 1 with a positive anti-TNFα antibody response at Day 38 will be disclosed) and will decide on the continuation of the recruitment in Stage 1. The IDSMB will concomitantly review the safety data of these patients.
- The enrolment in Stage 1 will continue if three or more patients out of the initial group of patients present a

positive anti-TNFα antibody response as defined by a three-fold increase at Day 38 compared to cut-off.

- If the IDSMB detects a safety issue or if an investigational product related serious adverse event is reported, the enrolment in that stage and/or in the study will be put on hold for further evaluation.

- Once three patients of Stage 1 have received at least one injection since at least seven days and no safety issues have been reported, the enrolment in Stage 2 will start and enrolment in Stage 1 will continue in parallel (4:2 ratio for Stage1:Stage2 randomization ensuring that only 1 in every 4 patients randomized consecutively in a stage is administered the control).

Stage 2

- 16 patients will be recruited to receive either TNF-K at 180 mcg (12 patients) or a control (4 patients). Study products will be administered following two different schedules (Day 0, 28 or Day 0, 7, 28).

- An interval of one week will be respected between the first three patients (the fourth patient can then be recruited immediately after the third and so on).

- Once three patients of Stage 2 have received at least one injection, since at least seven days and no safety issues have been reported, the enrolment in Stage 3 will start and enrolment in Stage 2 (and Stage 1, if applicable) will continue in parallel ([4:]2:1 ratio for [Stage1:]Stage2:Stage3 randomization ensuring that only 1 in every 4 patients randomized consecutively in a stage is assigned to the control group).

Stage 3

- 16 patients will be recruited to receive either TNF-K at 360 mcg (12 patients) or a control (4 patients). Study products will be administered following two different schedules (Day 0, 28 or Day 0, 7, 28).

- An interval of one week will be respected between the first three patients (the fourth patient can then be recruited immediately after the third and so on).

- Unblinding will be carried out by dose level when the last patient of each stage has completed the Month 3 visit.

- For ethical reasons, rescue therapy will be proposed at the discretion of the treating physician to any patients as of the Month 3 visit, if required (before unblinding: any treatment except anti-TNFα therapy; after unblinding: any treatment for the patients of the placebo group and any treatment except anti-TNFα therapy for the patients of the TNF-K group).

- If a patient has a medical need for rescue treatment prior to the Month 3 visit, the study treatment should be permanently discontinued (if applicable) and rescue treatment administered at the discretion of the investigator. The patient should still be followed up in the study. Furthermore, a new patient may replace this patient upon discussion between the International Coordinating Investigator and the Sponsor (the assignment of the new patient to the same treatment will be managed automatically by the Interactive Voice Response System [IVRS]). The patients discontinuing the study after Month 3 will not be replaced.

- TNF-K group patients will be followed up until the anti-TNFα antibody response declines to ≤ cut off levels and at least until Month 12.

- Beyond the Month 3 visit and following unblinding of their stage, placebo group patients will be followed for safety monitoring until Month 12. They will return at each planned visit to undergo all study procedures, except those related to immune response to TNF-K (anti-TNFα antibodies, anti-KLH antibodies, neutralizing antibodies and T cell response).

Treatment allocation:

- Randomization 3:1 between TNF-K group and control group within each stage. Patients dropping out or receiving rescue treatment prior to Month 3 may be replaced upon discussion between the International Coordinating Investigator and the Sponsor. Patients discontinuing the study after Month 3 will not be replaced.

- Randomization of patients may be split between two or three stages due to the staggered method of enrolment. The IVRS will ensure that patients will be randomized in a 4:2(:1) ratio (Stage1:Stage2, Stage2: Stage3 or Stage1:Stage2:Stage3) until the necessary number of patients have been randomized to each dose stage.

Blinding: Double-blind study within dose level until completion of Month 3 visit by the last patient of each stage.

Control: The control consists of mannitol powder with adjuvant ISA-51.

Data collection: The data is collected on a Case Report Form (CRF).

The Independent Data and Safety Monitoring Board (IDSMB) will also perform three safety data reviews by dose-injection schedule when all patients in the study have completed their Month 3 (or premature end of study [EOS]) visit, their Month 6 (or premature EOS) visit and at study completion.

Schedules	Groups				
	TNF-K 90 mcg	TNF-K 180 mcg	TNF- K 360 mcg	Control	Total
**Days: 0, 28**	6	6	6	6	24
**Days: 0, 7, 28**	6	6	6	6	24
**Total**	12	12	12	12	48

Number of patients

The total cohort will include 40-48 patients with a maximum of 12 patients in each TNF-K group and of 12 patients in the control group.

Primary endpoint

Immune responses

- Proportion of patients with at least a 3-fold increase in antibody response to TNFα vs cut-off at Day 38.

Secondary endpoints

Interim analysis at Month 3

Clinical efficacy

- Proportion of patients with a ≥1.2 decrease in DAS28 at Month 3 vs baseline (Day 0).
- Absolute change in DAS28 at Month 3 vs baseline (Day 0).
- Absolute change in Swollen Joint Counts (SJC) and in Tender Joint Counts (TJC) at Month 3 vs baseline (Day 0).
- Proportion of patients achieving ACR20, ACR 50 and ACR 70 at Month 3 vs baseline (Day 0).
- Proportion of patients with a good/moderate EULAR response at Month 3 vs baseline (Day 0).

- Absolute change in CRP level at Month 3 vs baseline (Day 0).
- Absolute change in ESR at Month 3 vs screening.
- Proportion of patients with or without ADAs at screening with a ≥1.2 decrease in DAS28 at Month 3 vs baseline ***(Day 0)***.

Immune response

- Proportion of patients with at least a 3-fold increase in antibody response to TNFα vs cut-off at Month 2 (Day 56) and at Month 3 (Day 84).
- Proportion of patients with a positive neutralizing antibody response.

Safety analysis

- Occurrence, intensity and relationship to TNF-K immunization of any solicited local and general signs and symptoms during a seven-day follow up period (i.e. day of immunization and 6 subsequent days) after each TNF-K injection.
- Occurrence, intensity and relationship to TNF-K immunization of unsolicited local and general signs and symptoms occurring until Month 3.
- Occurrence and relationship to TNF-K immunization of all SAE occurring until Month 3.
- Change from screening in hematological and biochemical levels in all groups at Month 3.
- Hematological and biochemical levels within or outside the normal ranges in all groups.

<u>Final analysis at Month 12</u>

Clinical efficacy

- Proportion of patients with a ≥1.2 decrease in DAS28 vs baseline (Day 0).
- Absolute change in DAS28 vs baseline (Day 0).

- Absolute change in Swollen Joint Counts (SJC) and in Tender Joint Counts (TJC) vs baseline (Day 0).

- Proportion of patients with ACR20, ACR 50 and ACR 70 vs baseline (Day 0).

- Proportion of patients with a good/moderate EULAR response vs baseline (Day 0).

- Absolute change in CRP level vs baseline (Day 0).

- Absolute change in ESR vs screening.

- Proportion of patients with a ≥1.2 decrease in DAS28 score vs DAS28 score decrease observed under previous treatment with a TNFα antagonist.

- Proportion of patients with the same maximum clinical improvement in terms of DAS28 observed during previous treatment with a TNFα antagonist.

- Absolute change in DAS28 vs DAS28 absolute change observed under previous treatment with a TNFα antagonist.

- Absolute change in SJC and in TJC vs SJC and TJC absolute change observed under previous treatment with a TNFα antagonist.

- Proportion of patients with ACR 20, ACR 50 and ACR 70 vs status observed under previous treatment with a TNFα antagonist.

- Proportion of patients with a good/moderate EULAR response vs status observed under previous treatment with a TNFα antagonist.

- Proportion of patients withdrawn for lack of efficacy.

Immune responses

The timings of blood samplings and analyses are specified in Table 2 and Table 5.

- Anti-TNFα antibody concentrations.

- Proportion of good responders defined as patients with dilution of anti-TNFα antibodies titers ≥2000

- Proportion of patients with a positive anti-TNFα

neutralizing antibody response.

- Anti-TNFα neutralizing antibody levels.
- Anti-KLH antibody concentrations.
- Absolute changes in levels of cytokines (TNFα, TNF- RII, IL-6, IL-17, IL-23 and others) vs baseline (Day 0).
- Proportion of patients with a positive T cell response as measured by lymphoproliferation.

Safety *analysis*

- Occurrence, intensity and relationship to TNF-K immunization of unsolicited local and general signs and symptoms occurring throughout the study period.
- Occurrence and relationship to TNF-K immunization of all SAE occurring throughout the study period.
- Change from screening in hematological and biochemical levels in all groups.
- Hematological and biochemical levels within or outside the normal ranges in all groups.

TABLE OF CONTENT(S)

1. INTRODUCTION 25
1.1. Background 25
1.2. Study rationale 27
1.3. Rationale for Amendment 01, dated 03 May 2010 28
1.4. Rationale for amendment 02, dated 29 November2010: 29

2. OBJECTIVES 30
2.1. Primary objective(s) 30
2.2. Secondary objective(s) 30

3. STUDY DESIGN OVERVIEW 31

4. STUDY POPULATION 34
4.1. Number of patients and characteristics 34
4.2. Recruitment method 34
4.3. Inclusion criteria for enrolment 34
4.4. Exclusion criteria for enrolment 35
4.5. Elimination criteria during the study 37
4.6. Contraindications to subsequent TNF-K/control administration 38

5. CONDUCT OF THE STUDY 39
5.1. Patient identification 39
5.2. Outline of study procedures 40
5.3. Detailed description of study procedures 42
5.3.1. Screening Visit (Visit 1) 42
5.3.2. Informed consent 43
5.3.3. Eligibility criteria 43
5.3.4. Medical history 43
5.3.5. Tuberculosis testing 44
5.3.6. Physical examination and vital signs 44
5.3.7. IVRS calls 45
5.3.8. Randomization 46
5.3.9. Contraindications 46
5.3.10. TNF-K administration 46
5.3.11. Elimination criteria 47
5.3.12. Blood sampling 47
5.3.13. Pregnancy test 49
5.3.14. Recording of concomitant medication 49
5.3.15. Recording of non-serious adverse events (AEs) and serious adverse events (SAEs) 49
5.3.16. Interim analysis and study conclusion 49
5.4. Sample handling and analysis 50
5.4.1. Sample volumes and handling 50
5.4.2. Laboratory assays 51
5.5. Restrictions 52
5.5.1. Prior and concomitant medications 52
5.5.2. Blood donation 52
5.5.3. Clinical trial restrictions 52
5.6. Compliance control and monitoring 53
5.6.1. Patient compliance 53
5.6.2. Site compliance and monitoring 53

5.6.3. Medical monitoring 54
5.6.4. Pharmacovigilance 54
5.6.5. Independent Data and Safety Monitoring Board 54

6. INVESTIGATIONAL PRODUCT AND ADMINISTRATION 55
6.1. Study products 55
6.2. Dosage and administration 55
6.3. Packaging and labeling 56
6.4. Storage, reconstitution and return 56
6.5. Treatment allocation and randomization 58
6.5.1. Randomization of supplies 58
6.5.2. Randomization of patients 59
6.6. Method of blinding and breaking the study blind 59

7. ADVERSE EVENTS AND SERIOUS ADVERSE EVENTS 60
7.1. Definitions 60
7.1.1. Adverse event 60
7.1.2. Intensity of event 60
7.1.3. Relationship to study products 61
7.1.4. Serious adverse event 62
7.2. Adverse event recording 62
7.2.1. Diary cards 63
7.2.2. *Recording of* solicited adverse events *by study staff* 63
7.3. Assessment of AE intensity 64
7.3.1. Local reactions 64
7.3.2. Systemic reactions 64
7.3.3. Reactions assessed by laboratory parameters 65
7.4. Adverse event *and pregnancy* reporting 66
7.5. Emergency and rescue medication 68

8. SUBJECT WITHDRAWAL AND STOPPING RULES 68
8.1. Withdrawal of patients on the basis of safety 68
8.2. Stopping rules on the basis of safety 69

9. STATISTICAL ANALYSES 70
9.1. Planned analyses 70
9.2. Primary endpoint 70
9.3. Secondary endpoints 70
9.3.1. Interim analysis at Month 3 70
9.3.2. Final analysis at Month 12 71
9.4. Estimated sample size 73
9.5. Analysis populations 73
9.6. Statistical considerations 74
9.7. Statistical Methods 75
9.7.1. Demographic, baseline and follow-up characteristics 75
9.7.2. Immune responses 75
9.7.3. Clinical efficacy 76
9.7.4. Safety 77

10. DOCUMENTATION OF DATA 78
10.1. *C*ase report forms (*CRF*s) 78
10.2. Data entry in database 78
10.3. Coding of adverse events, drugs and diseases 78
10.4. Reporting and communication of results 79

11. REGULATORY AND ETHICAL ISSUES 79
11.1. General legal references 79
11.2. Insurance and Indemnity 79
11.3. Ethics committee 79
11.4. Protocol adherence - amendments 79
11.5. Required documents 80
11.6. Informed consent 80
11.7. Subject data protection 80
11.8. Quality assurance 80
11.9. Record retention 81
11.10. Confidentiality 81
11.11. Publications 81

12. REFERENCES 83

LIST OF FIGURES

PAGE

Figure 1 Staggered dose design 333

LIST OF TABLES

PAGE

Table 1 Maximum number and distribution of patients enrolled 34
Table 2 List of study procedures 40
Table 3 Time window between study visits 42
Table 4 Exact timing of the IVRS calls *(Amended 29 November2010)* 46
Table 5 Laboratory assays 51
Table 6 Permitted medication 52
Table 7 Disallowed medications and wash-out periods *(Amended 29 November2010)* 52
Table 8 Study products 55
Table 9 Dosage and administration of study products 56
Table 10 Intensity scale for local reactions 64
Table 11 Intensity scale for quantitative systemic reactions 64
Table 12 Intensity scale for subjective systemic reactions 65
Table 13 Intensity scale of reactions assessed by laboratory parameters 66

LIST OF APPENDICES

PAGE

Appendix 1 Clinical scores and questionnaire 87

Appendix 2 WORLD MEDICAL ASSOCIATION DECLARATION OF HELSINKI 88

LIST OF ABBREVIATIONS

(Amended 29 November 2010)

**ACR**	American College of Rheumatology
**ADA**	Anti-Drug Antibodies
**AE**	Adverse Event
**ALT**	Alanine aminotransferase
**AST**	Aspartate aminotransferase
**BCG**	Bacillus Calmette-Guérin
**CCP**	Cyclic Cirullinated Peptides
**CD**	Crohn's Disease
**CI**	Confidence Interval
**CMI**	Cell Mediated Immunity
**CPK**	Creatine PhosphoKinases
**CRA**	Clinical Research Associate
**CRF**	Case Report Form
**CRO**	Contract Research Organization
**CRP**	C-Reactive Protein
**DAS**	Disease Activity Score
**DMARD**	Disease Modifying Anti-Rheumatic Drug
**DMF**	Drug Master File
**EC**	Ethics Committee
**ECG**	ElectroCardioGram
**ELISA**	Enzyme-Linked Immunosorbent Assay
**EOS**	End Of Study
**ESR**	Erythrocyte Sedimentation Rate
**EULAR**	EUropean League Against Rheumatism
**FAS**	Full Analysis Set
**FDA**	Food and Drug Administration
**GCP**	Good Clinical Practice
**GMT**	Geometric Mean Titer
**cGMP**	current Good Manufacturing Practice
**HACA**	Human Anti Chimeric Antibody
**HAHA**	Human Anti Human Antibody
**HBc**	Hepatitis B core
**HBsAg**	Hepatitis B surface Antigen
**HBV**	Hepatitis B Virus
**hCG**	human Chorionic Gonadotropin
**HCV**	Hepatitis C Virus
**HIV**	Human Immunodeficiency Virus
**ICF**	Informed Consent Form
**ICH**	International Conference on Harmonization
**IDES**	Internet Data Entry System
**IDSMB**	Independent Data and Safety Monitoring Board
**IEC**	Independent Ethics Committee
**IFN**	InterFeroN
**IgG**	Immunoglobulin G
**IgM**	Immunoglobulin M

**IL**	Interleukin
**IM**	IntraMuscular
**IN**	Institutional Normal
**IND**	Investigational New Drug
**IRB**	Institutional Review Board
**IVRS**	Interactive Voice Response System
**KLH**	Keyhole Limpet Hemocyanin
**LDH**	Lactate DeHydrogenase
**LLT**	Lowest Level Term
**mAb**	monoclonal Antibody
**mcg**	micrograms
**MCP**	MetaCarpoPhalangeal
**MedDRA**	Medical Dictionary for Regulatory Activities
**MMP**	Matrix MetalloProteinases
**MTX**	Methotrexate
**NSAID**	Non-Steroidal Anti-Inflammatory Drug
**PI**	Principal Investigator
**PPD**	Purified Protein Derivative
**PPS**	Per Protocol Set
**PT**	Preferred Term
**RA**	Rheumatoid Arthritis
**RBC**	Red Blood Cell
**RF**	Rheumatoid Factor
**RIA**	RadioImmunoAssay
**SAF**	Safety Set
**SAP**	Statistical Analysis Plan
**SAS®**	Statistical Analysis System
**SJC**	Swollen Joint Count
**SOC**	System Organ Class
**SOP**	Standard Operating Procedures
**TB**	Tuberculosis
**TBD**	To be determined
**TJC**	Tender Joint Count
**TNFα**	Tumor Necrosis Factor alpha
**TNF-K**	Tumor Necrosis Factor Kinoid
**ULN**	Upper Limit of Normal
**VAS**	Visual Analog Scale
**WBC**	White Blood Cell
**WFI**	Water For Injection

GLOSSARY OF TERMS

**Adverse event**	Any untoward medical occurrence in a patient or clinical investigation patient, temporally associated with the use of a medicinal product, whether or not considered related to the medicinal product.<br><br>An AE can therefore be any unfavorable and unintended sign (including an abnormal laboratory finding), symptom, or disease (new or exacerbated) temporally associated with the use of a medicinal product. For marketed medicinal products, this also includes failure to produce expected benefits (i.e. lack of efficacy), abuse or misuse.
**Blinding:**	A procedure in which one or more parties to the trial are kept unaware of the treatment assignment in order to reduce the risk of biased study outcomes. In a single-blind trial, the Investigator and/or his staff are aware of the treatment assignment but the patient is not. In an observer-blind study, the patient and the study personnel involved in the clinical evaluation of the patients are blinded while other study personnel may be aware of the treatment allocation. When the Investigator and Sponsor staff who are involved in the treatment or clinical evaluation of the patients and review/analysis of data are also unaware of the treatment assignments, the study is double-blind. Partially-blind is to be used for study designs with different blinding levels between different groups, e.g. double-blinded consistency lots which are open with respect to the control group. The level of blinding is maintained throughout the conduct of the trial, and only when the data are cleaned to an acceptable level of quality will appropriate personnel be unblinded or when required in case of a serious adverse event.
**Eligible:**	Qualified for enrolment into the study based upon strict adherence to inclusion/exclusion criteria.
**Evaluable:**	Meeting all eligibility criteria, complying with the procedures defined in the protocol and, therefore, included in the Per Protocol Set (PPS).
**Independent Data and Safety Monitoring Board**	The IDSMB is an independent committee appointed to oversee ethical and safety aspects of the conduct of the study.
**Investigational product:**	A pharmaceutical form of an active ingredient or placebo being tested or used as a reference in a clinical trial, including a product with a marketing authorization when

	used in a way different from the approved form, or when used for an unapproved indication, or when used to gain further information about an approved use.
**Medical Monitor:**	An individual medically qualified to assume the responsibilities of the Sponsor (Neovacs) especially in regards to the ethics, clinical safety of a study and the assessment of adverse events.
**Protocol amendment:**	ICH defines a protocol amendment as: "A written description of a change(s) to or formal clarification of a protocol." Neovacs further details this to include a change to an approved protocol that affects the safety of patients, scope of the investigation, study design, or scientific integrity of the study.
**Protocol administrative change:**	A protocol administrative change addresses changes to only logistical or administrative aspects of the study. N.B. Any change that falls under the definition of a protocol amendment (e.g. a change that affects the safety of patients, scope of the investigation, study design, or scientific integrity of the study) MUST be prepared as an amendment to the protocol.
**Site Monitor:**	An individual assigned by the Sponsor who is responsible for assuring proper conduct of clinical studies at one or more investigational sites.
**Solicited adverse event:**	Adverse events (AEs) to be recorded as endpoints in the clinical study. The presence/occurrence/intensity of these events is actively solicited from the patient or an observer during a specified post-immunization follow-up period.
**Study Monitor:**	An individual assigned by the Sponsor who is responsible for assuring proper conduct of a clinical study.
**Patient:**	Term used throughout the protocol to denote an individual that has been contacted in order to participate or participates in the clinical study, either as a recipient of the investigational product(s) or as a control.
**Patient number:**	A unique number identifying a patient, assigned to each patient consenting to participate in the study.
**Treatment:**	Term used throughout the clinical study to denote a set of investigational product(s) or marketed product(s) or placebo intended to be administered to a patient, identified by a unique number, according to the study randomization or treatment allocation.

**Treatment number:**	A unique number identifying a treatment box assigned to a patient, according to the study randomization or treatment allocation. For this study, one treatment number will be assigned for one injection at dose levels 90 and 180 mcg and two treatment numbers will be assigned for the two injections at dose level 360 mcg.
**Unsolicited adverse event**	Any adverse event (AE) reported in addition to those solicited during the clinical study. Also any "solicited" symptom with onset outside the specified period of follow-up for solicited symptoms will be reported as an unsolicited adverse event.

1. INTRODUCTION

1.1. Background

Rheumatoid arthritis (RA) is a chronic systemic inflammatory auto-immune disease that primarily affects the joints but can also induce systemic symptoms.

The disease is present in all ethnic groups. The prevalence estimates of RA range from 1% to 5% with rates varying between ethnicities and countries. There is a gender ratio imbalance with 2,5 times more women affected. The average incidence in the USA is 0.5/1000 person/year (Drosos 2004). The disease can appear at all ages but the peak incidence occurs within the fourth and fifth decades of life.

Joint involvement in RA is typically symmetrical in nature with an insidious onset. Usually the first joints to be affected are the wrists and the joints of hand and fingers (metacarpophalangeal [MCP] and interphalangeal joints). As the disease progresses, larger joints such as ankles, knees, elbows and shoulders are involved. Morning stiffness lasting more than one hour is a hallmark symptom of early disease, as well as swelling and tenderness of the joints (Tehlirian 2002).

If left untreated or inadequately treated, RA will cause extensive and irreversible joint damage and chronic pain. Important deformation of the hands with ulnar deviation of the fingers, dorsal subluxation of the MCP joints, loss of extension of the elbows and the wrists and other deformations are caused by chronic synovitis, loss of cartilage and bone erosion.

It is now accepted that these changes occur rapidly after the onset of disease. Indeed the rate of progression is most prominent during the first two years of follow-up (Fuch 1989; van der Heyden 1995). Afterwards, the disease continues to progress at a steady rate and disability and radiographically revealed damage are correlated at late stages of disease (Sharp 1991; Scott 2000).

In addition to this evolving disability, RA is associated with an approximately two fold increased mortality (Wolfe 1994) and with high rates of co-morbid conditions including malignancies, infections and cardiovascular diseases (Wolfe 2003; Doran 2002; Smitten 2008). Control of inflammatory disease is not sufficient to stop the progression of structural joint damages as observed by imaging studies. Indeed, patients with stable inflammatory indicators may still show progressive destructive changes on images, as if the pathogenesis of synovial inflammation may differ from that of articular erosions (Callahan 1997, Scott 1984).

For a long time, the therapeutic strategy of RA followed the step-up treatment pyramid, starting with non-steroidal anti-inflammatory drugs (NSAIDs) for months to years before initiating second line disease modifying anti-rheumatic drugs (DMARDs) when there was evidence of joint damage. However this approach only leads to short-term control of inflammation and symptom relief and most often to long-term joint destruction, severe functional decline and premature mortality. DMARDs were eventually introduced earlier and more aggressively after demonstration that they slowed down joint destruction and disability more efficiently (Emery 2002; Ward 1990). This change in strategy was

reinforced by studies showing that traditional DMARDs such as methotrexate (MTX) or leflunomide can delay radiographic disease progression (Strand 1999; Smolen 1999; Emery 2000).

Despite these positive results, monotherapy with traditional DMARDs often fails to fully control the symptoms and the evolution of RA. Combinations of drugs are therefore the rule to improve efficacy. However traditional DMARDs are associated with significant toxicity which may limit their use: hepatotoxicity, pulmonary fibrosis and myelosuppression with MTX; persistent diarrhea and weight loss with leflunomide; renal toxicity and hypertension with cyclosporine. This toxicity profile leads to high rates of patient withdrawal from therapy (Aletaha 2002). In addition traditional DMARDs are not fully effective in preventing joint erosion and they poorly improve survival.

The proinflammatory cytokine tumor necrosis factor alpha (TNFα) is a key mediator in the pathogenesis of RA. TNFα is primarily produced by activated monocytes and macrophages and it mediates both inflammatory synovitis and articular matrix degradation (Choy 2001). TNFα produces its deleterious effects through various mechanisms: it induces the production of pro-inflammatory cytokines, stimulates endothelial cells to express adhesion molecules that attract leukocytes into affected joints, increases the rate of synthesis of metalloproteinases by synovial macrophages, fibroblasts, osteoclasts and chondrocytes, and inhibits the synthesis of proteoglycans in cartilage.

Monoclonal antibodies (mAb) against TNFα have demonstrated very good efficacy in RA patients, especially in combination with MTX (Olsen 2004; Scott 2006). They showed rapid onset of action (within one to two weeks), substantial improvement in the signs and symptoms of disease for a high proportion of patients, reduction in disability, improved health related quality of life, inhibition of radiographic progression of disease and sustained efficacy over several years (Maini 1999; Weinblatt 1999, 2003; Keystone 2002; Lipsky 2000).

However these treatments also present shortcomings. All current therapies remain cumbersome to use and side effects of non biological DMARDs still impair the compliance of patients. Furthermore a substantial proportion of patients does not respond to TNFα antagonists (about 10-30% of primary non-responders) and another part loose clinical efficacy over time (up to 40% of secondary non-responders) in part due to the occurrence of antibodies against the TNFα antagonists (Anderson 2005). Active immunization against TNFα is an alternative therapeutic strategy that could allow these limitations to be overcome. Indeed neutralization of the excess TNFα through induction of self polyclonal antibodies is an elegant therapeutic option. First, polyclonal antibodies should be effective in patients who developed secondary resistance which is often associated with antibodies against therapeutic mAbs (such as Human Anti Chimeric Antibodies [HACAs] and Human Anti Human Antibodies [HAHAs] also known as anti-drug-antibodies [ADAs]). These antibodies are highly prevalent when the administered monoclonal antibody is chimeric, like infliximab, with a prevalence reaching up to 61 % (Baert, 2003) but they have also been detected in patients receiving "human" mAb like adalimumab in up to 17% of patients after only six months of treatment (Bartelds 2007). The synergy observed between mAbs and MTX could be at least partially explained by the reduction in HACAs and/or HAHAs induced by the immuno-suppressing properties of MTX. Second, active immunization should not induce secondary resistance. Third,

since approximately one third of patients with a primary resistance to one mAb will respond to another mAb (Buch 2007), it is likely that they will respond even better to the polyclonal response induced by active immunization. Fourth, the side-effects reported with mAb mainly due to the "bolus" high blood concentrations after administration should not be observed since the generation of antibodies following active immunization is more physiologically progressive. Furthermore, severe infusion reactions are usually reported when anti drug antibodies are present. Finally, the treatment maintenance with active immunization is expected to require only few administrations per year hence improving long term compliance to treatment.

Neovacs has prepared an adjuvanted kinoid formulation to induce polyclonal anti-TNFα self antibodies. TNF-Kinoid (TNF-K) has been studied in several preclinical efficacy and toxicology models. In a transgenic mouse model of RA, administration of TNF-K has successfully controlled the disease, both before the appearance of clinical symptoms (Le Buanec 2006) and after the onset of the disease (data on file). The absence of toxicity has been demonstrated in numerous animal models (please refer to the Investigator Brochure).

The safety and immune responses of TNF-K are currently being evaluated in a phase I-II clinical study conducted in patients with Crohn's disease (CD). Few mild local reactions at the injection site have been reported and no safety concern has been identified after multiple administrations of increasing doses of TNF-K (60, 180 and 360 mcg).

1.2. Study rationale

Patients who develop antibodies against TNFα antagonist mAb have a high probability of escaping mAb efficiency (Radstake 2008). Current therapeutic practice is to switch to another biological DMARD, such as anti-TNFα or another biological treatment. These switches however may result in the development of multiple resistances against multiple biological therapies. In addition switching therapy can erode the confidence of the patients and their compliance to an already cumbersome treatment (van Vollenhoven 2004).

The objective of this trial is to demonstrate that active immunization with TNF-K is able to induce anti-TNFα antibodies in RA patients who were previously treated with anti-TNFα mAb but have lost the ability to respond to therapy. The study is designed to identify the best immune response dose in terms of anti-TNFα antibody response by comparing schedules of two or three injections of TNF-K at three dose levels. The doses of TNF-K and the staggered dose increase scheme, have been selected based on the clinical experience gained in the ongoing phase I-II study in CD patients. Doses 180 and 360 mcg have been shown to be safe and to induce an antibody response in CD patients with no previous anti-TNFα therapy or with no resistance to previous anti-TNFα therapy. A dose of 60 mcg was also evaluated and is poorly immunogenic. Follow-up over 12 months minimum will evaluate the persistence of the immune response and could allow determination of the timing for subsequent doses of TNF-K.

Although the sample size will not allow significant characterization of the clinical efficacy of TNF-K in this population, patients will be evaluated clinically with well defined and validated disease scores and by measuring biological parameters.

1.3. Rationale for Amendment 01, dated 03 May 2010

The protocol has been modified mainly to correct minor inconsistencies that were identified during the initiation of the trial.

Major changes include:

- Methotrexate concomitant therapy is no longer mandatory since patients may be treated by TNFα antagonists only or be intolerant to methotrexate. In such cases, it would not be ethical to impose initiation of methotrexate administration to allow entry in the study.
- The wash-out period after last administration of adalimumab and etanercept has been reduced to 4 weeks instead of 8 weeks, to account for the shorter half life of these two TNFα antagonists.
- Since the clinical scores(DAS28; ACR) are rarely recorded in clinical practice, the inclusion criterion of history of positive response with a previous TNFα antagonist treatment can also be defined by the investigators opinion.
- An interval of at least 4 weeks after last administration of infliximab and of at least 2 weeks since last administration of adalimumab is recommended before sampling for detection of anti drug antibodies (ADA). This will decrease the risk of interference by the circulating TNFα antagonists on the detection of ADA.
- Since hematology and biochemistry parameters are collected at screening (Visit 1) and not at baseline (Visit 2), the end points involving hematology and biochemistry parameters, including ESR, will be assessed vs status at screening.
- Patients with RA under corticosteroid therapy often present an increase in white blood cells over 15x10/L due to their RA disease without any other pathology. Therefore the upper limit of $15x10^9$/L for WBC has been deleted from the exclusion criteria.
- The optional procedure of synovial biopsy has been deleted because results generated in only a minority of study sites would not be interpretable.
- The duration of the screening period is maximum 30 days. If a patient is not included during that period, screening procedures will have to be repeated, with the exception of:
    - the detection of ADA if the initial assay is positive
    - the Mantoux test, provided the previous test was done within 365 days prior to first immunization and was negative,
    - the ECG, provided the previous ECG was done within 84 days prior to first immunization and did not show any clinically significant abnormalities,
    - the chest X-Ray, provided the previous chest X-Ray was done within 84 days prior to first immunization and did not show any clinically significant abnormalities,

    - the viral serology, provided the previous viral serology was done within 84 days prior to first immunization and was negative.

- The study will be opened in additional countries. Due to logistics constraints, the T cell response will not be evaluated in some far located countries.
- The laboratory methods for the testing of serum and plasma circulating cytokines and of ADA have been updated.
- The optional procedure of synovial biopsy has been deleted because only a minority of investigators are willing to perform it. Therefore, results would not be analyzable.

1.4. Rationale for amendment 02, dated 29 November2010

- ***Suppression of the inclusion criterion 9 : positive antibodies against anti-TNFα monoclonal antibodies at screening. The rationale supporting a better clinical response to a second anti-TNFα biological therapy after secondary resistance associated with ADAs is now controversial. A recent large study of certolizumab in patients with secondary resistance to infliximab did not detect any difference in clinical response between patients with or without ADAs at baseline (Sandborn 2010). The regulatory authorities have recommended broadening the target population to all patients with secondary resistance. As the recruitment of patients with ADAs has proven to be difficult, the detection of ADAs is not anymore required at screening. However, the patients will be tested at screening for ADAs positivity and a sub-analysis may be performed in ADA positive patients if numbers allow.***

- ***Secondary resistance to any anti-TNFα monoclonal antibody therapy is allowed. Monoclonal antibodies include infliximab, adalimumab, certolizumab, etanercept, golimumab. Patients may have been previously treated with one TNFα antagonist only.***

- ***The wash-out period between the last administration of infliximab or adalimumab and the blood sample for detection of ADAs is no longer required.***

- ***Clarification of the screening for tuberculosis. The national recommendations for tuberculosis screening vary between countries. It is not common practice to perform both the purified protein derivative skin test (Mantoux) and the IFNg EliSPOT (eg Quantiferon Gold). Therefore a patient who is negative by one of the two tests, with no history of tuberculosis and no suspicion of tuberculosis at chest X rays as defined in the protocol can be included. A patient who is positive by one of the two tests as defined by the protocol cannot be included.***

- ***Clarification of the dosage of concomitant anti rheumatic therapies. The dosage regimen of concomitant corticosteroid, methotrexate and non-steroidal anti-inflammatory drugs must remain unchanged during the study period until month 3.***

- ***Pregnancy is not recommended at any time during the whole study period.***

- ***T cell response: in order to detect late T cell response systematically, all patients will have blood collected and analyzed at day 140. The blood sample for T cell response at day 336 will only be performed in case of a positive T cell response at day 56 or 140 (as defined as a lymphoproliferation stimulation index ≥3 after stimulation of PBMCs with TNF).***

2. OBJECTIVES

(Amended 29 November2010)

The study will investigate the effects of two or three injections of TNF-K at three dose levels in adult patients with RA who have relapsed .

2.1. Primary objective(s)

The primary objective of this study is:

- To identify the best dose and schedule of administration of TNF-K in terms of anti-TNFα antibody response induced by two or three injections of TNF-K (Day 0, 28 or Day 0, 7, 28) at three dose levels (90, 180 or 360 mcg).

2.2. Secondary objective(s)

(Amended 03 May 2010)

The secondary objectives of this study are:

- To characterize the immune response after two or three injections at each dose level until the anti-TNFα antibody response declines to ≤ cut-off level and at least until Month12.
- To evaluate the clinical response to immunization with TNF-K.
- To assess the safety after two or three injections at each dose level.

3. STUDY DESIGN OVERVIEW

(Amended 03 May 2010)

This study will be a phase II, randomized, double-blind, controlled, multicenter, international clinical trial. It will follow a staggered dose scheme composed of three stages (see Figure 1):

Stage 1

- 8-16 patients will be recruited to receive either TNF-K at 90 mcg (6-12 patients) or a control (2-4 patients). Study products will be administered following two different schedules (Day 0, 28 or Day 0, 7, 28).
- An interval of one week will be respected between the first three patients (the fourth patient can then be recruited immediately after the third and so on). Recruitment in stage 1 will be temporarily stopped after the 8th patient is randomized. The anti-TNFα antibody levels of this initial group of patients at baseline and at Day 38 will be titrated by ELISA. The Independent Data and Safety Monitoring Board (IDSMB) will assess blinded results (only the total number of patients in Stage 1 with a positive anti-TNFα antibody response at Day 38 will be disclosed) and will decide on the continuation of the recruitment in Stage 1. The IDSMB will concomitantly review the safety data of these patients.
- The enrolment in Stage 1 will continue if three or more patients out of the initial group of patients present a positive anti-TNFα antibody response as defined by a three-fold increase at Day 38 compared to cut-off.
- If the IDSMB detects a safety issue or if an investigational product related serious adverse event is reported, the enrolment in that stage and/or in the study will be put on hold for further evaluation.
- Once three patients of Stage 1 have received at least one injection since at least seven days and no safety issues have been reported, the enrolment in Stage 2 will start and enrolment in Stage 1 will continue in parallel (4:2 ratio for Stage1:Stage2 randomization ensuring that only 1 in every 4 patients randomized consecutively in a stage is administered the control).

Stage 2

- 16 patients will be recruited to receive either TNF-K at 180 mcg (12 patients) or a control (4 patients). Study products will be administered following two different schedules (Day 0, 28 or Day 0, 7, 28).
- An interval of one week will be respected between the first three patients (the fourth patient can then be recruited immediately after the third and so on).
- Once three patients of Stage 2 have received at least one injection, since at least seven days and no safety issues have been reported, the enrolment in Stage 3 will start and enrolment in Stage 2 (and Stage 1, if applicable) will continue in parallel ([4:]2:1

ratio for [Stage1:]Stage2:Stage3 randomization ensuring that only 1 in every 4 patients randomized consecutively in a stage is assigned to the control group).

Stage 3

- 16 patients will be recruited to receive either TNF-K at 360 mcg (12 patients) or a control (4 patients). Study products will be administered following two different schedules (Day 0, 28 or Day 0, 7, 28).
- An interval of one week will be respected between the first three patients (the fourth patient can then be recruited immediately after the third and so on).
- Unblinding will be carried out by dose level when the last patient of each stage has completed the Month 3 visit.
- For ethical reasons, rescue therapy will be proposed at the discretion of the treating physician to any patients as of the Month 3 visit, if required (before unblinding: any treatment except anti-TNFα therapy; after unblinding: any treatment for the patients of the placebo group and any treatment except anti-TNFα therapy for the patients of the TNF-K group).
- If a patient has a medical need for rescue treatment prior to the Month 3 visit, the study treatment should be permanently discontinued (if applicable) and rescue treatment administered at the discretion of the investigator. The patient should still be followed up in the study. Furthermore, a new patient may replace this patient upon discussion between the International Coordinating Investigator and the Sponsor (the assignment of the new patient to the same treatment will be managed automatically by the Interactive Voice Response System [IVRS]). The patients discontinuing the study after Month 3 will not be replaced.
- TNF-K group patients will be followed up until the anti-TNFα antibody response declines to cut off levels and at least until Month 12.
- As from the Month 3 visit and unblinding of their stage, control group patients will be followed for safety monitoring until Month 12. They will return at each planned visit to undergo all study procedures, except those related to immune response to TNF-K (anti-TNFα antibodies, anti-KLH antibodies, neutralizing antibodies and T cell response).
- The IDSMB will also review the safety data when all patients have completed the Month 3 visit, the Month 6 visit and at study completion.

Figure 1 Staggered dose design

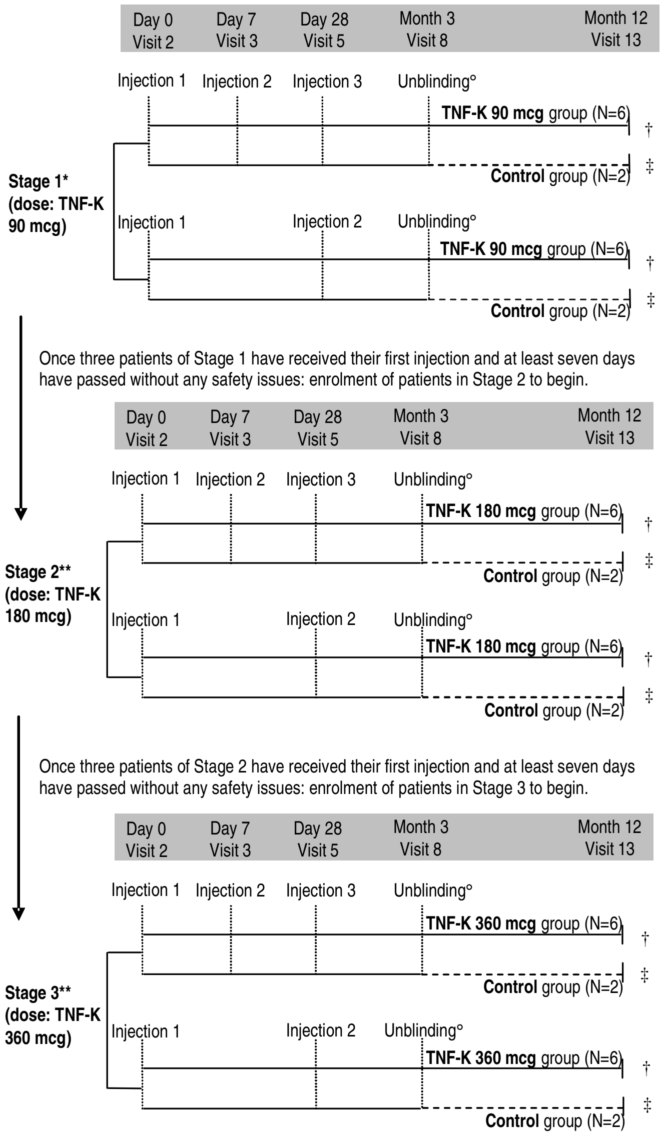

* An interval of one week will be respected between the first three patients. In addition, recruitment will only ***continue*** if at least three out of the ***initial group of*** patients have a positive anti-TNFα antibody response (3x increase vs ***cut off*** level) at Day 38.

** An interval of one week will be respected between the first three patients. Afterwards, there will be no limitations in the recruitment process.

° Unblinding will be carried out within a dose level after all patients randomized to that dose level have completed their Month 3 visit or been prematurely withdrawn from the study. Rescue therapy may be provided, if required, to any patient as of the Month 3 visit.

† TNF-K group patients will be followed up until the anti-TNFα antibody response declines to ≤ ***cut off*** level (***i.e. level at Day 0***) and at least until Month 12.

‡ Control group patients will be followed up until Month 12. During follow up (dashed line), they will undergo all study procedures, except those related to immune response to TNF-K.

4. STUDY POPULATION

4.1. Number of patients and characteristics

(Amended 29 November 2010)

The target number of eligible patients to be enrolled in this study is maximum 48. The patients, of either sex, and aged between 18 and 70 years, presenting with RA disease with a secondary loss of clinical response to anti-TNFα mAbs.

Each TNF-K group will include up to 12 patients and the control group will include 10-12 patients.

Table 1 Maximum number and distribution of patients enrolled

Schedules	Groups				
	TNF-K 90 mcg	TNF-K 180 mcg	TNF- K 360 mcg	Control	Total
Days: 0, 28	6	6	6	6	24
Days: 0, 7, 28	6	6	6	6	24
Total	12	12	12	12	48

4.2. Recruitment method

(Amended 03 May 2010)

This study will be performed in multiple centers. The recruitment rate will be monitored. The frequency of the monitoring visits will be adapted to the pace of enrolment with at least one visit per six weeks. Study product doses will be distributed respecting the randomization block size. Transfer of supplies will be tracked.

4.3. Inclusion criteria for enrolment

(Amended 29 November 2010)

Patients will be eligible for entry into the study if they meet the following criteria:

1. Diagnosis of RA according to the revised 1987 criteria of the American College of Rheumatology (ACR) (Arnett 1988) since at least six months prior to first study product administration.

2. Patients who the Investigator believes are able and willing to comply with the requirements of the protocol (e.g., completion of the diary cards, return for follow-up visits).

3. A male or female between 18 and 70 years of age at the time of the first immunization.

4. Active RA disease as evidenced by a Disease Activity Score 28 (DAS 28) $\geq$ 3.2.

5. History of treatment with TNFα antagonist (infliximab, adalimumab, etanercept, certolizumab, golimumab).

6. A wash-out period before the first administration of the study product of:

   - at least ten weeks since the last administration of certolizumab or golimumab
   - at least eight weeks since the last administration of infliximab
   - at least four weeks since the last administration of adalimumab oretanercept

7. History of positive response defined as an ACR20 or a DAS 28 decrease ≥ 1.2 or by the investigator opinion with previous TNFα antagonist treatment.

8. Secondary treatment failure to maximum one previous TNFα antagonist treatment as defined by:

   - Investigator opinion.

     OR

   - DAS28 increase ≥ 0.6 during the last six months.

     OR

   - Decrease in European League Against Rheumatoid (EULAR) score.

9. If the patient is female, she must be of non-childbearing potential, i.e., either surgically sterilized or one year post-menopausal; or, if of childbearing potential, she must have used adequate contraceptive precautions (e.g., intrauterine contraceptive device, oral contraceptives, diaphragm or condom in combination with contraceptive jelly, cream or foam) for 30 days prior to immunization, have a negative pregnancy test (serum) and must agree to continue such precautions during the whole study period .

10. Written informed consent obtained from the patient.

4.4. Exclusion criteria for enrolment

(Amended 29 November 2010)

Patients meeting any of the following criteria will be excluded from participation in the study:

1. Treatment with non-biological DMARDs within four weeks prior to first study product administration. MTX is allowed provided it is administered at a stable dosage ≤ 20mg/week since at least 4 weeks.

2. Treatment with any rheumatoid arthritis biological therapy other than TNFα antagonists at any time prior to first study product administration.

3. Administration of high doses of intra-articular corticosteroids for the treatment of an acute mono-arthritis (e.g. knee) within 3 months prior to first study product administration. High dose of corticosteroids is defined as >50 mg triamcinolone or equivalent)

4. History of documented severe bacterial infection within 28 days prior to first immunization.

5. History of primary resistance or intolerance to any TNFα antagonist.

6. History of or current congestive heart failure, controlled or not.

7. Corticosteroids (prednisone, or equivalent, ≤10 mg per day) are allowed if they are administered at stable dosage since at least 4 weeks prior to the first immunization. Inhaled and topical steroids are allowed.

8. Known history of tuberculosis (TB).

9. Suspicion of TB at chest X-rays at screening or within three months prior to first administration of study product.

10. Suspicion of latent or active tuberculosis as defined by:

    - Positive Mantoux/Purified Protein Derivative (PPD) test (≥5mm induration measured 48 to 72 hours after intradermal injection of tuberculin) at screening or within one year prior to first administration of study product.
    - and/or positive interferon-γ (IFN γ) TB diagnostic test (as measured by the ELISpot method) at screening or within 30 days prior to first administration of study product.

11. Positive for Human Immunodeficiency Virus (HIV), Hepatitis C Virus (HCV) or Hepatitis B Virus (HBV) including Hepatitis B surface Antigen (HBsAg) and anti-Hepatitis B core (anti-HBc) antibodies.

12. Use of any investigational or non-registered product (drug or vaccine) other than the study product(s) within 30 days preceding the first dose of study product(s), or planned use during the study period.

13. Administration of any live vaccine within three months prior to first administration of study product (e.g. oral poliomyelitis vaccine, measles-mumps-rubella vaccine, yellow fever vaccine, rotavirus vaccine, varicella vaccine, zoster vaccine, Bacillus Calmette-Guérin (BCG) vaccine, oral typhoid vaccine).

14. Any confirmed or suspected immunosuppressive or immunodeficient condition.

15. Pregnancy, lactation or planned pregnancy during the study.

16. History of malignancy, except surgically treated cutaneous basal cell carcinoma

17. History of allergic disease or reactions likely to be exacerbated by any component of the study product, including seafood allergy

18. Current serious neurologic or mental disorders.

19. Acute disease at the time of enrolment (acute disease is defined as the presence of a moderate or severe illness with or without fever). The study product can be administered to persons with a minor illness such as diarrhea, mild upper respiratory infection with or without low-grade febrile illness, i.e. Oral or Axillary temperature <37.5°C.

20. Acute or chronic, clinically significant pulmonary, cardiovascular, hepatic or renal functional abnormality as determined by physical examination or laboratory tests, at the discretion of the Investigator.

21. Abnormal laboratory values, in particular:

    - Hemoglobin < 10g/dl
    - White blood cells (WBC) count < 3.0×10^9/L.
    - Platelet count < 100×10^9/L.
    - Serum creatinine level >145µmoles/L.
    - Serum Alanine amino transferase (ALT) or Aspartate amino transferase (AST) >2.5ULN.
    - Alkaline phosphatases >2.5ULN.
    - OR any other abnormality that is clinically relevant according to the Investigator’s opinion.

22. History of chronic alcohol consumption and/or drug abuse.

4.5. Elimination criteria during the study

(Amended 29 November 2010)

The following criteria should be checked from Visit 3 to EOS. If any become applicable during the study, it will not require withdrawal of the patient from the study but may compromise a patient’s evaluability in the Per Protocol Set (PPS) analysis:

- Use of any investigational or non-registered product (drug or vaccine) other than the study product during the study period.

- The requirement of initiating or modifying before month 3 the dose of any anti-rheumatism treatment used and permitted at entry, including methotrexate, corticosteroid and non steroidal anti inflammatory drugs. Inhaled and topical steroids are allowed**.**Administration of high doses of intra-articular corticosteroids (triamcinolone, or equivalent above 50 mg per injection) for the treatment of an acute mono-arthritis (e.g. knee) before Month 3.

- Use of any non-biological DMARD (including MTX >20mg/week) before the month 3 visit.
- Use of any biological therapy before the Month 3 visit.
- Administration of immunoglobulins and/or any blood products during the study period before the Month 3 visit.
- Drug and/or alcohol abuse.

4.6. Contraindications to subsequent TNF-K/control administration

(Amended 29 November 2010)

The following adverse events (AEs) or conditions constitute absolute contraindications to further administration of TNF-K. If any of these AEs or conditions occurs during the study, the patient must not receive additional doses of TNF-K but must continue other study procedures. The patient must be followed until resolution of the event, or condition:

- Anaphylactic reaction following the administration of study products.
- Any confirmed or suspected immunosuppressive or immunodeficient condition.
- Suspected or overt tuberculosis or other severe infection in the investigator's opinion.
- Administration of any live vaccine (e.g. oral poliomyelitis vaccine, measles-mumps-rubella vaccine, yellow fever vaccine, rotavirus vaccine, varicella vaccine, zoster vaccine, BCG, oral typhoid vaccine) before the month 3 visit. Afterwards, only TNF-K recipients will avoid these vaccines until study end.
- Pregnancy.

The following AEs constitute contraindications to administration of study products at that point in time. If any one of these AEs occurs at the time scheduled for immunization, the patient may be immunized at a later date, within the time window specified in the protocol (see Table 3) or withdrawn at the discretion of the Investigator. The patient must be followed upuntil resolution of the event, as with any AEs.

- Acute disease at the time of immunization. Acute disease is defined as the presence of a moderate or severe illness with or without fever. All study products can be administered to persons with a minor illness such as diarrhea, mild upper respiratory infection with or without low-grade febrile illness, i.e. Oral or Axillary temperature <37.5°C.

5. CONDUCT OF THE STUDY

5.1. Patient identification

Patients will be screened for eligibility for the study. For each patient who signs the informed consent form (ICF), the investigator will call the IVRS to request a screening number (a unique identification number). The screening number will consist of 6 digits (two digits to represent the country, 2 to represent the site and 2 to represent the patient's order of inclusion at the site).

Patients will be identified by their unique identification number throughout the study.

5.2. Outline of study procedures

(Amended 26 November 2010)

Table 2 List of study procedures

Visits		1	2 *Baseline*	3	4	5	6	7	8	9	10	11	12	13[a]	Extended F-*UP* [b]
Timing	Days	-30 to -1	0	7	17	28	38	56	84	112	140	168	252	336	
	Months		0			1		2	3	4	5	6	9	12	15, 18,
Initiation procedures	Informed consent	● c													
	Check eligibility criteria	●													
	Medical history	●													
	Mantoux/PPD test and/or TB IFNγ ELISpot [d]	● c													
	Chest X-Ray [d]	● c													
12-lead ECG		● c												●	
IVRS calls[e]		●	● *	(●)**		● **		● **	●		● f **			● g **	●
Randomization			● *												
Check contraindications			●	(●)		●									
Physical examination	*P*hysical examination	●	●	(●)	●	●	●	●	●			●	●	●	●
	Disease specific physical exam / *Clinical scores*	●	●						●			●	●	●	●
	Complaint directed physical exam		●	(●)	●	●	●								
Vital signs (Pulse rate, blood pressure, *Body temperature)* (pre- and 1h post immunization)			●	(●)		●									
TNF-K administration (two schedules)			●	(●)		●									
Check elimination criteria				(●)	●	●	●	●	●	●	●	●	●	●	
Rescue therapy (optional)									●	●	●	●	●	●	●
Blood sampling	Hematology and biochemistry	●		●	●	●	●		●			●	●	●	●
	Viral serology (HBV, HCV, HIV)	● c													
	Antibodies (anti-TNFα, anti-KLH)		●		●	●	●	●	●	● h	● h	● h	● h	● h	●
	Neutralizing antibodies		●					●			● h		● h	● h	
	ADA	●						● f			● f				
	Serum/plasma cytokines		●					●	●		●	●	●	●	
	T cell response [i]		●					●			●			● g	
	RA markers (serology, inflammation)		●						●			●	●	●	●
	Serum pregnancy test	●													
Urine pregnancy test			●	(●)		●									
Record any concomitant medication/vaccination		●	●	●	●	●	●	●	●	●	●	●	●	●	●
Adverse events (AEs) Serious adverse events (SAEs)	Daily recording of solicited AEs by patients (Day 0 to 6 after immunization) *Diary Card A*		●	(●)		●									
	Recording of unsolicited AEs by patients *Diary Card B*		●	●	●	●	●	●	●	●	●	●	●		
	Return of Diary Card A			●	(●)		●								
	Return of Diary Card B			●	●	●	●	●	●	●	●	●	●		
	Recording of non-serious AEs by Investigator	●	●	●	●	●	●	●	●	●	●	●	●	●	●
	Reporting of SAEs	●	●	●	●	●	●	●	●	●	●	●	●	●	●

[*] 1-3 calendar days prior to Day 0 [**]1-3 calendar days prior to the visit

[a] If a patient is prematurely withdrawn from the study (premature EOS) prior to Visit 13, all evaluations at Visit 13 should be performed

[b] Patients who have received the active treatment and whose anti-TNFα antibody titers have not decreased to ≤ the cut off levels at Month 12 will be asked to come back every 3 months until their anti-TNFα antibody titers decline to ≤ cut off levels (see Section 5.3.16 for procedures to be performed at each additional visit).

[c] Informed consent may be given prior to D-30. Any screening procedures carried out more than 30 days prior to the first immunization must be repeated with the, exception of the Mantoux test provided the previous test was done within 365 days prior to first immunization and was negative, of the ECG provided the previous ECG was done within 84 days prior to first immunization and did not show any clinically significant abnormalities, of the chest X-Ray provided the previous chest X-Ray was done within 3 months prior to first immunization and did not show any clinically significant abnormalities, and of the viral serology provided the previous viral serology was done within 84 days prior to first immunization and was negative.

[d] Mantoux/PPD test/ TB IFNγ ELISpot /chest X-Ray only if requested/allowed by National Health authorities. For Mantoux/PPD test reading should be 48 to 72 hours after the intradermal injection..

[e] IVRS calls to be performed just before or during the visits (please refer to Section 5.3.7 for a detailed description of the IVRS call timings).

[f] Titration only if positive at previous visit.

[g] If Tcell response is positive at Day 56 or Day 140

[h] Blood for these analyses should not be taken in control group patients after unblinding of their stage

[i] In selected countries only

● is used to indicate a study procedure that requires documentation in the individual CRF.

(●) indicates procedures to be performed only for patients assigned to schedule D0-D7-D28.

Note: An analysis of selected endpoints will be performed after all patients assigned to Stage 3 have completed their Month 3 (or premature EOS) visit.

If a patient withdraws from the study before the Month 12 visit, the Month 12 visit procedures will be conducted.

It is the Investigator's responsibility to ensure that the intervals between visits/contacts are strictly followed.

Table 3 Time window between study visits

Visit	*Allowed window*
3 (D7)	D0 + 7days [+/- 1 day]
4 (D17)	D0 +17days [+/- 2 days]
5 (D28)	D0 +28 days [+/- 2 days]
6 (D38)	D0 +38days [+/- 3 days]
7 (D56)	D0 +56 days [+/- 3 days]
8 (D84)	D0 +84 days [+/- 7 days]
9 (D112)	D0 +112 days [+/- 7 days]
10 (D140)	D0 +140 days [+/- 7 days]
11 (D168)	D0 + 168 days [+/- 7 days]
12 (D252)	D0 + 252 days [+/- 7 days]
13 (D336)	D0 + 336 days [+/- 7 days]
FUP1 (D426)*	D0 + 426 days [+/- 7 days]
FUP2 (D516)*	D0 + 516 days [+/- 7 days]

***** *if required***

5.3. Detailed description of study procedures

When materials are provided by Neovacs, it is MANDATORY that all clinical samples (including serum samples) will be collected and stored using exclusively those materials in the appropriate manner. The use of other materials could result in the exclusion of the patient from the PPS analysis (see Section 9.5 for definition of study sets to be evaluated). The Investigator must ensure that his/her personnel and the laboratory(ies) under his/her supervision comply with this requirement. However, when Neovacs does not provide material for collecting and storing clinical samples, then appropriate materials from the Investigator's site are to be used.

5.3.1. Screening Visit (Visit 1)

(Amended 29 November 2010)

A patient may be eligible after screening but may not immediately proceed to the immunization phase due to the staggered design of the study. Immunization may be initiated up to 30 days after initial screening. If more than 30 days have elapsed since initial screening, all screening procedures must be repeated (and only the latter results should be recorded in the CRF) with the exception of:

- the Mantoux test, provided the previous test was done within 365 days prior to first immunization and was negative,

- the ECG, provided the previous ECG was done within 84 days prior to first immunization and did not show any clinically significant abnormalities,
- the chest X-Ray, provided the previous chest X-Ray was done within 3 months prior to first immunization and did not show any clinically significant abnormalities,
- the viral serology, provided the previous viral serology was done within 84 days prior to first immunization and was negative.

5.3.2. Informed consent

(Amended 03 May 2010)

The volunteers will attend the pre-study recruitment session at which they will be interviewed and examined by the Investigator (either the Principal Investigator (PI) or the PI's nominee) and given full information regarding the nature and requirements of the study, including an "Information for Patients and Consent Form for Study Participation" At this time, the patient volunteers will be asked to sign an ICF. Participants will be given a copy of the signed ICF for their records. The signed and dated originals will be held by the Investigator in the patient files.
No study procedures will commence until the patient has given informed consent to participate in the study.

5.3.3. Eligibility criteria

(Amended 03 May 2010)

At the Screening Visit, after informed consent has been given, all applicable inclusion and exclusion criteria as described in Sections 4.3 and 4.4 must be checked before enrolment.

A patient is considered as included (enrolled) once he/she has been randomized. Patients who provide informed consent but are not randomized will be considered as screen failures.

5.3.4. Medical history

(Amended 03 May 2010)

A history-directed medical examination will be performed during the Screening Visit (Visit 1). Any pre-existing conditions or signs and/or symptoms present in a patient prior to the start of the study will be recorded in the CRF. Treatment of any abnormality observed during this examination has to be performed according to local medical practice outside this study or by referral to an appropriate health care provider.

5.3.5. Tuberculosis testing

(Amended 29 November 2010)

The following tuberculosis (TB) diagnostic tests will be performed at the Screening Visit (Visit 1):

- Any history of TB disease
- One or both of the following tests, as per local standard practice:
  - TB skin screening test (Mantoux/PPD test), if required/allowed by the National Health Authorities with a reading 48 to 72 hours after the test was performed. The Mantoux/PPD test will not need to be repeated if the previous test was performed within 365 days prior to the first immunization and was negative.
  - IFNγ TB diagnostic test (ELISpot).
- Chest X-Ray if allowed/ required by National Health Authorities. The Chest X-Ray will not need to be repeated if the previous chest X-Ray was performed within 3 months prior to the first immunization and did not show any clinically significant abnormalities.

5.3.6. Physical examination and vital signs

(Amended 03 May 2010)

Physical examination

A full physical examination will be performed at all visits except at visits 9 and 10 and will comprise measurements of body height (at screening only), body weight, routine medical examination of body systems (cardiovascular system [CVS], pulmonary, abdominal assessments).

The CVS assessment includes a 12-lead electrocardiogram (ECG) recording at entry into the study and at Month 12 or end of study (EOS) if prior to Month 12. The ECG and the automatic printout of ECG parameters will be retained with the source notes. The ECG will not need to be repeated if the previous ECG was performed within 84 days prior to the first immunization and did not show any clinically significant abnormalities.

Prior to and post study products administration (1 h), blood pressure (systolic and diastolic) and pulse rate will be measured after the patient has been in a supine position for three minutes. Oral temperature will also be measured.

Disease specific physical examination / Clinical scores
A disease specific physical examination will be performed at Visit 1, 2, 8, 11, 12 and 13 (and follow-up, if applicable) in order to calculate the clinical scores related to the evolution of RA in patients (see Appendix 1).

The following clinical scores will be used:

- American College of Rheumatoid (ACR20 ACR50 and ACR70) criteria.

- Disease Activity Scale 28 (DAS28) score.
- EUropean League Against Rheumatoid (EULAR) criteria.

Please, refer to Appendix 1 for a detailed description of these scores.

The Tender Joint Count (TJC) and the Swollen Joint Count (SJC) will be assessed using the same 28-joint counts in shoulders, elbows, wrists, metacarpophalangeal joints, proximal interphalangeal joints and knees.

Complaint directed physical examination

An injection site assessment will be performed before and after the injections and also at Visits 4 and 6. The intensity of local AEs at the injection site will be determined by visual assessment of erythema/redness, inflammation/swelling and by asking the patient about his/her perception of itching/pain sensation and tenderness (see Table 10 for intensity grading scale). The longest diameter of the affected skin area will be measured by the study personnel using either a transparent plastic foil ruled in millimeter squares or a ruler. In addition, evaluations at the study center will include measurements of induration, swelling, nodule and regional lymphadenopathy (for regional lymphadenopathy, the presence or absence, and location of axillary, subclavicular or cervical nodes should be noted as well as any unexpected findings).

5.3.7. IVRS calls

(Amended 29 November 2010)

The IVRS calls will be performed 1-3 calendar days before (randomization, immunization, Visit 7, Visits 10 and 13) or during visits (screening, Visit 8, extended follow-up visits) and events (screening failure, EOS).

The exact timings of IVRS calls to be performed throughout the study are stated in Table 4. The appropriate timing related to the EOS will be determined for each patient, according to the situations described in the aforementioned table.

Table 4 Exact timing of the IVRS calls *(Amended 29 November2010)*

Events		Exact timing of the IVRS calls	
		1-3 *calendar* days before event	During event
Visit 1 (screening)			●
Screening failure			●
Visit 2 (Day 0)		●	
Visit 3 (Day 7)		(●)	
Visit 5(Day 28)		●	
Visit 7 (Day 56)		●	
Visit 8 (Day 84)			●
Visit 10 (Day 140)		●	
Visit 13 (Day 336)		● [1]	
Extended Follow-up visits every 3 months			●
Premature end of study [2]	Lost to FUP/withdrawal of consent, etc		●

1. If the T cell response was positive at the Day 56 or Day 140.
2. A separate EOS call will not be necessary for patients who do not prematurely discontinue the study. In particular, the IVRS will inform the sites when anti-TNFα antibody titers have declined to ≤ cut off levels. If the titers have not declined to ≤ cut off levels prior to D336, the IVRS will inform the sites when the patient no longer needs to be followed up (based on the results of the D336 analysis and extended follow-up analyses, if applicable).

In addition, the IVRS system can be used to confirm reception of a treatment box, to unblind a treatment or to request a replacement treatment box.

5.3.8. Randomization

(Amended 03 May 2010)

One to three calendar days prior to the first immunization visit (Visit 2, Day 0), randomization will be performed as explained in Section 6.5.

5.3.9. Contraindications

(Amended 03 May 2010)

Contraindications listed in Section 4.6 will be checked at the beginning of each immunization visit.

5.3.10. TNF-K administration

The study products will be administered intramuscularly according to schedule allocation at Visit 2, Visit 3, and Visit 5 (Days 0, 7, 28) or only at Visit 2 and Visit 5 (Days 0, 28).

For patients entering the TNF-K 90 and 180 mcg group, one injection site will be considered at each injection day. For patients entering the TNF-K 360 mcg group, two injection sites will be considered at each injection day. They must be rotated as described in Table 9 and will be recorded in the Case Report Form (CRF.)

The patients will be observed for at least 60 minutes following the administration of study products, with appropriate medical treatment readily available in case of a rare anaphylactic reaction.

5.3.11. Elimination criteria

(Amended 03 May 2010)

From Visit 3 to EOS, elimination criteria listed in Section 4.5 will be checked. If the patient has to be eliminated from the analysis of the PPS, this must be recorded in the electronic CRF.

5.3.12. Blood sampling

Please refer to Table 2 and Table 5 for the timings and methods performed throughout the study.

5.3.12.1. Biochemistry and hematology

(Amended 03 May 2010)

Routine laboratory parameters will be assessed and reviewed by a Physician to evaluate and confirm the patient's eligibility at screening (see Section 4.4). Patients who have out of range laboratory test results within 10% of the normal range may be included in the study at the discretion of the Investigator.

Afterwards, biochemistry and hematology assessments outlined in Table 5 will be performed throughout the study for safety monitoring (see Section 7.3.3).

Routine laboratory parameters include:

- Blood chemistry: ALT, AST, Lactate Dehydrogenase (LDH), Creatine Phosphokinases (CPK), Alkaline phosphatases, Serum creatinine, Urea.
- Hematology: Platelet count, RBC, WBC (including differential count), Hemoglobin, Hematocrit, Prothrombin time, Erythrocyte Sedimentation Rate (ESR), CRP (at screening only).

5.3.12.2. Viral serology

(Amended 03 May 2010)

The following serological assessments will be performed at the Screening Visit to test the viral status of the patients:

- HBsAg and anti-HBc antibodies.
- HCV antibodies.
- HIV.

The viral serology will not need to be repeated if the previous viral serology was performed within 84 days prior to the first immunization and did not show any clinically significant abnormalities.

5.3.12.3. Immunological assessments

(Amended 29 November 2010)

The following immunological assessments, also outlined in Table 5, will be performed throughout the study according to the timings stated in Table 2:

- Anti-TNFα antibodies.
- Anti-KLH antibodies.
- Serum Cytokines: Interleukin 6 (IL-6), IL-17, IL-23
- TNFα in serum and/or plasma
- TNFα soluble receptor 2 levels in plasma
- ADA: antibodies against the TNFα previously received by the patient.
- T cell response: lymphoproliferation assessment and titration of IL-2 and IFN-γ in supernatant. In selected countries only.

The following immunological assessments will be performed only if an anti-TNFα antibody response, defined as a three fold increase in the pre-treatment level, has been observed at the appropriate sampling time:

- TNF neutralizing antibodies.
- Immunoglobulin (Ig) isotypes (IgG, IgA, IgM, IgE) and Ig subclasses (IgG1, IgG2, IgG3, IgG4).

5.3.12.4. RA markers assessments

The following RA markers will be assessed:

Serology:

- Rheumatoid Factor (RF).
- Anti-Cyclic Cirullinated Peptides (anti-CCP) antibodies.
- Matrix MetalloProteinases (MMP1 and -3).

Inflammation:

- C-Reactive protein (CRP).

5.3.13. Pregnancy test

A serum pregnancy test will be performed at the Screening Visit (Visit 1) whereas urine pregnancy tests will be performed prior to each study products administration.

Performance of any additional urine pregnancy tests during interim visits and follow-up will be left at the Investigator's discretion.

5.3.14. Recording of concomitant medication

(Amended 03 May 2010)

Any concomitant medication taken within 30 days prior to the first immunization and until EOS will be recorded in the CRF .

5.3.15. Recording of non-serious adverse events (AEs) and serious adverse events (SAEs)

(Amended 03 May 2010)

Patients will be provided with two diary cards to record details of any AEs experienced until the next visit (see Section 7.2.1). The first diary card will be used the day of the immunization and the 6 following days to record any solicited AEs. The second diary card will be used to record any unsolicited AEs experienced from the day of the immunization until the next visit. At each visit, the Investigator will record any AEs that have occurred since the last visit.

The patients will be instructed to contact the Investigator immediately if they experience any signs or symptoms which they perceive as serious. Please refer to Section 7.4 for SAE reporting procedures.

5.3.16. Interim analysis and study conclusion

(Amended 03 May 2010)

The interim analysis will be performed at Month 3 when all patients assigned to any of the three stages have completed the Month 3 visit or been prematurely withdrawn from the study. The Month 3 analysis will include data for the evaluation of the primary and selected secondary endpoints.

The final analysis for all safety, immune response, and clinical efficacy endpoints will be performed at the end of the study i.e. when all patients have completed *the* Month 12 study visit.

Patients who have received the active treatment and whose anti-TNFα antibody titers have not *decreased to ≤ the cut off levels* at Month 12 will be asked to return every three months until anti-TNFα antibody titers have *decreased to ≤ the cut off*. At each visit, the following procedures will be performed:

- Physical examination.

- Blood sample for antibody detection, hematology, biochemistry and RA markers.
- Clinical scores.
- Recording of any AEs.
- Recording of any concomitant medication/vaccination.

An additional analysis will be performed when all patients have completed the extended follow-up.

5.4. Sample handling and analysis

Samples will not be labeled with information that directly identifies the patients but will be coded with the identification number for the patient.

Collected samples may be used for purposes related to the quality assurance of the laboratory tests described in this protocol. This may include the management of the quality of these current tests, the maintenance or improvement of these current tests, the development of new test methods for the markers described in this protocol, as well as making sure that new tests are comparable to previous methods and work reliably.

It may be that any findings in the present or in other studies necessitate further investigation by Neovacs into the efficacy or immune responses of TNF-K and its constituents under study or further research in the study of RA. Under these circumstances, additional testing on the samples may be performed by Neovacs outside the scope of this protocol.

Any sample testing will be done in line with the consent of the individual patient.

Any human pharmacogenetic testing will require specific consent from the individual patients and the ethics committee approval.

Collected samples will be stored for up to 15 years (counting from when the last patient performed the last study visit), unless local rules, regulations or guidelines require different timeframes or different procedures, which will then be in line with the patient consent. These extra requirements need to be communicated formally to and discussed and agreed with Neovacs.

5.4.1. Sample volumes and handling

(Amended 03 May 2010)

The maximum volume of blood collected from each patient during the study will not exceed 500 mL.

The volumes of blood to be drawn at each visit are indicated in Table 5. Further details on blood treatment procedures will be stated in the Laboratory Manual.

5.4.2. Laboratory assays

(Amended 03 May 2010)

Routine laboratory safety parameters (serology at screening only, hematology, coagulation and clinical chemistry) will be analyzed by the hospital laboratory using standardized collection tubes and procedures.

Other parameters will be assessed in Neovacs designated laboratories according to the procedures described in the Laboratory manual.

The tests to be conducted throughout the study are outlined in the Laboratory Manual.

Table 5 Laboratory assays *(Amended 29 November 2010)*

Read Out	Marker	Method	Blood Volume	Sample type	Laboratory
**Hematology**	Complete blood cell count and differential (RBC, WBC, Platelets)	Standard laboratory procedures	5 ml	Whole blood	Investigator's laboratory
	Hemoglobin				
	Hematocrit				
	Prothrombin time				
	Erythrocyte Sedimentation Rate (ESR)				
	C-Reactive Protein (CRP) (screening only)				
**Biochemistry**	Alanine Aminotransferase (ALT)	Standard laboratory procedures	2.5 ml	Serum	
	Aspartate Aminotransferase (AST)				
	Lactate dehydrogenase (LDH)				
	Creatine phosphokinases (CPK)				
	Alkaline phosphatases				
	Serum creatinine				
	Urea				
**Viral Serology**	HBs Ag, Anti-HBc Ab	Standard laboratory procedures	1 ml	Serum	
	HCV Ab				
	HIV				
**Tuberculosis**	IFN-gamma	ELISpot (Quantiferon Gold)	5 ml	Whole blood	
**Pregnancy test**	Serum β-HCG	Standard laboratory procedures	0.5 ml	Serum	
	Urine β-HCG			Urine	
**Antibodies**	Anti-TNFα antibodies	ELISA - Luminex	20 ml*	Serum	Neovacs' designated laboratory**
	Anti KLH antibodies				
**N Antibodies**	TNF Neutralizing antibodies		20 ml*	Serum	
**TNF-R II**		ELISA - Luminex	2 ml	Plasma	
**TNFα**		MSD	20 ml*	Serum	
**Serum cytokines**	IL-6, IL-17, IL-23	Multiplex -ELISA	20 ml*	Serum	
**T cell response**	Lymphoproliferation	Tritiated thymidine incorporation	40 ml	Whole Blood	
	Cytokines in supernatant (IL-2, IFNγ)				
**RA markers**	Rheumatoid factor (RF)	EIA / ELISA	20 ml*	Serum	
	Pro-Matrix MetalloProteinases (pro-MMP-1 and-3)				

Read Out	Marker	Method	Blood Volume	Sample type	Laboratory
	Anti-CCP antibodies				
	C-Reactive Protein (CRP)	Standard			
**ADA**	Antibodies against TNFα antagonists	FIDIS™ , RIA	20 ml*	Serum	

* A maximum total of 20ml of blood will be drawn per visit for all these analyses

** See Laboratory Manual for more details and for exact volumes of blood and types of samples at each visit

5.5. Restrictions

5.5.1. Prior and concomitant medications

(Amended 03 May 2010)

The medications in Table 6 are allowed prior to the first study product administration and during the study.

Table 6 Permitted medication *(Amended 29 November 2010)*

Medication	Minimum treatment duration at stable dosage prior to first study drug administration	Maximum dose authorized
Methotrexate	4 weeks	20 mg/week,
Corticosteroids	4 weeks	10 mg/day
NSAIDs	2 weeks	NA

The disallowed medications prior to and during the study, together with the minimum wash-out periods, are indicated in Table 7.

Table 7 Disallowed medications and wash-out periods *(Amended 29 November 2010)*

Disallowed medication	Wash-out period
Any other investigational or non-registered product (drug or vaccine)	30 days prior to first immunization
Non-biological DMARDs (including MTX if >20mg/week)	4 weeks prior to first immunization
Infliximab	8 weeks prior to first immunization
Etanercept, Adalimumab	4 weeks prior to first immunization
Certolizumab, Golimumab	10 weeks prior to first immunization
Biological RA therapy other than TNFα antagonists	Any time prior to first immunization
Corticosteroids (prednisone or equivalent >10mg/day)	3 months prior to first immunization
Live vaccines	3 months prior to first immunization
High dose intra articular corticosteroids	3 months prior to first immunization

5.5.2. Blood donation

Patients should not donate blood or blood products (other than for the purposes of this study) from study entry and until the EOS or until the decline of the antibody response following active immunization with TNF-K.

5.5.3. Clinical trial restrictions

(Amended 03 May 2010)

Participation in another clinical trial is not allowed from one month prior to and until the end of this study or in case of patient withdrawal, until the decline of the anti-TNFα antibody response to ≤ cut off levels.

5.6. Compliance control and monitoring

5.6.1. Patient compliance

(Amended 03 May 2010)

All study products will be administered to the patients by suitably trained clinical staff designated and authorized by the Investigator at the study site. Patients will be required to attend the medical facility for the study required visits at the specified times. Patients unable to comply may be withdrawn after discussion between the Investigator and the medical monitor. Patients are free to withdraw their consent at any time.

5.6.2. Site compliance and monitoring

(Amended 03 May 2010)

In agreeing to participate, the investigator undertakes to strictly comply with the study protocol, GCP and the national regulations. The investigator also guarantees the authenticity of the data collected in the context of the study and agrees to the legal provisions for study sponsor quality control.

In compliance with GCP, the regular onsite verification of the data by the study monitor or other person authorized to conduct study related monitoring.

The investigator undertakes to make him/herself available to the study monitor and provide direct access to source data/documents for study related monitoring, audits, IEC review, and regulatory inspection.

Sites will be monitored by the Neovacs' designated Contract Research Organization (CRO). The purpose of site monitoring is to verify that:

- the rights and well-being of human patients are protected
- the reported trial data are accurate, complete and verifiable from source documents
- the conduct of the trial is in compliance with the currently approved protocol, with Good Clinical Practice (GCP) and with the applicable regulatory requirements.

Monitoring will be performed during the following on-site visits:

- Pre-study visit
- Initiation visit

- Interim monitoring visits
- Study end visit

Additional contacts may take place by telephone, fax, letter or email outside of the on-site visits.

5.6.3. Medical monitoring

The Medical Monitor will represent Neovacs in the event of questions regarding patient eligibility, evaluation of AE's, major and minor protocol deviations, and questions relating to the protocol and conduct of the study.

Tel: +33 (0)1 53 10 26 40<br>Fax: +33(0)1 53 10 93 03<br>Mobile phones for 7/7 day availability: +32(0)472 96 00 17

5.6.4. Pharmacovigilance

(Amended 03 May 2010)

The Medical Monitor will work in collaboration with the CRO United BioSource Corporation (UBC Geneva) on the pharmacovigilance aspects according to their standard procedures and the GCP Guidelines.

Tel: +41 22 596 44 44<br>Fax: +41 22 596 44 46

5.6.5. Independent Data and Safety Monitoring Board

This trial is overseen by an IDSMB operating under a charter.

An independent committee consisting of experts in the appropriate disciplines has been appointed to oversee ethical and safety aspects of the study conduct. The role of the IDSMB includes the review of the implementation and progress of the study. It provides advice on safety-related issues which is based on the interpretation of study data with reference to the study protocol. The IDSMB is empowered to suspend the enrolment to the trial and/or further product administration pending review of potential safety issues arising in this trial.

6. INVESTIGATIONAL PRODUCT AND ADMINISTRATION

6.1. Study products

(Amended 29 November2010)

The study products, TNF-K and control, have been developed and manufactured by Neovacs.

The Quality Control Standards and Requirements for TNF-K and control are described in separate release protocols and the required approvals have been obtained.

All study materials, prepared in compliance with current Good Manufacturing Practice (cGMP) requirements and guidelines for injectable products, will be provided by Neovacs and are listed in Table 8.

Table 8 Study products

Study product	Formulation/vial	Presentation	Volume*
TNF-K 90 mcg	180 mcg of TNFα conjugated with KLH	Freeze-dried pellet in monodose vial to be reconstituted with water for injection and ISA-51	0.3 mL per injection time
TNF-K 180 mcg	180 mcg of TNFα conjugated with KLH	Freeze-dried pellet in monodose vial to be reconstituted with water for injection and ISA-51	0.6 mL per injection time
TNF-K 360 mcg	180 mcg of TNFα conjugated with KLH	Freeze-dried pellet in monodose vial to be reconstituted with water for injection and ISA-51	2 vials: 0.6 mL x2 administered separately per injection time
Control	30 mg of mannitol	Freeze-dried pellet in monodose vial to be reconstituted with water for injection and ISA-51	According to the corresponding TNF-K dose
Adjuvant	ISA-51	3 mL Liquid in 8 mL vials	
Liquid for reconstitution	WFI	2 mL Liquid in 2 mL monodose vials	

* volume for injection after reconstitution

Each monodose vial of TNF-K or control are to be reconstituted in 0.3 mL of water for injection (WFI) and 0.3 mL of ISA-51 adjuvant, as described in Section 6.3.

6.2. Dosage and administration

(Amended 29 November2010)

The study products will be injected by suitably trained clinical staff.
Administration methods of TNF-K and control throughout the study are outlined in Table 9.

Table 9 Dosage and administration of study products

Timing	Injection	Group	Study product	Route	Site	Side	Location
Stage 1	1	TNF-K 90 mcg	½ of TNF-K 180 mcg + WFI + ISA-51	IM	D	R	U
		Control	½ of mannitol + WFI + ISA-51	IM	D	R	U
	2	TNF-K 90 mcg	½ of TNF-K 180 mcg + WFI + ISA-51	IM	D	L	U
		Control	½ of mannitol + WFI + ISA-51	IM	D	L	U
	3*	TNF-K 90 mcg	½ of TNF-K 180 mcg + WFI + ISA-51	IM	D	R	Lo
		Control	½ of mannitol + WFI + ISA-51	IM	D	R	Lo
Stage 2	1	TNF-K 180 mcg	TNF-K 180 mcg + WFI + ISA-51	IM	D	R	U
		Control	Mannitol + WFI + ISA-51	IM	D	R	U
	2	TNF-K 180 mcg	TNF-K 180 mcg + WFI + ISA-51	IM	D	L	U
		Control	Mannitol + ISA-51	IM	D	L	U
	3*	TNF-K 180 mcg	TNF-K 180 mcg + WFI + ISA-51	IM	D	R	Lo
		Control	Mannitol + WFI + ISA-51	IM	D	R	Lo
Stage 3	1	TNF-K 360 mcg	TNF-K 180 mcg + WFI + ISA-51	IM	D	R	U
			TNF-K 180 mcg + WFI + ISA-51	IM	D	L	U
		Control	Mannitol + WFI +ISA-51	IM	D	R	U
			Mannitol + WFI + ISA-51	IM	D	L	U
	2	TNF-K 360 mcg	TNF-K 180 mcg + WFI + ISA-51	IM	D	R	Lo
			TNF-K 180 mcg + WFI + ISA-51	IM	D	L	Lo
		Control	Mannitol + WFI + ISA-51	IM	D	R	Lo
			Mannitol + WFI + ISA-51	IM	D	L	Lo
	3*	TNF-K 360 mcg	TNF-K 180 mcg + WFI+ ISA-51	IM	D	R	B
			TNF-K 180 mcg + WFI+ ISA-51	IM	D	L	B
		Control	Mannitol + WFI + ISA-51	IM	D	R	B
			Mannitol + WFI + ISA-51	IM	D	L	B

IM: Intramuscular, D: Deltoid, R/L: Right / Left, U/Lo: Upper / Lower, B: Back
* If applicable, according to schedule allocation (Days 0, 7, 28 or Days 0, 28).
WFI- water for injection

6.3. Packaging and labeling

(Amended 03 May 2010)

All clinical trial packaging and labeling operations will be performed according to standard practice. Importation of the product will be performed by Neovacs or designated CRO. The contents of the label will be in accordance with all applicable regulatory requirements.

The study products and the materials will be provided to the investigational site packed in one box with appropriate vials and outer-packaging labels and a "Directions for use" leaflet.

6.4. Storage, reconstitution and return

(Amended 03 May 2010)

Upon receiving the study products, the Investigator will dispense the study products only to the identified participants of this study, following the procedures described in this study protocol and documented in the appropriate CRFs.

Study products will be stored away from light and in a securely locked area accessible only to authorized personnel until they are administered to the patients. The site will maintain a temperature log to ensure that they are stored within the correct temperature range. The study products will be stored refrigerated (2-8 °C). The study products must be left at room temperature for at least 6 hours and no more than 24 hours prior to preparation. The emulsion will be ideally prepared extemporaneously, immediately prior to use.

The standard procedure for the reconstitution of TNF-K and emulsion preparation will be communicated to each investigational site. Briefly, the procedure for one emulsion preparation comprises the following steps:

1) Reconstitute the TNF-K freeze dried pellet by injecting 0.3 mL WFI using a 1mL Braun Injekt-F® syringe (provided in the kit) connected to an emulsion needle 20G (provided in the kit). Rotate the vial gently for three minutes to ensure a complete solubilization.

2) Take 0.3 mL of ISA-51 from the ISA-51 vial using the same syringe and needle.

3) Discharge the syringe content into the TNF-K vial solution.

4) Pump up and down the total vial content (ca. 0.6 mL). Avoid pumping air during this operation (tilt the vial and place the needle tip on the bottom of the vial wall). Repeat this operation at least 30 times.

5) Finally, pull up 0.6 mL of the emulsified contents, change the emulsion needle for the IM injection needle 23G prior to administration. Remove air (purging).

6) The emulsion is ideally prepared extemporaneously prior to use but, if necessary, the prepared emulsion may be stored for 10 hours at 2-8°C. In this last case, warm up the emulsion between hands before the injection.

For Stage 1 (90 mcg group), half of a fully reconstituted dose of kinoid in a volume of 0.3 ml will be administered per injection day.

For Stage 2 (180 mcg group), one fully reconstituted dose of kinoid in a volume of 0.6 ml will be administered per injection day.

For Stage 3 (360 mcg group) two fully reconstituted doses of kinoid in a volume of 0.6 ml each will be administered separately per injection day.

For the control formulation, reconstitution and emulsion procedures will remain the same in each stage, but the kinoid powder will be replaced by mannitol powder.

The Investigator or designated deputy will maintain records of the delivery of the study products to site, the use by each patient and the return to Neovacs of unused study products. These records will include receipt, administration, return dates, quantities, lot numbers, product name, vial numbers and the numbers of the vials allocated to the patients. The Investigator or designated deputy will maintain records that adequately document that the patients were provided with the dose specified by the protocol and reconcile all investigational products received from Neovacs. After completion of the

study, all unused study products will be returned to Neovacs, unless otherwise requested by Neovacs in writing.

6.5. Treatment allocation and randomization

(Amended 03 May 2010)

A total of up to 48 patients will be enrolled in the study and randomized in a 3:1 ratio between the TNF-K group and the control group within each stage. Randomized patients dropping out or receiving rescue treatment prior to Day 84 may be replaced upon discussion between the International Coordinating Investigator and the Sponsor. Patients not receiving their assigned number of injections may also be replaced. The patients discontinuing the study after Month 3 will not be replaced.

When the first eight patients are randomized in Stage 1, the recruitment in stage 1 will be temporarily stopped and will only continue in that stage if at least 3 of this initial group of patients have a positive anti-TNFα antibody response at Day 38, defined as a three fold increase as compared to cut off

Randomization of patients may be split between two or three stages due to the staggered method of enrolment (enrolment in Stage 2 and Stage 3 will start when three patients of the previous stage have received their first injection since at least 7 days with no safety issues). Randomization between two stages in parallel (Stage1:Stage2 or Stage2:Stage3) will be performed in a 4:2 ratio until all patients have been randomized to one of the dose stages (Stage 1 or Stage 2 respectively). In case of randomization between three stages (Stage1:Stage2:Stage3), patients will be randomized in a 4:2:1 ratio until all patients have been randomized to one of the dose stages.

An IVRS will manage randomization to the different stages and injection schedules. Patient replacement will also be managed by the IVRS.

6.5.1. Randomization of supplies

(Amended 03 May 2010)

A packaging list will be computer-generated using a standard SAS® (Statistical Analysis System) program and will be used to number the treatment boxes.

TNF-K and control doses will be distributed to each study center.

Each treatment box (one vial of kinoid or of mannitol, one vial of WFI, one vial of ISA-51, one syringe and two needles) will be identified by a unique treatment number. One treatment number will be assigned to a subject for one injection per visit at dose levels 90 and 180 mcg and two treatment numbers will be assigned for two injections per visit at dose level 360 mcg.

The initial shipment will consist of one control and one TNF-K treatment boxes and the first resupply to a site will be triggered following the first randomization at the site. Additional resupplies will be triggered if a patient is randomized and the treatment available at the site is insufficient to cover the planned treatment period for all currently

randomized patients at the site and 4 additional treatment boxes (two controls and two TNF-K boxes).

6.5.2. Randomization of patients

(Amended 29 November 2010)

The treatment allocation at the Investigator site will be performed using central randomization via an IVRS. Randomization lists computer-generated using a standard SAS® program will be integrated in the IVRS.

At the time of randomization, the IVRS will assign the patient to a treatment arm and schedule and one (90 and 180 mcg) or two (360 mcg) treatment numbers corresponding to one or two kinoid or mannitol treatment boxes for the first injection(s). The IVRS will also assign treatment numbers to patients continuing treatment at D7 and/or D28 as per the assigned treatment schedule. The actual treatment numbers administered to the patient will be recorded in the ***CRF***.

6.6. Method of blinding and breaking the study blind

Data will be collected in a double-blinded manner within dose of study product until all patients randomized to the dose have completed Month 3 or have been prematurely withdrawn from the study. This implies that during this period, the patients and those responsible for the evaluation of any study endpoints (e.g. safety and efficacy) will be unaware of the identity of the product (TNF-K or control) administered to a particular patient. The dose of study product used in each phase (90, 180 or 360 mcg) will however be open at any moment.

If emergency unblinding is considered necessary for a patient prior to the planned unblinding for his/her dose level, the Investigator or person designated by the Investigator, should contact Neovacs or UBC Safety physician to discuss the need for emergency unblinding. The randomization code will be broken only if the Investigator in charge of the patient estimates that medical events cannot be treated without knowing the identity of the study product administered to his/her patient.

Unblinding procedures will be managed via the IVRS.

In the case of a support line call to the randomization team requesting a treatment unblinding, the support line operator will request the identity of the person and ask for confirmation that they are a qualified doctor requesting the unblinding for a medical emergency. If confirmation is given, the support line operator will be authorized to unblind the treatment.

**Study Contacts for Emergency Code Break**	**Neovacs Clinical Safety Physician**
Toll free numbers:<br>France - 0 800 801 269<br>Belgium - 0800 71 227<br>Argentina -0800 666 30 54<br>Bulgaria – 00800 110 33 10<br>Croatia - 0800 75 39	Tel: +33153102640

Study Contacts for Emergency Code Break	Neovacs Clinical Safety Physician
Chile – 1230 020 7700<br>Romania - +40 318 10 7447	
Fax: +33140219495	Fax: +33153109303
Back-up phone: +33140211909	Mobile phones for 7/7 day availability: +32472960017

7. ADVERSE EVENTS AND SERIOUS ADVERSE EVENTS

(***Amended 03 May 2010***)

All TNF-K patients will be followed until the anti-TNFα antibody response declines to ≤ cut off levels and at least until Month 12. Control group patients will be followed until Month 12.

The Investigator is responsible for reporting all adverse events (AEs) that are observed or reported, regardless of their relationship to study product from the time a patient gives informed consent until study completion.

7.1. Definitions

7.1.1. Adverse event

ICH E6 GCP Guidelines define an AE as any untoward medical occurrence in a patient or subject administered a pharmaceutical product in a clinical investigation regardless of its causal relationship to the study treatment.

An AE can therefore be any unfavorable and unintended sign (including an abnormal laboratory finding), symptom or disease temporally associated with the use of medicinal (investigational) product. The occurrence of an AE may come to the attention of study personnel during study visits and interviews or by a study recipient presenting for medical care.

All AEs must be graded for intensity and relationship to study product.

7.1.2. Intensity of event

(Amended 03 May 2010)

All AEs will be assessed by the Investigator using the following definitions:

- **Mild**: events require minimal or no treatment and do not interfere with the subject's daily activities.
- **Moderate**: events result in a low level of inconvenience or concern with the therapeutic measures. Moderate events may cause some interference with functioning.

- **Severe**: events interrupt a subject's usual daily activity and may require systemic drug therapy or other treatment. Severe events are usually incapacitating.

The overall duration of each AE and the number of days at the maximum severity will be documented in the CRF. AEs characterized as intermittent require documentation of onset and duration (overall and by maximum severity) of each episode.

7.1.3. Relationship to study products

The Investigators will decide if AEs are related to the administered products. The assessment of causality will be made using the following definitions:

- **Unrelated**: This category is applicable to AEs which are judged to be clearly and incontrovertibly due to extraneous causes (disease, environment, etc.) and do not meet the criteria for drug relationship listed under Unlikely, Possible or Probable.

- **Unlikely**: In general, this category is applicable to an AE which meets the following criteria (must have the first two):

  1. It does not follow a reasonable temporal sequence from administration of the drug.
  2. It may readily have been produced by the subject's clinical state, environment or toxic factors, or other modes of therapy administered to the subject.
  3. It does not follow a known pattern of response to the suspected drug.
  4. It does not reappear or worsen when the drug is re-administered.

- **Possible**: This category applies to AEs in which the connection with the investigational product administration appears unlikely but cannot be ruled out with certainty. An AE may be considered possible if, or when (must have the first two):

  1. It follows a reasonable temporal sequence from administration of the drug.
  2. It may have been produced by the subject's clinical state, environment or toxic factors, or other modes of therapy administered to the subject.
  3. It follows a known pattern of response to the suspected drug.

- **Probable**: This category applies to AEs which are considered to be related to the investigational product with a high degree of certainty. An AE may be considered probable, if (must have the first three):

  1. It follows a reasonable temporal sequence from administration of the drug.

2. It cannot be reasonably explained by the known characteristics of the subject's clinical state, environment or toxic factors, or other modes of therapy administered to the subject.

3. It disappears or decreases on cessation or reduction in dose.

4. It follows a known pattern of response to the drug.

5. It reappears on re-challenge.

7.1.4. Serious adverse event

A **serious adverse event** (SAE) is defined as an AE meeting one of the following conditions:

- Death.
- Life threatening event (defined as any adverse event that places the subject, in the view of the Investigator, at immediate risk of death from the event as it occurred, i.e., it does not include an event that had occurred in a more severe form, might have caused death).
- An event requiring inpatient hospitalization or prolongation of existing hospitalization during the period of protocol defined surveillance.
- Results in a persistent or significant disability/incapacity.
- Congenital anomaly or birth defect in the offspring of a study participant.
- Any other important medical event that may not result in death, be life threatening, or require hospitalization, may be considered a serious adverse experience when, based upon appropriate medical judgment, the event may jeopardize the subject and may require medical or surgical intervention to prevent one of the outcomes listed above. Examples of such medical events include allergic bronchospasm requiring intensive treatment in an emergency room or at home, blood dyscrasias or convulsions that do not result in inpatient hospitalization, or the development of drug dependency or drug abuse.

7.2. Adverse event recording

(Amended 03 May 2010)

Each AE occurring to a subject, either spontaneously revealed by the subject or observed by the Investigator, whether believed by the Investigator to be related or unrelated to the study product, must be recorded on the AE information page of the CRF and on the subject's hospital notes.

The Investigator will determine the relationship of any AEs to investigational product and record it on the appropriate section of the CRF.

7.2.1. Diary cards

(Amended 03 May 2010)

Patients will be provided with two diary cards. These will include the definitions of mild, moderate and severe AEs, as described in Section 7.1.2, in order to facilitate the assessments by the patients of their own level of functional impairment for each experienced AE.

The first diary card will be used to record on the day of the immunization and the 6 following days the presence or absence, and severity of the following solicited AEs:

- Local AEs, at each injection site:
  - Pain, tenderness, itching with grading of severity.
  - Swelling, redness, induration, ulceration, with measurement of size in mm.
- General AEs:
  - Temperature in °C.
  - Vomiting, nausea, fatigue, myalgia, headache with grading of severity.

In addition, patients will be asked to record on the second diary card any other unusual symptoms (unsolicited AEs) experienced during that period.

After the seven day period following each TNF-K/control administration, patients will be asked to record on the second diary card any unusual symptoms (solicited and unsolicited AEs) experienced until the next hospital visits.

Patients will be instructed to bring their diary cards with them to the unit when they return for their visits. Following discussion of symptoms with the Investigator, information on local tolerability/AEs will be recorded in the CRF, as appropriate. Diary cards will be retained with the subject's source notes after completion.

7.2.2. *Recording of* solicited adverse events *by study staff*

(Amended 03 May 2010)

The presence and intensity of general AEs occurring within one hour after injection will be recorded by the study staff, in particular malaise, fatigue, temperature, gastro intestinal symptoms and headache.

The presence and intensity of local AEs at the injection site will also be determined by study center staff during the first hour after study treatment administration by visual assessment of erythema/redness, inflammation/swelling and by asking the subject about his/her perception of itching/pain sensation and tenderness (see Table 10 for intensity grading scale). The longest diameter of the affected skin area will be measured using either a transparent plastic foil ruled in millimeter squares or a ruler. In addition, evaluations at the study center will include measurements of induration, swelling, itching,

nodule and regional lymphadenopathy (for regional lymphadenopathy, the presence or absence, and location of axillary, subclavicular, cervical nodes should be noted as well as any unexpected findings).

7.3. Assessment of AE intensity

All local and general AEs will be graded according to the intensity scales described below.

7.3.1. Local reactions

(Amended 03 May 2010)

Local reactions will be reported and graded according to the intensity scale in Table 10.

Table 10 Intensity scale for local reactions

Local Reaction	Mild (Grade 1)	Moderate (Grade 2)	Severe (Grade 3)
**Pain**	Does not interfere with activity	Interferes with activity	Prevents daily activity
**Tenderness**	Mild pain to touch	Pain with movement	Significant pain at rest
**Erythema/Redness***	>0 to <30 mm	≥30 to <120 mm	≥120 mm
**Inflammation/Swelling***	>0 to <30 mm	≥30 to <120 mm	≥120 mm
Induration	***>0 to <30 mm***	***≥30 to <120 mm***	***≥120 mm***
Itching	***Mild or localized***	***Intense or widespread***	***Prevents daily activity***
**Inflammation/Swelling****	Does not interfere with activity	Interferes with activity	Prevents daily activity

*Measure at greatest single diameter
**Inflammation/Swelling are to be evaluated using both the quantitative and subjective scales mentioned in the table.

7.3.2. Systemic reactions

Quantitative systemic reactions will be reported and graded according to the intensity scale in Table 11.

Table 11 Intensity scale for quantitative systemic reactions

Systemic Reaction	Mild (Grade 1)	Moderate (Grade 2)	Severe (Grade 3)
**Fever (°C)***	≥37.5 to <38	≥38 to <39	≥39
**Respiratory rate, breaths per minute****	20 to 23	24 to 29	≥30
**Tachycardia (beats per minute)****	101 to 115	116 to 130	≥131
**Bradycardia (beats per minute)**	<45 to ≥40	<40 to ≥35	<35
**Hypertension, systolic (mmHg)**	151 to 165	166 to 175	≥176
**Hypertension, diastolic (mmHg)**	91 to 95	96 to 100	≥101
**Hypotension, systolic (mmHg)**	89 to 85	84 to 80	≤79
**Vomiting**	1 to 2 episodes in 24 hours	>2 episodes in 24 hours	Requires intravenous hydration

*Oral temperature; no recent hot or cold beverages or smoking. [Note: A fever can be considered not product-related if an alternative etiology can be checked and documented by the Investigator.]
** Subject at rest for 3 minutes, supine, prior to measurement.

Subjective systemic reactions will be reported and graded according to the intensity scale in Table 12.

Table 12 Intensity scale for subjective systemic reactions

**Systemic Reaction (subjective)**	**Mild (Grade 1)**	**Moderate (Grade 2)**	**Severe (Grade 3)**
**Headache**	No interference with activity	Some interference with activity	Significant, prevents daily activity
**Fatigue**	No interference with activity	Some interference with activity	Significant, prevents daily activity
**Myalgia**	No interference with activity	Some interference with activity	Significant, prevents daily activity
**Nausea**	No interference with activity	Some interference with activity	Significant, prevents daily activity

7.3.3. Reactions assessed by laboratory parameters

(Amended 03 May 2010)

AEs assessed by laboratory parameters will be reported and graded according to the intensity scale in Table 13.

The laboratory normal ranges are only illustrative and will be adjusted for the actual hospital laboratory ranges.

Table 13 Intensity scale of reactions assessed by laboratory parameters

Laboratory	Laboratory Normal Range	Mild (Grade 1)	Moderate (Grade 2)	Severe (Grade 3)
**Hemoglobin (female) – g/dL**	11.5-14.8	<11.5 and ≥10.0	<10 and ≥8.5	<8.5
**Hemoglobin (female) – change from *screening* value in g/dL**	-	≥1.5 and <2.0	≥2.0 and <3.0	≥3.0
**Hemoglobin (male) – g/dL**	13.0-16.7	<13.0 and ≥11.0	<11.0 and ≥10.0	<10.0
**Hemoglobin (male) – change from *screening* value in g/dL**	-	≥1.5 and <2.0	≥2.0 and <3.0	≥3.0
**White blood cells - Increase – cells/µL**	3,500 – 10,000	≥10, 000 and <15,000	≥15,000 and <20,000	≥20,000
**White blood cells - Decrease – cells/µL**	3,500 – 10,000	<3,500 and ≥3, 000	<3,000 and ≥2,000	<2,000
**Lymphocytes - Decrease – cells/µL**	800 – 4,000<br>15.5 – 46.6%	<800 and ≥600	<600 and ≥400	<400
**Neutrophils - Decrease – cells/µL**	1,500 – 8,000<br>40 – 75%	<1,500 and ≥1,250	<1,250 and ≥1,000	<1,000
**Eosinophils - %**	0.0 – 7%	≥7 and <10	≥10 and <15	≥15
**Platelets - Decrease – cells/µL**	400,000 - 140,000	<140,000 and ≥100,000	<100,000 and ≥75,000	<75,000
**Liver function tests (increase by factor)**	-	>1.0 and <2.5 x ULN	≥2.5 and <5 x ULN	≥5 x ULN
**Serum creatinine – µmol/L**	60-130	>130 and <145	≥145 and <160	≥160

Abbreviations: ULN = upper limit of normal

7.4. Adverse event *and pregnancy* reporting

(Amended 03 May 2010)

Adverse event reporting

AEs including local and systemic reactions should be captured on the appropriate CRF page. Information to be collected includes event description, Investigator assessment of severity, relationship to study product, seriousness, and outcome. All AEs, regardless of relationship to study products, must be appropriately documented. All AEs will be followed up until adequate resolution.

Any medical condition that is present at the time that the patient gives informed consent should be considered as pre-existing and not reported as an AE. However, if it deteriorates at any time during the study, it should be recorded as an AE.

All SAEs will also be:

- Recorded on the relevant SAE reporting form and followed up through to resolution by a study physician,
- Submitted on an SAE form to the Pharmacovigilance Department of United BioSource Corporation (UBC) (see Section 5.6.4 for contact details),
- Then, submitted by UBC to Neovacs.

All SAEs, regardless of relationship, will be reported via fax by the site within 24 hours of becoming aware of the event.

Other supporting documentation of the event may be requested and should be provided as soon as possible.

All SAEs will be followed up until satisfactory resolution or until the PI or Sub-Investigator deems the event to be chronic or the patient to be stable.

The Sponsor or delegate (Pharmacovigilance Department of UBC) will send appropriate safety notifications to Health Authorities in accordance with applicable laws and regulations.

The Investigator must comply with any applicable site-specific requirements related to the reporting of SAEs (and in particular deaths) involving his/her subjects to the IEC/IRB that approved the trial.

In accordance with ICH GCP guidelines, the Sponsor will inform the Investigator of "findings that could adversely affect the safety of subjects, impact the conduct of the trial or alter the IEC's/IRB's approval/favorable opinion to continue the trial." In particular and in line with respective regulations, the Sponsor will inform the Investigator of AEs that are both serious and unexpected and are considered to be at least possibly related to the administered product ("Suspected Unexpected Serious Adverse Reactions" or SUSARs – the reference safety information for this trial is the product Investigator Brochure). The Investigator should place copies of Safety reports in the Investigator Site File. National regulations with regard to Safety report notifications to Investigators will be taken into account.

When specifically required by regulations and guidelines, the Sponsor delegate (Pharmacovigilance Department of UBC) will provide appropriate Safety reports directly to the concerned lead IEC/IRB and will maintain records of these notifications. When direct reporting by the Sponsor or delegate is not clearly defined by national or site-specific regulations, the Investigator will be responsible for promptly notifying the concerned IEC/IRB of any Safety reports provided by the Sponsor or delegate and of filing copies of all related correspondence in the Investigator Site File.

For trials covered by the European Directive 2001/20/EC, the Sponsor's responsibilities regarding the reporting of SAEs/SUSARs/Safety Issues will be carried out in accordance with that Directive and with the related Detailed Guidances.

Pregnancy reporting

Any pregnancy that occurs during the study must be recorded on a pregnancy notification form and reported to UBC Safety Europe with 24 hours of learning of the occurrence. The pregnancy should be followed up to determine its outcome (i.e. healthy birth, spontaneous or voluntary abortion, presence or absence of any birth defects, congenital abnormality or maternal and/or newborn complications). It should include the investigator assessment of any possible relationship between the outcome and the exposure to study products. Women becoming pregnant while their partner is under treatment with the study products should also be followed up in order to collect

information regarding the pregnancy outcomes. Consent to report information regarding the pregnancy should be obtained from the mother.

7.5. Emergency and rescue medication

(Amended 03 May 2010)

The possibility that TNF-K can cause anaphylaxis in humans cannot be excluded. All participating centers should therefore be equipped to manage cardiovascular resuscitation.

Please refer to Section 6.6 for emergency unblinding and contact details of Neovacs' Clinical Safety Physician.

Rescue therapy, may be provided to any patients from Month 3 if required (before unblinding: any treatment except anti-TNFα therapy; after unblinding: any treatment for the patients of the placebo group and any treatment except anti-TNFα therapy for the patients of the TNF-K group). If a patient has a medical need for rescue treatment prior to the Month 3 visit, the study treatment should be permanently discontinued (if applicable) and rescue treatment administered at the discretion of the investigator. The patient should still be followed up in the study.

8. SUBJECT WITHDRAWAL AND STOPPING RULES

8.1. Withdrawal of patients on the basis of safety

(Amended 03 May 2010)

If any subject withdraws, the reason for a subject withdrawing from the study will be reported in the subject's CRF.

Patients may prematurely discontinue treatment and they may also prematurely withdraw from the study at any time. Patients will discontinue treatment after any administration of investigational product for the following reasons (reason to be recorded on the appropriate page of the CRF):

- The subject is withdrawn according to the stopping rules of TNF-K administration within the study (see Section 8.2).
- Development of an intolerable AE due to study participation, as determined by the Investigator.
- Development of an intercurrent illness, condition or procedural complication, which would require the study treatment to be permanently discontinued.
- Discovery that the subject entered the study in deviation of the protocol or occurrence of a protocol deviation during the study.

- The PI believes it is medically in the best interest of the subject to permanently discontinue the study treatment.
- New data raises concern about the safety of the study treatment so that continuation would pose potential risks to the patients.
- Pregnancy.

Patients prematurely discontinuing the study treatment should still be followed up, where possible, according to the protocol (excluding procedures related to study treatment administration). Patients who discontinue the study prematurely will be given a complete examination as per the assessments planned at the Month 12 visit including a physical examination, and standard safety laboratory tests if possible. All patients who are withdrawn from the study because of AEs or clinical laboratory abnormalities will be followed up at suitable intervals in order to evaluate the course of the AE or laboratory abnormalities and to ensure reversibility or stabilization. The subsequent outcomes of these events will be recorded on the CRF. Reasons for patients withdrawing from the study will be documented in the CRFs in any case.

Patients who discontinue after the first study product administration should be followed until their anti-TNFα antibodies have decreased to ≤ the cut off levels following the same procedures as control patients after Month 3.

Patients who withdraw before Month 3 for non treatment related reasons may be replaced upon discussion between the International Coordinating Investigator and the Sponsor. Patients discontinuing the study after Month 3 will not be replaced.

8.2. Stopping rules on the basis of safety

The administration of TNF-K within the study will be put on hold if:

- One or more patients present a SAE possibly or probably related to the drug according to the investigator.
- Neovacs and/or the IDSMB believe that the number and/or severity of AEs justify halting the study.
- New data raises concerns about the safety of the study material so that continuation would pose potential risks to the patients.

The available safety, immune responses and clinical data will then be reviewed by an IDSMB which may recommend to either resume or to terminate the dose group or the study. The IDSMB recommendations will be submitted to all ECs and national health authorities.

If a group is terminated then the patients in that group will be followed according to the study protocol until the anti-TNFα antibody response declines to ≤ cut off levels.

9. STATISTICAL ANALYSES

Statistical analyses will be performed by an independent CRO.

9.1. Planned analyses

Statistical analyses during the course of this study will include one interim analysis and one final analysis.

The interim analysis will be performed at Month 3 when all patients assigned to any of the three stages have completed their Month 3 visit or been prematurely withdrawn from the study. The analysis will include data until Month 3 for the evaluation of the primary endpoint, selected secondary and safety endpoints.

The final analysis for all safety, immune response, and clinical efficacy endpoints will be performed at the end of the study i.e. when all patients have completed their Month 12 study visit or have been prematurely withdrawn from the study.

9.2. Primary endpoint

(Amended 03 May 2010)

The primary endpoint will be the proportion of patients with at least a 3-fold increase in antibody response to TNFα vs cut off at Day 38 (immune response). It will be analyzed at the interim analysis at Month 3.

Since the immune response induced by active immunization against a self-cytokine is not well defined and may vary from one disease to another, it is not certain that the anti-TNFα antibody response will allow by itself to select the best dose and schedule. Therefore, findings resulting from the analyses of key secondary endpoints, such as the presence and intensity of a neutralizing antibody response, the clinical response, the circulating levels of TNFα and soluble TNFα receptor and the safety parameters, will also be taken into account together with the primary endpoint to determine the best dose and schedule.

9.3. Secondary endpoints

9.3.1. Interim analysis at Month 3

(Amended 29 November 2010)

Clinical efficacy

- Proportion of patients with a ≥1.2 decrease in DAS28 at Month 3 vs baseline (Day 0).
- Absolute change in DAS28 at Month 3 vs baseline (Day 0).

- Absolute change in Swollen Joint Counts (SJC) and in Tender Joint Counts (TJC) at Month 3 vs baseline (Day 0).

- Proportion of patients achieving ACR20, ACR 50 and ACR 70 at Month 3 vs baseline (Day 0).

- Proportion of patients with a good/moderate EULAR response at Month 3 vs baseline (Day 0).

- Absolute change in CRP level at Month 3 vs baseline (Day 0).

- Absolute change in ESR at month 3 vs screening.

- Proportion of patients with or without ADAs at screening with a ≥1.2 decrease in DAS28 at Month 3 vs baseline ***(Day 0)***.

Immune response

- Proportion of patients with at least a 3-fold increase in antibody response to TNFα vs cut off at Month 2 (Day 56) and at Month 3 (Day 84).

- Proportion of patients with a positive neutralizing antibody response to TNFα.

Safety analysis

- Occurrence, intensity and relationship to TNF-K immunization of any solicited local and general signs and symptoms during a seven-day follow up period (i.e. day of immunization and 6 subsequent days) after each TNF-K injection.
- Occurrence, intensity and relationship to TNF-K immunization of unsolicited local and general signs and symptoms occurring until Month 3.
- Occurrence and relationship to TNF-K immunization of all SAE occurring until Month 3.
- Change from screening to Month 3 of hematological and biochemical levels in all groups.
- Hematological and biochemical levels within or outside the normal ranges in all groups.

9.3.2. Final analysis at Month 12

(Amended 03 May 2010)

Clinical efficacy

- Proportion of patients with a ≥1.2 decrease in DAS28 vs baseline (Day 0).

- Absolute change in DAS28 vs baseline (Day 0).

- Absolute change in Swollen Joint Counts (SJC) and in Tender Joint Counts (TJC) vs baseline (Day 0).
- Proportion of patients with ACR20, ACR50 and ACR70 vs baseline (Day 0).
- Proportion of patients with a good/moderate EULAR response vs baseline (Day 0).
- Absolute change in CRP level and vs baseline (Day 0).
- Absolute change in ESR vs screening.
- Proportion of patients with a ≥1.2 decrease in DAS28 score vs DAS28 score decrease observed under previous treatment with a TNFα antagonist.
- Proportion of patients with the same maximum clinical improvement in terms of DAS28 observed during previous treatment with a TNFα antagonist.
- Absolute change in DAS28 vs DAS28 absolute change observed under previous treatment with a TNFα antagonist.
- Absolute change in SJC and in TJC vs SJC and TJC absolute change under previous treatment with a TNFα antagonist.
- Proportion of patients with ACR20, ACR50 and ACR70 vs status observed under previous treatment with a TNFα antagonist.
- Proportion of patients with a good/moderate EULAR response vs status under previous treatment with a TNFα antagonist.
- Proportion of patients withdrawn for lack of efficacy.

Immune responses

The timings of blood samplings and analyses are specified in Table 2 and Table 5.

- Anti-TNFα antibody concentrations.
- Proportion of good responders defined as patients with dilution of anti-TNFα antibodies titers ≥2000
- Proportion of patients with a positive anti-TNFα neutralizing antibody response.
- Anti-TNFα neutralizing antibody levels.
- Anti-KLH antibody concentrations.
- Absolute changes in levels of cytokines (TNFα, TNF-RII, IL-6, IL-17, IL-23 and others) vs baseline (Day 0).

- Proportion of patients with a positive T cell response as measured by lymphoproliferation.

Safety

- Occurrence, intensity and relationship to TNF-K immunization of unsolicited local and general signs and symptoms occurring throughout the study period.
- Occurrence and relationship to TNF-K immunization of all SAE occurring throughout the study period.
- Change from screening in hematological and biochemical levels in all groups.
- Hematological and biochemical levels within or outside the normal ranges in all groups.

9.4. Estimated sample size

Assumptions regarding the expected proportions of patients with at least a 3-fold increase in antibody response to TNFα vs cut off at Day 38 are presented in the table below for each dose*injection schedule combination:

Schedules	Doses			
	90 mcg	180 mcg	360 mcg	Control
Day 0 and Day 28	5%	60%	80%	0%
Day 0, Day 7 and Day 28	10%	75%	95%	0%

Statistical power computations are performed with regards to the comparison of the above proportions between the expected optimal dose*schedule injection combination and control. The significance level is set to 0.05/6 = 0.008% to account for multiple comparisons between doses.

A total of 12 patients in the treated group and 12 patients in the control group will achieve 80% power to detect a difference of 75% between group proportions of patients with at least a 3-fold increase in antibody response to TNFα vs cut off at Day 38, using a two-sided Fisher's Exact test. Thus, based on 12 patients in each dose group, 48 patients would have to be included in the study.

9.5. Analysis populations

(Amended 03 May 2010)

Safety Set

The Safety Set (SAF) will include all patients who received at least one dose of study product. Analyses on the SAF will be performed according to the product actually received.

Full Analysis Set

The Full Analysis Set (FAS) will include all randomized patients who received at least one dose of study product and for whom data concerning immune responses endpoint measures are available at Month 3.

Analyses on the FAS will be performed according to the randomization group regardless of the study product actually received.

Per Protocol Set

The Per Protocol Set (PPS) will be a subset of the FAS including all randomized patients who met all inclusion and exclusion criteria, did not meet any elimination criteria during the study and complied with the procedures defined in the protocol (i.e. who did not have protocol deviations that could have an impact on the primary criterion evaluation).

Reasons for exclusion from the PPS will be further described in the SAP.

Populations used for statistical analyses

The analysis of the primary endpoint and selected clinical efficacy and immune response secondary endpoints at interim analysis at Month 3 will be primarily performed on the PPS (due to the exploratory nature of the efficacy evaluations at this phase II stage of the development plan) and then on the FAS.

The analysis of clinical efficacy secondary endpoints vs baseline (or vs screening (ESR)) at the final analysis at Month 12 will be primarily performed on the PPS and then on the FAS.

The analysis of all other secondary clinical efficacy and immune response endpoints at the final analysis at Month 12 will be performed on the FAS.

The analysis of the safety secondary endpoints will be performed on the SAF, both at the interim analysis at Month 3 and at the final analysis at Month 12.

Some results (disposition of patients, data availability etc.) will be presented on all randomized patients.

9.6. Statistical considerations

(Amended 03 May 2010)

Statistical analyses will be performed using the SAS® software (SAS Institute, Cary, NC, USA).

A first version of the statistical analysis plan (SAP) will be written before the interim analysis. It will detail the statistical methodology to be applied for the analysis of the primary and key secondary endpoints. A second version will be finalized prior to the Month 3 study database lock detailing all analyses to be performed, the rules and conventions used to compute the questionnaire scores, the derived analysis variables and how to handle missing data. The second version of the SAP will also include the list and content of the statistical tables, figures, and listings presenting the results. All

amendments to the first version of the SAP will be documented with justifications to protect the integrity of the trial.

Continuous data will be summarized using the number of observations, mean, 95% confidence interval (CI) of the mean (unless specified otherwise), standard deviation, median, 25% and 75% quartiles, minimum and maximum. Categorical data will be presented in contingency tables along with frequencies, percentages and their 95% CI (unless specified otherwise).

The 95% CIs will be calculated using:

- the normal approximate method for GMT and other continuous endpoints,
- the exact binomial distribution for percentages.

Unless specified otherwise in the SAP (multiple comparisons), statistical tests will be two-sided at a 5% significance level.

Medications will be coded using the WHO Drug dictionary. Medical history and adverse events will be coded using the Medical Dictionary for Regulatory Activities (MedDRA).

All study data will be at least presented in individual patient data listings.

9.7. Statistical Methods

The same statistical methods will be applied both at the interim and at the final analysis. They are described in the next sections.

9.7.1. Demographic, baseline and follow-up characteristics

Demographic, baseline and follow-up characteristics such as medical history, previous medications and concomitant medications during the study will be summarized by treatment group on the SAF and the FAS by means of descriptive statistics. Formal statistical comparisons between treatment groups will be performed with regards to age.

9.7.2. Immune responses

Primary endpoint

The primary endpoint will be the proportion of patients with at least a 3-fold increase in antibody response to TNFα vs cut off at Day 38.

The cut-off level of the ELISA to detect anti-TNFα antibodies during the study will be calculated as twice the mean of the baseline dilutions values in the phase 1-2 study TNF-K-001. At the end of the study, this cut-off level will be updated for the analysis by calculating the mean of the baseline dilutions in the present trial.

The following statistical methodology will be followed:

First, the presence of an overall vaccine effect will be assessed by testing whether there is any differences across the dose*injection schedule combinations. Comparisons will be

performed by means of a non parametric Analysis of Variance (ANOVA), with anti-TNFα antibody concentrations at Day 38 as response variable and dose*injection schedule combinations as factor. Age will be taken into account as a covariate in the analysis.

Testing and adjustment of type I error for multiple comparisons between combinations will be performed using the "multiple comparison with the worst" method described by Hsu (1996). This method controls type I error (overall alpha level of 5%) and will allow to determine optimal dose*schedule injection combinations. No adjustment for interim and final analyses will be performed as the primary endpoint will be analyzed at the interim analysis only.

Then, the proportion of patients with at least a 3-fold increase in antibody response to TNFα vs ***cut off*** at Day 38 will be compared by means of a Fisher test between the control combinations and the optimal dose*injection schedule combination determined previously.

The presence and nature (either quantitative or qualitative) of any interaction between doses and injection schedules will be explored by means of a non parametric ANOVA with anti-TNFα antibody concentrations at Day 38 as response variable, doses and injection schedules as main factors, the dose*injection schedule interaction term and age as covariate.

In the absence of a statistically significant qualitative interaction, the primary efficacy endpoint will also be analyzed for each of the two main effects, i.e. doses and injection schedules.

The full statistical methodology for the analysis of the primary endpoint will be further described in the SAP.

Secondary endpoints

The proportions of patients with at least a 3-fold increase in antibody response to TNFα vs cut off at Month 2 (Day 56) and at Month 3 (Day 84) will be analyzed at the interim analysis at Month 3 following the same methodology as for the primary endpoint.

Secondary immune response endpoints (proportions of patients, antibody concentrations, GMT for antibodies) will be described with 95% CIs according to dose*injection schedule combinations at each visit a blood sample result is planned.

Depending on the nature of the dose*injection schedule interaction observed for the primary endpoint, additional descriptions could also be performed by dose and by injection schedule.

9.7.3. Clinical efficacy

Descriptive statistics of all clinical efficacy assessments (values at visits) and all secondary clinical efficacy endpoints listed in Section 9.3 (changes from baseline (or screening (ESR)) and proportions of patients) will be computed at each visit according to dose*injection schedule combinations.

For exploratory purposes, between-group comparisons will be performed for efficacy secondary endpoints vs baseline. Adjustment on age will be taken into account in the analyses. Patients dropping out or receiving rescue treatment prior to Month 3 may be replaced upon discussion between the International Coordinating Investigator and the Sponsor. Patients discontinuing the study after Month 3 will not be replaced. Sensitivity analyses will be performed for all between-group comparisons by replacing missing efficacy data after Month 3 using appropriate imputation techniques that will be described in the SAP.

Depending on the nature of the dose*injection schedule interaction observed for the primary endpoint, additional analyses could also be performed by dose and by injection schedule.

Potential associations between clinical efficacy and immune response endpoints (anti-TNFα antibody concentrations, anti-TNFα neutralizing antibody levels) will also be explored.

All statistical methods applied for the analysis for clinical efficacy endpoints will be further described in the SAP.

9.7.4. Safety

The proportions of patients with at least one local solicited adverse event, with at least one general solicited adverse event and with any solicited adverse event as well as the proportions of patients reporting each individual solicited local and general adverse event during the solicited follow-up period (days 0 to 6) will be tabulated according to dose*injection schedule combinations with 95% CI after each study product dose and overall.

The proportions of patients reporting at least one unsolicited adverse event will be tabulated according to dose*injection schedule combinations based on the MedDRA classification at the Preferred Term (PT) and primary System Organ Class (SOC) levels. Individual patient data listings will also include the Lowest Level Term (LLT).

The same tabulation will be performed for grade 3 solicited and unsolicited adverse events, solicited and unsolicited adverse events related to immunization and for grade 3 solicited and unsolicited adverse events related to immunization.

The proportion of patients reporting SAEs will be presented according to dose*injection schedule combinations and overall.

Descriptive statistics will be presented according to dose*injection schedule combinations and visits for all hematological and biochemical parameters. Out of range laboratory data will be summarized according to dose*injection schedule combinations and visits and the number and proportions of patients with normal and abnormal values at post-baseline visits will be listed with respect to the same categorization at baseline (shift tables).

Depending on the nature of the dose*injection schedule interaction observed for the primary endpoint, additional descriptions could also be performed by dose and by injection schedule.

10. DOCUMENTATION OF DATA

The findings will be evaluated by an independent CRO.

Computations for the statistical methods will be performed using the computer software package SAS® version 8 and higher, or similar validated software.

10.1. *C*ase report forms (*CRF*s)

(Amended 03 May 2010)

The Investigator or a designee will be responsible for recording study data in the CRF provided by Neovacs. It is ultimately the Investigator's responsibility to ensure the accuracy of the data entered in the CRFs.

Clinical data (including AEs and concomitant medications) will be entered into a CRF. Clinical data will be entered into a quality assured database. Clinical data will be entered directly from the source documents.

Data validation will be performed after data entry and verification by computerized logical checks and manual review.

An independent CRO will carry out the data processing in accordance with their data management procedures. Database lock will occur once quality assurance procedures have been completed.

10.2. Data entry in database

(Amended 03 May 2010)

The data entry will be made on site by the study personnel into the CRFs except for laboratory data. In particular, data from local laboratory reports (results and normal ranges) will be double data-entered at the CRO and central laboratory data will be transferred to the CRO from the different centralized laboratories. All laboratory data will be uploaded into the study database.

Procedures for treatment of missing, unused and spurious data will be addressed in the detailed Statistical Analysis Plan (SAP).

10.3. Coding of adverse events, drugs and diseases

After data entry, the AEs will be coded according to MedDRA Version 11.1 or later. Concomitant medication will be coded according to WHO Drug Reference List. Concomitant diseases will be coded according to MedDRA.

10.4. Reporting and communication of results

A report will be generated by Neovacs or delegate at each of the following time-points:

- when all Month 3 data are cleaned and analyzed.
- when all Month 12 data are cleaned and analyzed.
- When all patients have completed extended follow-up, if applicable

11. REGULATORY AND ETHICAL ISSUES

11.1. General legal references

This study will be conducted under the Declaration of Helsinki, revised form of 59th World Medical Association (WMA) General Assembly, Seoul, South Korea, October 2008 (see Appendix 2).

11.2. Insurance and Indemnity

Neovacs has subscribed an insurance for the conduct of this clinical trial in each country

Based on the number of study visits and in order to facilitate the follow-up of patients through the study duration, patients will be compensated for local transportation costs only.

11.3. Ethics committee

(Amended 03 May 2010)

The EC will review the final, approved protocol and the ICF. The composition of the committee will conform to global and local regulations and guidelines and will approve all aspects of the study, including protocol, informed consent form and any modifications made to the protocol or informed consent prior to initiation.

11.4. Protocol adherence - amendments

(Amended 03 May 2010)

The protocol must be read thoroughly and the instructions must be followed exactly.

Any changes in the protocol will require a formal amendment. Such amendments will be agreed upon and approved in writing by the PI and Neovacs. Substantial amendments have to be notified to the EC and the National Health Authorities. Changes that are not substantial, which have no significant impact on the medical or scientific validity of the

study will be documented in a statement. The EC and the National Health Authorities may be notified of administrative changes.

11.5. Required documents

(Amended 03 May 2010)

The PI must provide Neovacs with the following documents prior to the enrolment of any subject (copies should be kept by the Investigator in the appropriate file folders provided):

- Signed copy (original) of the approved protocol.
- Financial Disclosure.
- Curriculum vitae of the Investigator.

11.6. Informed consent

The patients will give their informed consent. The PI will be responsible for obtaining from every subject, prior to his/her participation in the study, an ICF for study participation signed by the subject in accordance with ICH GCP guidelines.

Patients will be fully informed of the nature of the study, the properties and possible side effects of the investigational products and all relevant aspects of study procedures. They may ask questions to the Investigator or the Clinic Staff at any time.

The ICF will be signed and dated by the patients in the presence of an Investigator or designee (according to the site delegation of duties list). Participants will be given a copy of the signed '"Information for Patients and Consent Form for Study Participation" for their records. The signed and dated originals will be held on file by the PI.

11.7. Subject data protection

(Amended 03 May 2010)

Patients will be informed that their clinical data may be sighted by Neovacs' monitor on behalf of Neovacs and by external auditors on behalf of either Neovacs or regulatory agencies. They will similarly be informed that information from the study will be prepared and may also be submitted to National Health Authorities and perhaps for publication. However, participants of the study will only be identified in such reports by their study identification number and perhaps their gender, initials and age. The PI undertakes to hold all personal information in confidence.

11.8. Quality assurance

(Amended 03 May 2010)

The site will be audited as necessary during the course of the study. The audits will include control of adherence to the protocol, Standard Operating Procedures (SOPs), ICH GCP Guidelines, and national laws. Source data verification and checking of data entered in the CRFs will be used for assessment of complete and reliable documentation.

The PI will allow Neovacs or their designated representatives to audit, at mutually convenient time(s) during the study, or after the study has been completed, all CRFs and all corresponding portions of office, clinic, and laboratory records of each study participant.

Regulatory authorities and representatives of the relevant independent EC will be permitted to conduct inspections at the site. The PI should notify Neovacs if the regulatory authority contacts them to schedule an inspection.

11.9. Record retention

All source data, clinical records and laboratory data relating to the study will be retained in the archive of the sponsor for 15 years after the completion of the clinical study.

All correspondence relating to this study should be kept in appropriate file folders. If an Investigator moves, withdraws from an investigation or retires, the responsibility for maintaining the records may be transferred to another person (e.g. Sponsor, other Investigator) who will accept the responsibility. Notice of this transfer must be made to and agreed upon by Neovacs.

11.10. Confidentiality

The CRO and Neovacs will affirm and uphold the principle of the subject's right to protection against invasion of privacy. Throughout this study, all data will only be identified by an identification number and the patient's gender, initials and date of birth.

All information concerning this study and which was not previously published is considered confidential information. This confidential information shall remain the sole property of Neovacs; it shall not be disclosed to others without written consent of Neovacs and shall not be used except in the performance of this study.

The information compiled during the conduct of this clinical study is also considered confidential and may only be disclosed and/or used by Neovacs as they deem necessary. To allow the use of the information derived from this clinical study and to ensure compliance to current global regulations, the Investigator is obliged to furnish Neovacs with the complete test results and all data compiled in this study.

11.11. Publications

The investigator is not entitled to publish the results of the study without the Neovacs' prior written consent, which shall not be unreasonably withheld. Should the investigator desire to publish the results of this study, the investigator will request permission from Neovacs, and provide a copy of the manuscript at least 30 days prior to the expected date of submission to the publisher. Neovacs will review the manuscript and, if Neovacs

consents to publication, will provide any comments on the manuscript to the investigator. The Investigator agrees to include any reasonable comments made by Neovacs and further agrees to delay submission of the manuscript for up to six months if requested by Neovacs. In the event that Neovacs chooses to publish the data from this study, the investigator will be provided with a copy of the manuscript at least 30 days prior to the expected date of submission to the publisher. The investigator will review the manuscript and will provide any comments on the manuscript to Neovacs. Neovacs commits to submit for publication to a peer-reviewed journal the study results within three months following complete analysis of the Month 3 and of the Month 12 data. The results of the analyses will first be discussed with the Investigators. The first author will be the investigator who will have recruited and completed the largest number of patients in the study and the following authors will be the other Investigators in the order of number of recruited patients. Up to three collaborators of Neovacs will be included as authors.

12. REFERENCES

1. Aletaha D, Smolen J. Effectiveness profiles and dose dependent retention rates of traditional disease modifying antirheumatic drugs for rheumatoid: an observational study. J Rheumatol 2002;29:1631-1638.

2. Anderson PJ. Tumor necrosis factor inhibitors: clinical implications of their different immunogenicity profiles. Semin Arthritis Rheum. 2005 Apr;34(5 Suppl1):19-22.

3. Arnett FC, Edworthy SM, Bloch DA, et al. The American Rheumatism Association 1987 Revised criteria for the classification of rheumatoid arthritis. Arthritis Rheum 1988;31:315-24.

4. Baert F, Noman M, Vermeire S, Van Assche G, D' Haens G, Carbonez A, Rutgeerts P. Influence of immunogenicity on the long-term efficacy of infliximab in Crohn's disease. N Engl J Med 2003; 348(7):601-8.

5. Bartelds GM, Wijbrandts CA, Nurmohamed MT, Stapel SO, Lems WF, Aarden L, Dijkmans BAC, Tak PP and Wolbink GJ. Clinical response to adalimumab: the relationship with anti-adalimumab antibodies and serum adalimumab concentrations in rheumatoid arthritis. Ann Rheum Dis 2007; 66(7):921-6.

6. Buch MA, Bingham SJ, Bejarano V, et al. Therapy of patients with rheumatoid arthritis: outcome of infliximab failures switched to etanercept. Arthritis Rheum 2007; 53:448-453.

7. Calahan LF, Pincus T, Huston JW, et al. Measures of activity and damage in rheumatoid arthritis: depiction of changes and prediction of mortality over five years. Arthritis care res 1997;10:381-394.

8. Choy EH, Panayi GS. Cytokine pathways and joint inflammation in rheumatoid arthritis. N Engl J Med 2001;344:907-916.

9. Doran MF, Crowson CS, Pond GR, et al. Frequency of infection in patients with rheumatoid arthritis compared with controls: a population-based study. Arthritis Rheum 2002;46:2287-2293.

10. Drosos A. Epidemiology of Rheumatoid arthritis. Autoimmun Rev 2004;3(Suppl 1):S20-S22.

11. Emery P. Evidence supporting the benefit of early intervention in rheumatoid arthritis. J Rheumatol Suppl 2002;66:3-8.

12. Emery P, Breeveld FC, Lemmel EM, et al. A comparison of the efficacy and safety of leflunomide and methotrexate for the treatment of rheumatoid arthritis. Rheumatology (Oxford). 2000;39:655-665.

13. Felson DT, Anderson JJ, Boers M, et al. American College of Rheumatology preliminary definition of improvement in rheumatoid arthritis. Arthritis Rheum 1995; 38: 727-35.

14. Fransen J, van Riel PLCM. The disease activity score and the EULAR response criteria. Clin Exp Rheumatol 2005;23(suppl 39):S93-99.

15. Fuchs HA, Kaye JJ, Callahan LF, et al. Evidence of significant damage in rheumatoid arthritis within the first 2 years of disease. J Rheumatol 1989; 16: 585-591

16. Keystone E, Kavanaugh AF, Sharp J, et al. Adalimumab (D2E7), a fully human anti-TNFα monoclonal antibody, inhibits the progression of structural damage in patients with active RA despite concomitant methotrexate therapy. Arthritis Rheum 2002; 46(suppl):S205.abstract

17. Le Buanec H, Delavallée L, et al. TNFalpha kinoid vaccination-induced neutralizing antibodies to TNFalpha protect mice from autologous TNF alpha-driven chronic and acute inflammation. Proc Natl Acad Sci U S A. 2006;103(51):19442-7.

18. Lipsky PE, van der Heijde DM, St Clair EW, et al. Infliximab and methotrexate in the treatment of rheumatoid arthritis. Anti tumour necrosis factor trial in rheumatoid arthritis with concomitant therapy study group. N Engl J Med 2000; 1594-1602.

19. Maini R, St Clair EW, Breedveld F, et al. Infliximab (chimeric anti-tumour necrosis factor α monoclonal antibody) versus control in rheumatoid arthritis patients receiving concomitant methotrexate: a randomized phase III trial. ATTRACT study group. Lancet 1999;354:1932-1939.

20. Olsen NJ, Stein CM. New drugs for rheumatoid arthritis. N Engl J Med 2004;350:2167-2179.

21. Prevoo ML, van't Hof MA, Kuper HH, et al. Modified disease activity scores that include twenty-eight joint count. Development and validation in a prospective longitudinal study of patients with rheumatoid arthritis. Arthritis Rheum 1995; 38:44-8.

22. Radstake TRDJ, Svenson M, Eijsbouts AM, et al. Formation of antibodies against infliximab and adalimumab strongly correlate with functional drug levels and clinical responses in rheumatoid arthritis. Ann Rheum Dis 2008;

23. Scott DL, Grindulis kA, Struthers GR, et al. Progression of radiological changes in rheumatoid arthritis. Ann Rheum Dis. 1984;43:8-17.

24. Scott DL, Kingsley GH. Tumor necrosis factor inhibitors for rheumatoid arthritis. N Engl J Med 2006;355:704-712.

25. Scott DL, Pugner K, Kaarela K, et al. The links between joint damage and disability in rheumatoid arthritis. Rheumatology (Oxford) 2000;39:122-132.

26. Sharp JT, Wolfe F, Mitchell DM, Bloch DA. The progression of erosion and joint space narrowing scores in rheumatoid arthritis during the first twenty years of disease. Arthritis Rheum 1991;34:660-668.

27. Smitten AL, Simon TA, Hochberg MC, Suissa S. A Meta-analysis of the incidence of malignancy in adult patients with rheumatoid arthritis. Arthritis research & Therapy 2008; 10:R45.

28. Smolen JS, Kalden JR et al. Efficacy and safety of leflunomide compared with placebo and sulphasalazine in active rheumatoid arthritis: a double-blind, randomised, multicenter trial. European Leflunomide Study Group. Lancet. 1999 Jan 23;353(9149):259-66.

29. Strand V, Cohen S et al. Treatment of active rheumatoid arthritis with leflunomide compared with placebo and methotrexate. Leflunomide Rheumatoid Arthritis Investigators Group. Arch Intern Med. 1999 Nov 22;159(21):2542-50.

30. Tehlirian V, Bathan JM. Rheumatoid arthritis: Clinical and laboratory manifestations. In: Primer on the Rheumatic diseases. Springer 2002.

31. van der Heijden DM, van Leeuwen MA, van Riel PL, et al. Radiographic progression on radiographs of hands and feet duringthe first three years or rheumatoid arthritis measured according to Sharp's scoring method (van der Heijden modification). J Rheumatol 1995;22:1792-1796.

32. van Gestel AM, Anderson JJ, van Riel PL, et al. ACR and EULAR improvement criteria have comparable validity in rheumatoid arthritis trials. American College of Rheumatology European League of Associations for Rheumatology. J Rheumatol 1999; 26: 705-11.

33. van Vollenhoven RF. Clin Exp Rheumatol 2004;22(suppl.35):S115-S121

34. Ward JR. Challenging the pyramid: a new look at therapeutic approaches for rheumatoid arthritis: early intervention with second line therapies. J Rheumatol Suppl 1990;25:18-23.

35. Weinblatt ME, Keystone EC, Furst DE, et al. Adalimumab, a fully human anti-tumor necrosis factor α monoclonal antibody for the treatment of rheumatoid arthritis in patients taking concomitant methotrexate: the ARMADA trial. Arthritis Rheum 2003;48:35-45.

36. Weinblatt ME, Kremer JM, Bankhurst AD, et al. A trial of etanercept, a recombinant tumor necrosis factor receptor Fc fusion protein, in patients with rheumatoid arthritis receiving methotrexate. N Engl J Med 1999;340:253-259.

37. Wolfe F, Freundlich B, Strauss WL. Increase in cardiovascular disease prevalence in rheumatoid arthritis. J Rheumatol 2003;30:36-40.

38. Wolfe F, Mitchell DL,M, Sibley JT, et al. The mortality of rheumatoid arthritis. Arthritis Rheum 1994;37:481-494.

39. Sandborn W, Abreu M, D'haert G, Colombel JF et al. Certolizumab Pegol in Patients With Moderate to Severe Crohn's Disease and Secondary Failure to Infliximab. Clin Gastroenterology and hepatology, 2010 ;8:688–695

Appendix 1 Clinical scores and questionnaire

(Amended 03 May 2010)

The following indicators will be used to assess the evolution of RA's disease in patients:

American College of Rheumatoid 20 (ACR 20), 50 and 70 criteria

These criteria measure 20, 50 or 70 % improvement in tender or swollen joint counts and in three of the following five parameters: acute phase reactant (such as sedimentation rate or CRP), patient disease activity assessment, physician disease activity assessment, patient pain assessment, disability/functional questionnaire (HAQ).

Disease Activity Scale 28 (DAS28) score

The DAS28 score includes in its calculation the following parameters: Tender Joint Counts 28 (TJC 28), Swollen Joint Counts 28 (SJC 28), CRP: C-reactive protein, patient assessment of disease activity.

TJC 28 and SJC 28 should be assessed using the same 28-joint counts (shoulders, elbows, wrists, metacarpophalangeal joints, proximal interphalangeal joints and knees). The CRP should be measured in mg/L. In addition, the patient general health (GH) or global disease activity measured on a Visual Analogue Scale (VAS) of 100 mm must be obtained. Using this data, the DAS28 can be calculated with the following formula:

$$DAS28 = 0.56 \sqrt{(TJC\ 28)} + 0.28 \sqrt{(SJC\ 28)} + 0.{=}36\ [\ln(CRP+1)] + 0.014\ GH + 0.96$$

The DAS28 score provides a value on a scale from 0 to 10, indicating the current activity of the RA of the patient. A DAS28 score above 5.1 means high disease activity whereas a DAS28 score below 3.2 indicates low disease activity. Remission is achieved by a DAS28 lower than 2.6.

European League Against Rheumatoid (EULAR) criteria

The EULAR criteria use comparison of DAS28 scores at two timepoints to assess response or improvement in the evolution of RA in patients. The EULAR response criteria are defined as follows:

**Present DAS28 score**	**DAS28 score improvement**		
	> 1,2	0,6-1,2	<0,6
<3,2	Good response	Moderate response	No response
3,2-5,1	Moderate response	Moderate response	No response
>5,1	Moderate response	No response	No response

Source: http://www.das-score.nl/www.das-score.nl/

Appendix 2 WORLD MEDICAL ASSOCIATION DECLARATION OF HELSINKI

Ethical Principles for Medical Research Involving Human Subjects

Adopted by the 18th **WMA General Assembly, Helsinki, Finland, June 1964, and amended by the:**
29th WMA General Assembly, Tokyo, Japan, October 1975
35th WMA General Assembly, Venice, Italy, October 1983
41st WMA General Assembly, Hong Kong, September 1989
48th WMA General Assembly, Somerset West, Republic of South Africa, October 1996
52nd WMA General Assembly, Edinburgh, Scotland, October 2000
53th WMA General Assembly, Washington 2002 (Note of Clarification on paragraph 29 added)
55th WMA General Assembly, Tokyo 2004 (Note of Clarification on Paragraph 30 added)
59th WMA General Assembly, Seoul, October 2008

INTRODUCTION

The World Medical Association (WMA) has developed the Declaration of Helsinki as a statement of ethical principles for medical research involving human subjects, including research on identifiable human material and data.
The Declaration is intended to be read as a whole and each of its constituent paragraphs should not be applied without consideration of all other relevant paragraphs.

Although the Declaration is addressed primarily to physicians, the WMA encourages other participants in medical research involving human subjects to adopt these principles.

It is the duty of the physician to promote and safeguard the health of patients, including those who are involved in medical research. The physician's knowledge and conscience are dedicated to the fulfillment of this duty.

The Declaration of Geneva of the WMA binds the physician with the words, "The health of my patient will be my first consideration," and the International Code of Medical Ethics declares that, "A physician shall act in the patient's best interest when providing medical care."

Medical progress is based on research that ultimately must include studies involving human subjects. Populations that are underrepresented in medical research should be provided appropriate access to participation in research.

In medical research involving human subjects, the well-being of the individual research subject must take precedence over all other interests.

The primary purpose of medical research involving human subjects is to understand the causes, development and effects of diseases and improve preventive, diagnostic and therapeutic interventions (methods, procedures and treatments). Even the best current interventions must be evaluated continually through research for their safety, effectiveness, efficiency, accessibility and quality.

In medical practice and in medical research, most interventions involve risks and burdens.

Medical research is subject to ethical standards that promote respect for all human subjects and protect their health and rights. Some research populations are particularly vulnerable and need special protection. These include those who cannot give or refuse consent for themselves and those who may be vulnerable to coercion or undue

influence.

Physicians should consider the ethical, legal and regulatory norms and standards for research involving human subjects in their own countries as well as applicable international norms and standards. No national or international ethical, legal or regulatory requirement should reduce or eliminate any of the protections for research subjects set forth in this Declaration.

BASIC PRINCIPLES FOR ALL MEDICAL RESEARCH

It is the duty of physicians who participate in medical research to protect the life, health, dignity, integrity, right to self-determination, privacy, and confidentiality of personal information of research subjects.

Medical research involving human subjects must conform to generally accepted scientific principles, be based on a thorough knowledge of the scientific literature, other relevant sources of information, and adequate laboratory and, as appropriate, animal experimentation. The welfare of animals used for research must be respected.

Appropriate caution must be exercised in the conduct of medical research that may harm the environment.

The design and performance of each research study involving human subjects must be clearly described in a research protocol. The protocol should contain a statement of the ethical considerations involved and should indicate how the principles in this Declaration have been addressed. The protocol should include information regarding funding, sponsors, institutional affiliations, other potential conflicts of interest, incentives for subjects and provisions for treating and/or compensating subjects who are harmed as a consequence of participation in the research study. The protocol should describe arrangements for post-study access by study subjects to interventions identified as beneficial in the study or access to other appropriate care or benefits.

The research protocol must be submitted for consideration, comment, guidance and approval to a research ethics committee before the study begins. This committee must be independent of the researcher, the sponsor and any other undue influence. It must take into consideration the laws and regulations of the country or countries in which the research is to be performed as well as applicable international norms and standards but these must not be allowed to reduce or eliminate any of the protections for research subjects set forth in this Declaration. The committee must have the right to monitor ongoing studies. The researcher must provide monitoring information to the committee, especially information about any serious adverse events. No change to the protocol may be made without consideration and approval by the committee.

Medical research involving human subjects must be conducted only by individuals with the appropriate scientific training and qualifications. Research on patients or healthy volunteers requires the supervision of a competent and appropriately qualified physician or other health care professional. The responsibility for the protection of research subjects must always rest with the physician or other health care professional and never the research subjects, even though they have given consent.

Medical research involving a disadvantaged or vulnerable population or community is only justified if the research is responsive to the health needs and priorities of this population or community and if there is a reasonable likelihood that this population or

community stands to benefit from the results of the research.

Every medical research study involving human subjects must be preceded by careful assessment of predictable risks and burdens to the individuals and communities involved in the research in comparison with foreseeable benefits to them and to other individuals or communities affected by the condition under investigation.

Every clinical trial must be registered in a publicly accessible database before recruitment of the first subject.

Physicians may not participate in a research study involving human subjects unless they are confident that the risks involved have been adequately assessed and can be satisfactorily managed. Physicians must immediately stop a study when the risks are found to outweigh the potential benefits or when there is conclusive proof of positive and beneficial results.

Medical research involving human subjects may only be conducted if the importance of the objective outweighs the inherent risks and burdens to the research subjects.

Participation by competent individuals as subjects in medical research must be voluntary. Although it may be appropriate to consult family members or community leaders, no competent individual may be enrolled in a research study unless he or she freely agrees.

Every precaution must be taken to protect the privacy of research subjects and the confidentiality of their personal information and to minimize the impact of the study on their physical, mental and social integrity.

In medical research involving competent human subjects, each potential subject must be adequately informed of the aims, methods, sources of funding, any possible conflicts of interest, institutional affiliations of the researcher, the anticipated benefits and potential risks of the study and the discomfort it may entail, and any other relevant aspects of the study. The potential subject must be informed of the right to refuse to participate in the study or to withdraw consent to participate at any time without reprisal. Special attention should be given to the specific information needs of individual potential subjects as well as to the methods used to deliver the information. After ensuring that the potential subject has understood the information, the physician or another appropriately qualified individual must then seek the potential subject's freely-given informed consent, preferably in writing. If the consent cannot be expressed in writing, the non-written consent must be formally documented and witnessed.

For medical research using identifiable human material or data, physicians must normally seek consent for the collection, analysis, storage and/or reuse. There may be situations where consent would be impossible or impractical to obtain for such research or would pose a threat to the validity of the research. In such situations the research may be done only after consideration and approval of a research ethics committee.

When seeking informed consent for participation in a research study the physician should be particularly cautious if the potential subject is in a dependent relationship with the physician or may consent under duress. In such situations the informed consent should be sought by an appropriately qualified individual who is completely independent of this relationship.

For a potential research subject who is incompetent, the physician must seek informed

consent from the legally authorized representative. These individuals must not be included in a research study that has no likelihood of benefit for them unless it is intended to promote the health of the population represented by the potential subject, the research cannot instead be performed with competent persons, and the research entails only minimal risk and minimal burden.

When a potential research subject who is deemed incompetent is able to give assent to decisions about participation in research, the physician must seek that assent in addition to the consent of the legally authorized representative. The potential subject's dissent should be respected.

Research involving subjects who are physically or mentally incapable of giving consent, for example, unconscious patients, may be done only if the physical or mental condition that prevents giving informed consent is a necessary characteristic of the research population. In such circumstances the physician should seek informed consent from the legally authorized representative. If no such representative is available and if the research cannot be delayed, the study may proceed without informed consent provided that the specific reasons for involving subjects with a condition that renders them unable to give informed consent have been stated in the research protocol and the study has been approved by a research ethics committee. Consent to remain in the research should be obtained as soon as possible from the subject or a legally authorized representative.

Authors, editors and publishers all have ethical obligations with regard to the publication of the results of research. Authors have a duty to make publicly available the results of their research on human subjects and are accountable for the completeness and accuracy of their reports. They should adhere to accepted guidelines for ethical reporting. Negative and inconclusive as well as positive results should be published or otherwise made publicly available. Sources of funding, institutional affiliations and conflicts of interest should be declared in the publication. Reports of research not in accordance with the principles of this Declaration should not be accepted for publication.

ADDITIONAL PRINCIPLES FOR MEDICAL RESEARCH COMBINED WITH MEDICAL CARE

The physician may combine medical research with medical care only to the extent that the research is justified by its potential preventive, diagnostic or therapeutic value and if the physician has good reason to believe that participation in the research study will not adversely affect the health of the patients who serve as research subjects.

The benefits, risks, burdens and effectiveness of a new intervention must be tested against those of the best current proven intervention, except in the following circumstances:

The use of placebo, or no treatment, is acceptable in studies where no current proven intervention exists; or

Where for compelling and scientifically sound methodological reasons the use of placebo is necessary to determine the efficacy or safety of an intervention and the patients who receive placebo or no treatment will not be subject to any risk of serious or irreversible harm. Extreme care must be taken to avoid abuse of this option.

At the conclusion of the study, patients entered into the study are entitled to be

informed about the outcome of the study and to share any benefits that result from it, for example, access to interventions identified as beneficial in the study or to other appropriate care or benefits.

The physician must fully inform the patient which aspects of the care are related to the research. The refusal of a patient to participate in a study or the patient's decision to withdraw from the study must never interfere with the patient-physician relationship.

In the treatment of a patient, where proven interventions do not exist or have been ineffective, the physician, after seeking expert advice, with informed consent from the patient or a legally authorized representative, may use an unproven intervention if in the physician's judgement it offers hope of saving life, re-establishing health or alleviating suffering. Where possible, this intervention should be made the object of research, designed to evaluate its safety and efficacy. In all cases, new information should be recorded and, where appropriate, made publicly available.

22.10.2008
